# Safety, reactogenicity, and immunogenicity of Ad26.COV2.S co-administered with a quadrivalent standard-dose or high-dose seasonal influenza vaccine: a non-inferiority randomised controlled trial

**DOI:** 10.1016/j.eclinm.2024.103016

**Published:** 2025-01-07

**Authors:** Gabriela Tapia-Calle, Gloria Aguilar, Nathalie Vaissiere, Carla Truyers, Pedro Ylisastigui, Erik Buntinx, Mathieu Le Gars, Frank Struyf, Gert Scheper, Macaya Douoguih, Javier Ruiz-Guiñazú, the COV3005 Study group, Robert Patrizi, Wai Ling, Sanne de Ridder, Marit de Groot, Maria Grazia Pau, Gerald Weidinger, Srividya Pradeep, Nadine Salisch, Sophie Cambre

**Affiliations:** a Janssen Vaccines and Prevention BV, Leiden, the Netherlands; b Janssen Research and Development, Beerse, Belgium; c Alliance for Multispecialty Research, Fort Myers, Florida, USA; d Animal Research Center, Alken, Belgium

**Keywords:** SARS-CoV-2, COVID-19, Vaccine, Adenoviral vectors, Influenza vaccines, Co-administration

## Abstract

**Background:**

Vaccine co-administration can increase vaccination coverage. We assessed the safety, reactogenicity, and immunogenicity of concomitant administration of Ad26.COV2.S COVID-19 vaccine with seasonal influenza vaccines.

**Methods:**

This non-inferiority, Phase 3, randomised, double-blind study enrolled 859 healthy adults and was conducted between 02 November 2021 and 28 November 2022. Participants aged ≥18–64 years were randomised to receive a seasonal quadrivalent standard dose (SD) influenza vaccine (*Afluria* Quadrivalent, Seqirus) concomitantly with Ad26.COV2.S (Coad_SD) or placebo (0.9% NaCl; Control_SD) on Day 1 and placebo or Ad26.COV2.S on Day 29. Participants aged ≥65-years were randomised to the Coad_SD or Control_SD groups, or to Coad_HD or Control_HD groups that received a seasonal quadrivalent HD (high-dose) influenza vaccine (*Fluzone* High-Dose Quadrivalent, Sanofi Pasteur Inc) in the same schedules. The primary outcomes were haemagglutinin inhibition titres against the four influenza vaccine strains at Day 29, and SARS-CoV-2 Spike-specific antibodies at Day 29 in the Coad_SD group and Day 57 in the Control-SD group, with a non-inferiority margin (Control-SD group/Coad_SD group) of 1.5. Reactogenicity and safety were assessed in all participants (NCT05091307).

**Findings:**

Non-inferiority criteria for concomitant administration in the SD groups were met for SARS-CoV-2 Spike-specific antibodies (ratio 1.11, 95% CI 0.97–1.26) and haemagglutinin inhibition titres for all influenza strains (A/H3N2 1.23, 95% CI 1.05–1.45; B/Victoria 0.99, 95% CI 0.84–1.19; B/Yamagata, 1.03, 95% CI 0.88–1.21) except A/H1N1 (1.28, 95% CI 1.09–1.53) for which the upper limit of the 95% CI was >1.5. Concomitant administration of Ad26.COV2.S and SD influenza vaccine induced robust immune responses in terms of SARS-CoV-2 Spike-specific antibodies and haemagglutinin inhibition to all four influenza strains. Seroconversion and seroprotection rates against all influenza vaccine strains were comparable in the Coad and Control groups. Anti-Spike antibodies 28 days after receiving Ad26.COV2.S were similar whether administered with influenza vaccine or alone. Antibody responses persisted at least 6 months post-vaccination in all groups. The reactogenicity and safety profile following co-administration was consistent with the known safety profiles of the study vaccines. No safety concerns were identified. Coadministration was immunogenic and well tolerated in adults aged ≥65 years who received HD influenza vaccine.

**Interpretation:**

Co-administration of seasonal influenza vaccine with Ad26.COV2.S was immunogenic with an acceptable safety profile, supporting co-administration of these vaccines.

**Funding:**

Janssen Vaccines & Prevention BV and 10.13039/100012399Biomedical Advanced Research and Development Authority.

**Research in context**

**Evidence before this study**

We searched PubMed using the terms “COVID-19 influenza vaccine co-administration” for English language articles reporting randomised trials. Three studies in COVID-19 vaccine-naïve individuals observed acceptable reactogenicity and safety, satisfactory immunogenicity, and/or high efficacy against COVID-19 in study participants who received inactivated or protein-based COVID-19 vaccines co-administered with inactivated or adjuvanted seasonal influenza vaccines. Seven studies conducted in booster settings also reported acceptable safety and reactogenicity, with robust immune responses to co-administered vaccines (mRNA, recombinant or ChAdOx1 nCoV-19 COVID-19 vaccines co-administered with a range of seasonal influenza vaccines). Among five studies with non-inferiority objectives, two failed to meet non-inferiority criteria for the anti-SARS-CoV-2 response for some coadministration groups, and one failed to meet criteria for influenza B/Yamagata haemagglutinin inhibition. Two of the other four studies that measured anti-SARS-CoV-2 antibody levels noted lower levels compared to separate administration, although immunogenicity was considered satisfactory. No study assessed co-administration of influenza vaccines with Ad26.COV2.S.

**Added value of this study**

This randomised controlled study evaluated immunological non-inferiority of the co-administration of booster dose of Ad26.COV2.S with seasonal influenza vaccines. The criterion for non-inferiority of the SARS-CoV-2-specific response was met for the standard dose groups, as well as non-inferiority for haemagglutinin inhibition for three out of four influenza vaccine strains—except influenza A/Victoria (H1N1). HI seroprotection and seroconversion rates for A/Victoria (H1N1) exceeded CBER licensure criteria for seasonal influenza vaccines. There was no evidence that co-administration led to clinically important effects on immunogenicity or impacted reactogenicity or safety compared to separate administration.

**Implications of all the available evidence**

This study contributes to our understanding of the interactions between COVID-19 and influenza vaccines, which is pivotal to inform and shape vaccination strategies involving COVID-19 boosters and the prevention of other respiratory infectious diseases in adults.

## Introduction

The co-occurrence of winter outbreaks of coronavirus disease 2019 (COVID-19) and the annual influenza season poses an ongoing problem for over-burdened public health systems worldwide.^1^ Both COVID-19 and influenza manifest most severely in older adults and immunocompromised individuals, and the incidence and severity of both infections can be mitigated through vaccination.^2^^,^^3^ Co-administration of several vaccines at the same medical visit provides benefits such as convenience, improved timeliness of vaccination, improved vaccine coverage, and could be leveraged to address low vaccination rates in high risk populations subgroups.^4^, ^5^, ^6^ While vaccine co-administration is accepted in paediatric practice, it is less common in adults.^4^ Co-administration of influenza and COVID-19 vaccines at the same healthcare visit could improve vaccine uptake, potentially reducing the burden of severe infections and alleviating the seasonal healthcare burden on hospitals and intensive care facilities.

Co-administration of COVID-19 booster doses and seasonal influenza vaccines was recommended by the United States Centers for Disease Control and Prevention during the 2022–2023 Northern hemisphere influenza season.^7^ Current interim guidance provided by the Strategic Advisory Group of Experts (SAGE), states that the co-administration of inactivated seasonal influenza vaccines and any dose of a COVID-19 vaccine is acceptable, given the known risks associated with adults infected with influenza virus or severe acute respiratory syndrome coronavirus 2 (SARS-CoV-2).^8^ Notwithstanding, the World Health Organization urges further research on co-administration of these two types of vaccines in a COVID-19 booster setting, and the generation of additional safety and immunogenicity data following co-administration.^8^

There is a growing body of evidence suggesting that co-administration of a range of different COVID-19 booster doses with seasonal influenza vaccines is immunogenic, with clinically acceptable reactogenicity and safety.^9^, ^10^, ^11^, ^12^, ^13^, ^14^, ^15^, ^16^ However, four out of eight studies either failed to meet non-inferiority criteria for the SARS-CoV-2 antibody response, or reported a lower SARS-CoV-2 antibody response, in groups that received a co-administered COVID-19 booster and an influenza vaccine, although immunogenicity was considered acceptable.^9^, ^10^, ^11^, ^12^, ^13^ There is no evidence to suggest that co-administration of COVID-19 and influenza has an adverse impact on safety or on vaccine efficacy in preventing COVID-19 in a primary or booster setting.^12^^,^^17^

To date there has been one co-administration study assessing immunogenicity and safety of an adenovirus-vectored vaccine with influenza vaccines. Lazarus et al.,^13^ evaluated co-administration of ChAdOx1 nCoV-19 with either cellular quadrivalent, adjuvanted trivalent, or recombinant quadrivalent influenza vaccines compared to their separate administration. Until now, the immunogenicity and safety of Ad26.COV2.S co-administered with a seasonal influenza vaccine has not been reported.

Ad26.COV2.S is composed of a recombinant, replication-incompetent human adenovirus type 26 vector that encodes the SARS-CoV-2 S protein in its prefusion conformation. In clinical trials, one dose of Ad26.COV2.S was effective in preventing moderate-to-severe COVID-19, as well as COVID-19-associated hospitalisations and deaths.^18^, ^19^, ^20^ Ad26.COV2.S was granted conditional marketing authorisation by the European Commission on 11 March 2021 and was authorised or conditionally approved in more than 120 countries/territories globally. We investigated the reactogenicity, safety and immunogenicity of co-administration of Ad26.COV2.S with standard dose (SD) and high dose (HD) influenza vaccines in adults.

## Methods

### Study design

This randomised, double-blind, phase 3 study was conducted at 27 centres in Belgium, Poland, and the United States. We hypothesized that coadministration of Ad26.COV2.S and SD seasonal influenza vaccines would be non-inferior to separate vaccine administration in terms of immunogenicity. The co-primary objectives were to demonstrate the non-inferiority of the humoral immune response of the four influenza vaccine strains, and non-inferiority of the anti-Spike (S) antibody response, 28 days after concomitant administration of Ad26.COV2.S and a seasonal quadrivalent SD influenza vaccine compared to administration of each vaccine alone. The study was originally designed to also demonstrate non-inferiority of co-administration of Ad26.COV2.S and a seasonal quadrivalent HD influenza vaccine in adults aged ≥65 years. In response to limited recruitment due to early COVID-19 vaccination campaigns prioritizing this age-group, humoral immunogenicity of Ad26.COV2.S co-administered with a seasonal quadrivalent HD vaccines in adults aged ≥65 years was amended to become a secondary endpoint. Reactogenicity and safety of the study vaccines were also secondary endpoints.

### Ethics

The study adhered to the principles of the Declaration of Helsinki and to the Good Clinical Practice guidelines and the protocol was approved by all applicable institutional ethics review boards. All participants provided written informed consent prior to enrolment.

### Participants

Eligible participants were healthy or medically stable persons aged at least 18 years who had completed primary vaccination with an authorised/licensed COVID-19 vaccine at least 6 months prior to the study start, or who were COVID-19 vaccine-naïve. Participants who had recovered from COVID-19 were also eligible for enrolment. Exclusion criteria included previous vaccination with a 2021–2022 Northern Hemisphere seasonal influenza vaccine, a history of severe adverse reactions associated with vaccination, severe allergies to any component of the study vaccines, acute severe febrile illness or acute infection, pregnancy, and breastfeeding. Participants were prohibited from receiving any live vaccine within 28 days or any other (non-live) vaccine within 14 days of the first dose of study vaccine. Participants with immune dysfunction, with a history of thrombosis with thrombocytopenia syndrome (TTS) or capillary leak syndrome, were excluded from the study. A full list of inclusion and exclusion criteria are provided in the Appendix (pp 1).

### Randomisation and masking

Participants aged ≥18–64 years were randomised 1:1 to one of two groups based on a computer-generated randomisation schedule prepared before the study according to the sponsor's guidelines. Participants aged ≥65 years were centrally randomised 1:1:1:1 to one of four groups, including the SD groups that contributed to the primary objective. Randomisation was balanced by using randomly permuted blocks and was stratified by age (≥18 to ≤64 years of age versus ≥65 years of age) and previous SARS-CoV-2 vaccination history (*Vaxzevria,* AstraZeneca, *Comirnaty* Pfizer-BioNTech, *SpikeVax* Moderna, Ad26.COV2-S Janssen, or COVID-19 vaccine naïve).

Study vaccines were prepared by an unblinded pharmacist or other qualified study site personnel with primary responsibility for study vaccine preparation and dispensing. Blinding was maintained by the administration of the vaccine in a masked syringe by a blinded study vaccine administrator.

### Procedures

**Fig. 1:**
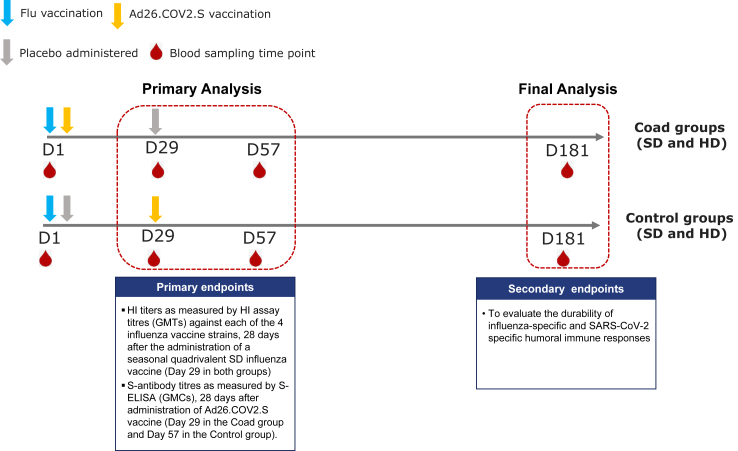
Vaccination schedule and timing of the analysis of primary and secondary endpoints D, study day, GMC/GMT, geometric mean concentration/titre; HI, haemagglutinin inhibition; HD, high dose; SD, standard dose.

There were four study groups (Table 1). Participants aged ≥18–64 years were randomised (1:1) to receive either seasonal quadrivalent SD influenza vaccine (*Afluria* Quadrivalent, Seqirus) administered concomitantly Ad26.COV2.S on Day 1, with placebo (0.9% NaCl) on Day 29 (Coad_SD group, experimental schedule), or the seasonal quadrivalent SD influenza vaccine and placebo on Day 1, and Ad26.COV2.S on Day 29 (Control_SD group, control schedule). Participants aged ≥65 years were randomised (1:1:1:1) to the Coad–SD and Control_SD groups, or to the same schedules using the seasonal quadrivalent HD influenza vaccine (*Fluzone* High-dose Quadrivalent, Sanofi Pasteur Inc) (Coad_HD and Control_HD groups) (Fig. 1).

**Table 1:** Study groups and vaccination days.

Group	Age group	Day 1	Day 29
Coad_SD group	≥18 years	Ad26.COV2.S + Q SD influenza vaccine	Placebo
Control_SD group	≥18 years	Placebo + Q SD influenza vaccine	Ad26.COV2.S
Coad_HD group	≥65 years	Ad26.COV2.S + Q HD influenza vaccine	Placebo
Control_HD group	≥65 years	Placebo + Q HD influenza vaccine	Ad26.COV2.S

The administration of influenza vaccines was aligned with age-based indications for influenza vaccines. Both the quadrivalent SD and HD influenza vaccines were indicated for active immunisation for prevention of disease caused by influenza A subtype viruses and type B viruses contained in the vaccine, in line with WHO recommendations for influenza strains to be used in the Northern Hemisphere 2021–2022 influenza season. The vaccine composition is provided in the Appendix (pp 4).

Ad26.COV2.S was supplied at a concentration of 1 × 10^11^ viral particles/ml in single-use vials, with an extractable volume of 0.5 mL, giving and administered dose of 5 × 10^10^ viral particles/ml. All study vaccines were administered by intramuscular injection into the deltoid muscle. Each participant received two injections administered in opposite arms on Day 1, and a single injection, preferably into the non-dominant arm, on Day 29. After each vaccination, participants remained under observation at the study site for at least 15 min. Blood samples were collected prior to and 28 days after each vaccination, and at 6 months post-vaccination. Vital signs and physical examinations were conducted at scheduled visits. At each visit, participants were asked if they had undergone an off-study COVID-19 diagnostic test. Positive cases were recorded as AEs. Blood samples collected at Day 1 and Day 29 included whole blood for complete blood count including platelet count.

The trial is registered at clinicaltrials.gov NCT05091307.

### Outcomes

Haemagglutinin inhibition (HI) was measured at Vismederi as previously described.^21^ The lower limit of quantification (LLOQ) of the assay was 10. For the co-primary endpoint, HI titres measured at Day 29 were compared between the Control_SD group and the Coad_SD group.

Antibody binding activity was measured at Nexelis by an S-specific enzyme-linked immunosorbent assay (S-ELISA) as described previously (LLOQ = 50.3 EU/ml).^22^ Taking into account the difference in the timing of administration of Ad26.COV2.S in each group, the co-primary endpoint compared anti-S antibodies measured on Day 29 in the Coad-SD group and Day 57 (28-days after vaccination) in the Control-SD group. Secondary immunogenicity endpoints were measured until 6 months after the second vaccination.

Reactogenicity was assessed in all participants using electronic diaries. Solicited local and systemic adverse events (AEs) were recorded for 7 days post-vaccination (or until resolution). Other (unsolicited) AEs were reported until 28 days post vaccination. All serious adverse events (SAEs), medically attended AEs, and AEs leading to discontinuation from the study/vaccination were to be reported until study end. Medically attended AEs included hospital, emergency room, urgent care clinic, or other visits to or from medical personnel for any reason. TTS was considered an AE of special interest (AESI) for safety follow-up. All cases of thrombotic events and/or thrombocytopenia (defined as platelet count below 150,000/μL post-vaccination) were considered a suspected AESI. Cases with thrombosis and thrombocytopenia co-occurring within 42 days qualified for TTS assessment by the TTS Adjudication Committee which held appropriate expertise to determine whether it was a case of TTS.

### Statistical analysis

The sample size assumed a standard deviation (Std Dev) of 0.53 for HI antibody titres against the four influenza vaccine strains, and a SD of 0.50 for anti-S antibodies, at 28 days after vaccination. With 610 eligible participants, a two-sided alpha of 5% and a non-inferiority margin of 1.5, the overall power to show non-inferiority, in terms of HI titres against the four influenza vaccine strains and anti-S antibodies at Day 29, was at least 90%. Additional assumptions are provided in the Appendix pp 5.

The Full Analysis Set included all randomised participants with at least one documented study vaccine administration and was used for the analysis of safety. The Per Protocol Influenza Immunogenicity set included all randomised participants for whom immunogenicity data were available for at least one of the influenza vaccine strains. The Per Protocol SARS-CoV-2 Immunogenicity Set included all randomised participants for whom SARS-CoV-2 immunogenicity data were available. Samples obtained from participants at timepoints after molecularly confirmed natural SARS-CoV-2 infection (viral RNA test result) were excluded from the analysis. The primary objectives were evaluated when all participants had completed the 28-day visit after the second study vaccination or discontinued earlier (Primary analysis). Secondary endpoints were assessed at the Final analysis, performed when all participants had completed the 6-month follow-up period after the second vaccination, or discontinued earlier.

For HI titres against each of the four influenza vaccine strains and for anti-S antibody concentrations, an analysis of variance model was fitted with the respective titres as dependent variable and group (Control or Coad), age category (as stratified), history of COVID-19 vaccination, and baseline anti-S antibody concentration as independent variables. The ANOVA models were used to calculate confidence intervals (CIs) around the difference and were back transformed (by exponentiation) to CIs around a geometric mean concentration/titre (GMC/GMT) ratio. Non-inferiority was demonstrated if the upper limit of the 95% CI on the GMC/GMT ratios were ≤1.5 for all of the co-primary endpoints. Exploratory sub-group analyses were conducted by SARS-CoV-2 serostatus at baseline as determined by N-serology and S-ELISA.

Seroconversion was defined for each of the four influenza vaccine strains as an HI titre ≥1:40 in participants with a pre-vaccination HI titre of <1:10, or a ≥4-fold increase in HI titre in participants with a pre-vaccination HI titre of ≥1:10. Seroprotection was defined for each of the four influenza vaccine strains as a post-vaccination HI titre ≥1:40.

For the calculation of GMTs/GMCs, values below LLOQ were imputed to LLOQ/2, except for the calculation of the geometric mean of the increase from baseline where values below LLOQ were imputed to the LLOQ. Missing immunogenicity data were not imputed.

Descriptive statistics (geometric mean and 95% CI) were calculated for continuous immunogenicity parameters at all time points. Continuous variables were summarised using median, minimum, and maximum. Frequencies and percentages were generated for categorical variables.

The verbatim terms used by investigators to identify AEs were coded using the Medical Dictionary for Regulatory Activities (MedDRA) Version 25.0. All solicited AEs were considered vaccine related. All other AEs were assessed for causality to study vaccination by the investigators.

Statistical analysis was done using SAS Studio 3.8.

### Role of the funding source

Janssen Vaccines & Prevention BV was involved in all aspects of the study design, analysis, and writing of the clinical study report.

## Results

### Study participants

**Fig. 2:**
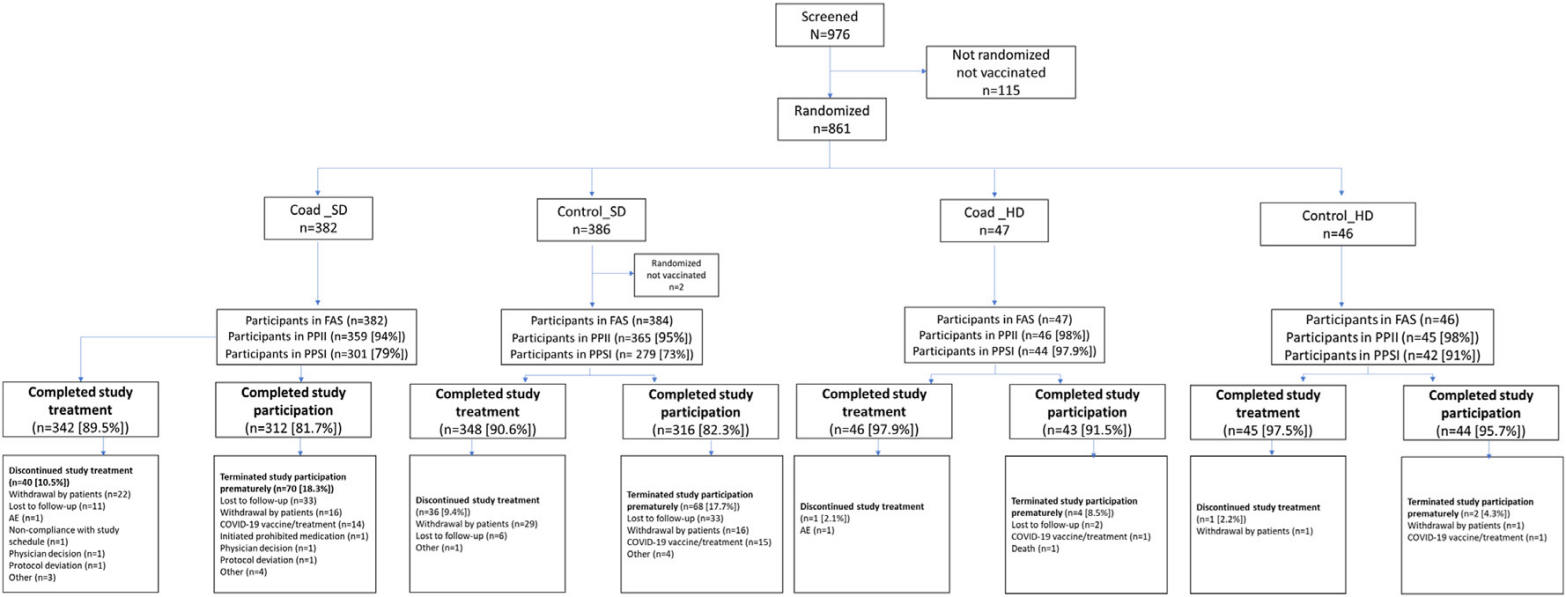
Participant flow (Primary analysis)∗. ∗ when all participants had completed the visit 28 days after the second study vaccination, or discontinued earlier. AE, adverse events; COAD_HD/SD; participants received seasonal quadrivalent high-dose/standard dose influenza vaccine administered concomitantly with Ad26.COV2.S on Day 1, with placebo (0.9% NaCl) on Day 29; Control_HD/SD, participants received seasonal quadrivalent high-dose/standard influenza vaccine and placebo on Day 1, and Ad26.COV2.S on Day 29; FAS, Full analysis set; PPII, Per protocol influenza immunogenicity set; PPSI, Per protocol SARS-CoV-2 immunogenicity set.

The study was conducted between 02 November 2021 and 28 November 2022. Of 976 subjects screened, 859 were randomised and vaccinated, and 781 (91.5%) completed the study treatment at the time of the Primary analysis (Fig. 2). The two most common reasons for discontinuing vaccination were withdrawal of consent by the participant (6%) and loss to follow-up (2%). Two participants were discontinued from the study participation during the treatment phase. One participant in the Coad_SD group reported Grade 2 urticaria considered to be related to vaccination by the investigator, and one participant in the Coad_HD group had a cardiac arrest (SAE) 2 days after dose 1 that resulted in death, considered unrelated to vaccination. This participant was a 69-year-old female with a history of atrial fibrillation, hypertension, type 2 diabetes mellitus, and gastroesophageal reflux disease.

Major protocol deviations were recorded for 318 (37%) participants across all study groups. The majority of protocol deviations (n = 274, 32%) were due to receipt of another COVID-19 vaccine during the study according to evolving local recommendations. These participants were excluded from the immunogenicity analysis for timepoints after the date of the COVID-19 vaccination. There were 61 (16%) participants in the Coad-SD group and 59 (15%) in the Control_SD group who had major protocol deviations with a potential impact on the primary immunogenicity analysis and who were excluded from the analysis of the primary endpoint.

For the primary immunogenicity analysis, the Per Protocol Influenza Immunogenicity set consisted of 815 participants: 359 in the Coad-SD group, 365 in the Control_SD group, 46 in the Coad_HD group and 45 in the Control_HD group. The Per Protocol SARS-CoV-2 Immunogenicity set consisted of 666 participants, 301, 279, 44, and 42 in the respective groups (Fig. 2).

The mean age of participants was 46.2 years (SD 14.92) in the Coad-SD group and 45.5 years (Std Dev 15.25) in the Control_SD group, and 88.0% and 87.8% respectively were aged between 18 and 64 years. Most participants were white (81.4% and 80.7%, respectively).

The mean age of participants was 71.1 years (SD 4.58) in the Coad-HD group and 71.3 years (Std Dev 5.29) in the Control_HD group. Most participants were white (97.9% and 97.8%, respectively).

Demographic characteristics and prior COVID-19 vaccination experience were well balanced between the two SD and the two HD groups (Table 2). In total, 386 (56%) participants reported previous COVID-19 infection.

**Table 2:** Summary of demographic and baseline characteristics (Full Analysis Set, Final Analysis).

	Coad_SD N = 382	Control_SD N = 384	Coad_HD N = 47	Control_HD N = 46
Age (years)				
Mean (SD)	46.2 (14.92)	45.5 (15.25)	71.1 (4.58)	71.3 (5.29)
Median (range)	48.0 (18; 80)	45.0 (18; 81)	71.0 (65; 82)	70.0 (65; 87)
18–64 n (%)	336 (88.0)	337 (87.8)	0	0
≥65 n (%)	46 (12.0)	47 (12.2)	47 (100)	46 (100)
Sex n (%)				
Female	191 (50.0)	177 (46.1)	22 (46.8)	33 (71.7)
Male	191 (50.0)	207 (53.9)	25 (53.2)	13 (28.3)
Race n (%)				
American Indian/Alaska Native	1 (0.3)	3 (0.8)	0	0
Asian	7 (1.8)	10 (2.6)	1 (2.1)	1 (2.2)
Black/African American	48 (12.6)	52 (13.5)	0	0
Native Hawaiian/other Pacific Islander	0	1 (0.3)	0	0
White	311 (81.4)	310 (80.7)	46 (97.9)	45 (97.8)
Multiple/unknown/not reported	15 (3.9)	8 (2.0)	0	0
Ethnicity n (%)				
Hispanic or Latino	117 (30.6)	109 (28.4)	4 (8.5)	4 (8.7)
Not Hispanic or Latino	262 (68.6)	274 (71.4)	43 (91.5)	42 (91.3)
Unknown/not reported	3 (0.8)	1 (0.3)	0	0
COVID-19 vaccination history n (%)^a^				
Ad26.COV2.S Janssen, adenovirus vectored	33 (8.6)	35 (9.1)	2 (4.3)	2 (4.3)
*Comirnaty* Pfizer-BioNTech, mRNA	181 (47.4)	175 (45.6)	28 (59.6)	29 (63.0)
*SpikeVax* Moderna, mRNA	84 (22.0)	84 (21.9)	11 (23.4)	11 (23.9)
*Vaxzevria* ChAdOx1 nCoV-19, AstraZeneca	17 (4.5)	18 (4.7)	4 (8.5)	2 (4.3)
Naive	67 (17.5)	72 (18.8)	2 (4.3)	2 (4.3)

N, number of participants; SD, standard deviation.

a Participants may have received more than one type of COVID-19 vaccine previously.

### Immunogenicity

#### Primary objective

**Fig. 3:**
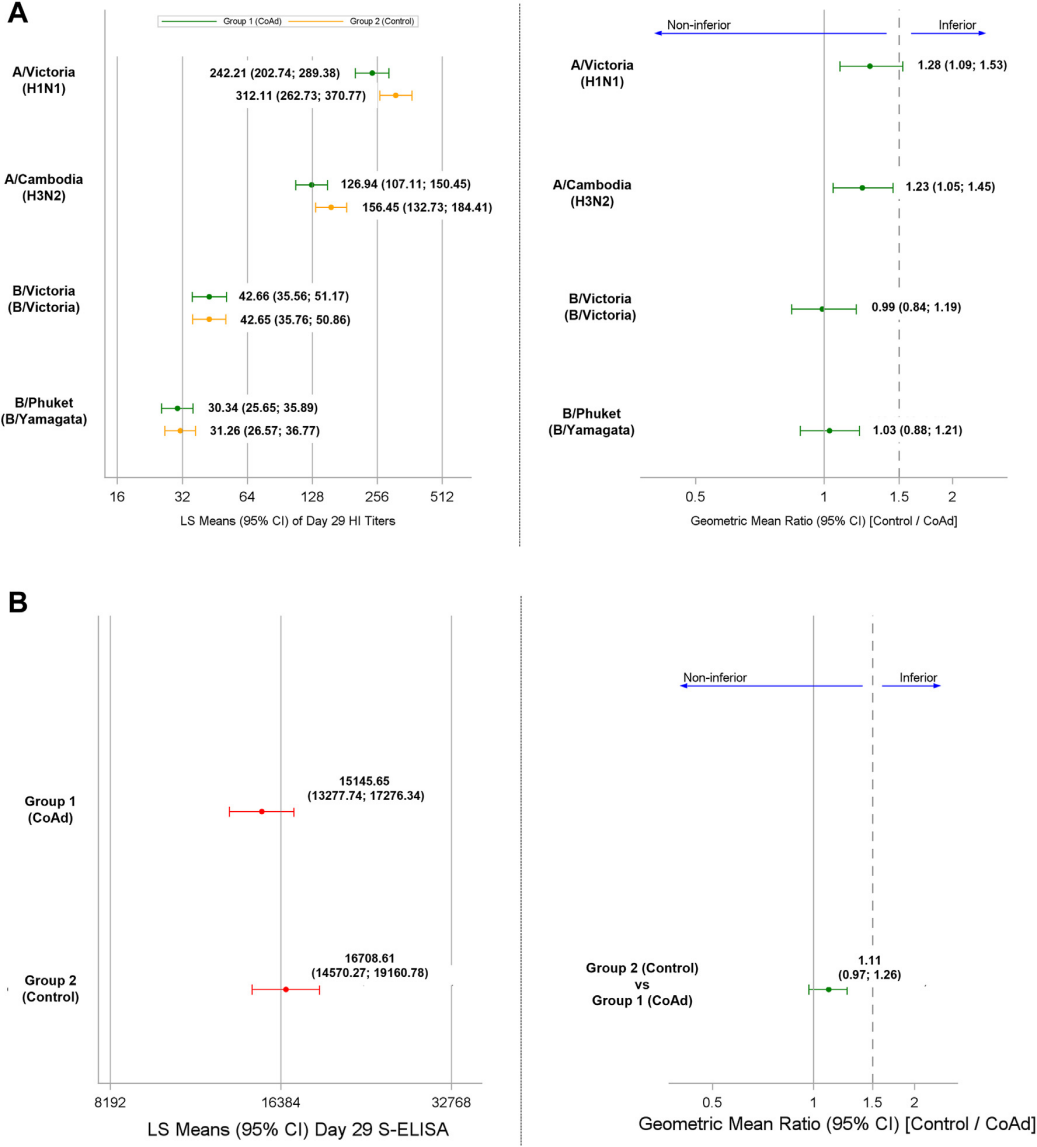
Non-inferiority assessment: haemagglutinin inhibition responses against A) each of the four influenza vaccine strains and B) anti-S antibodies against SARS-CoV-2 (Primary objective). An analysis of variance (ANOVA) model was fitted (using SAS proc mixed) per outcome with the respective Day 29 titres (log_10_-transformed) as dependent variable, and group (Control or CoAd), age category (as stratified), previous SARS-CoV-2 vaccination, and baseline S-ELISA antibody titres as independent variables.

The pre-specified non-inferiority criteria for the SD groups were met for the antibody responses against SARS-CoV-2 and for three out of four influenza vaccine strains when both vaccines were co-administered versus either vaccine administered alone because the upper limit of the 95% CI on the GMC/GMT ratios were ≤1.5 (Fig. 3). The study did not demonstrate non-inferiority for A/Victoria H1N1 because the upper limit of 95% CI on the GMT ratio was above 1.5 (ratio 1.28, 95% CI 1.09–1.53).

#### Secondary objectives–SD groups

**Fig. 4:**
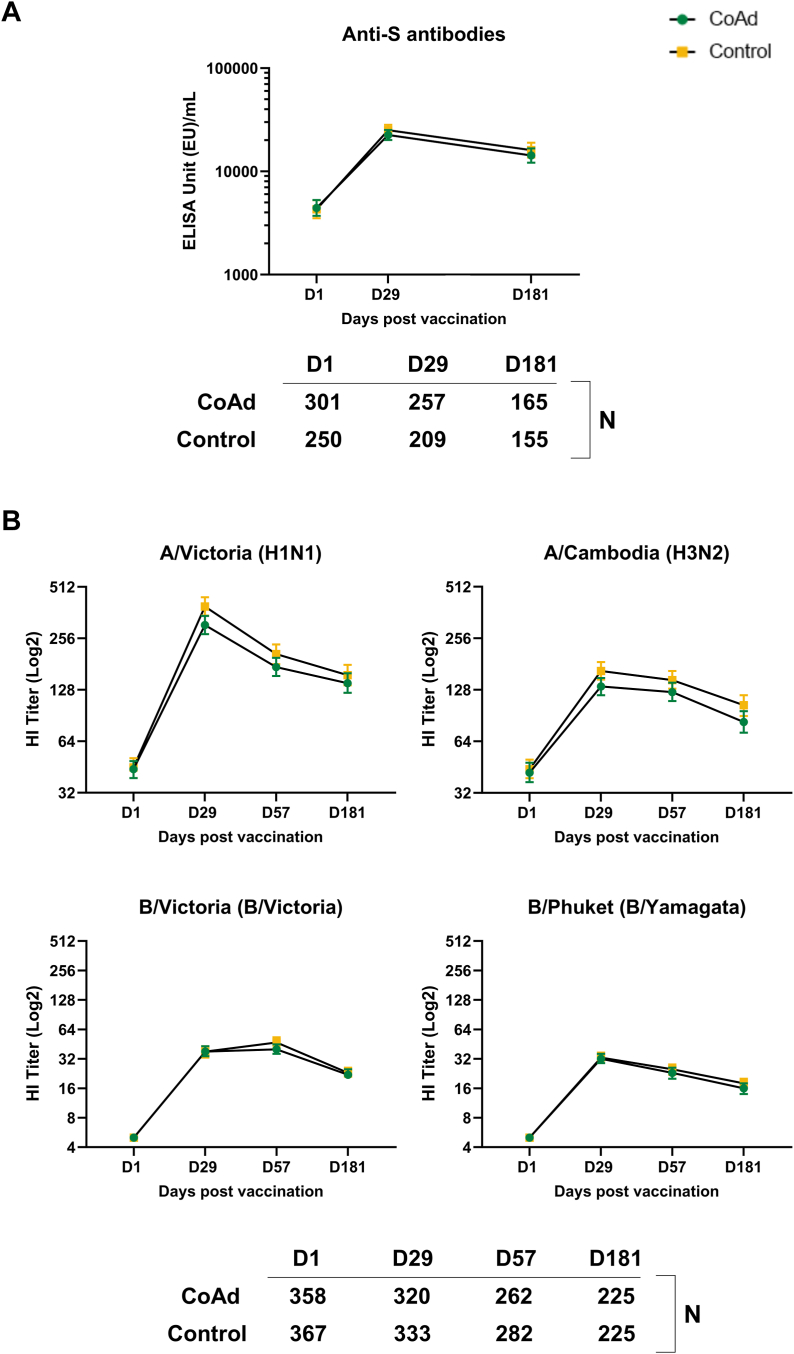
Immunogenicity until 6 months after dose 2 in participants who received *standard dose* influenza vaccine with or without co-administered Ad26.COV2.S A) anti-S antibodies (Per Protocol SARS-CoV-2 Immunogenicity Set), B) haemagglutinin inhibition for vaccine influenza strains (Per Protocol Influenza Immunogenicity Set). D, day; HI, haemagglutinin inhibition; N, number of participants with data at each time point.

Concomitant administration of Ad26.COV2.S with quadrivalent SD seasonal influenza vaccine induced anti-S antibody responses that were similar compared to administration of Ad26.COV2.S alone (Fig. 4). At 28 days after vaccination with Ad26.COV2.S, the geometric mean increase from baseline was 5.3-fold in the Coad_SD group to 22,531 (95% CI 20,140–25,205), and 5.7-fold in the Control_SD group to 25,035 (95% CI 22,189–28,246) (Table S1). By Day 181, anti-S antibody concentrations had declined similarly in both groups but remained well above baseline levels (Table S1).

**Fig. 5:**
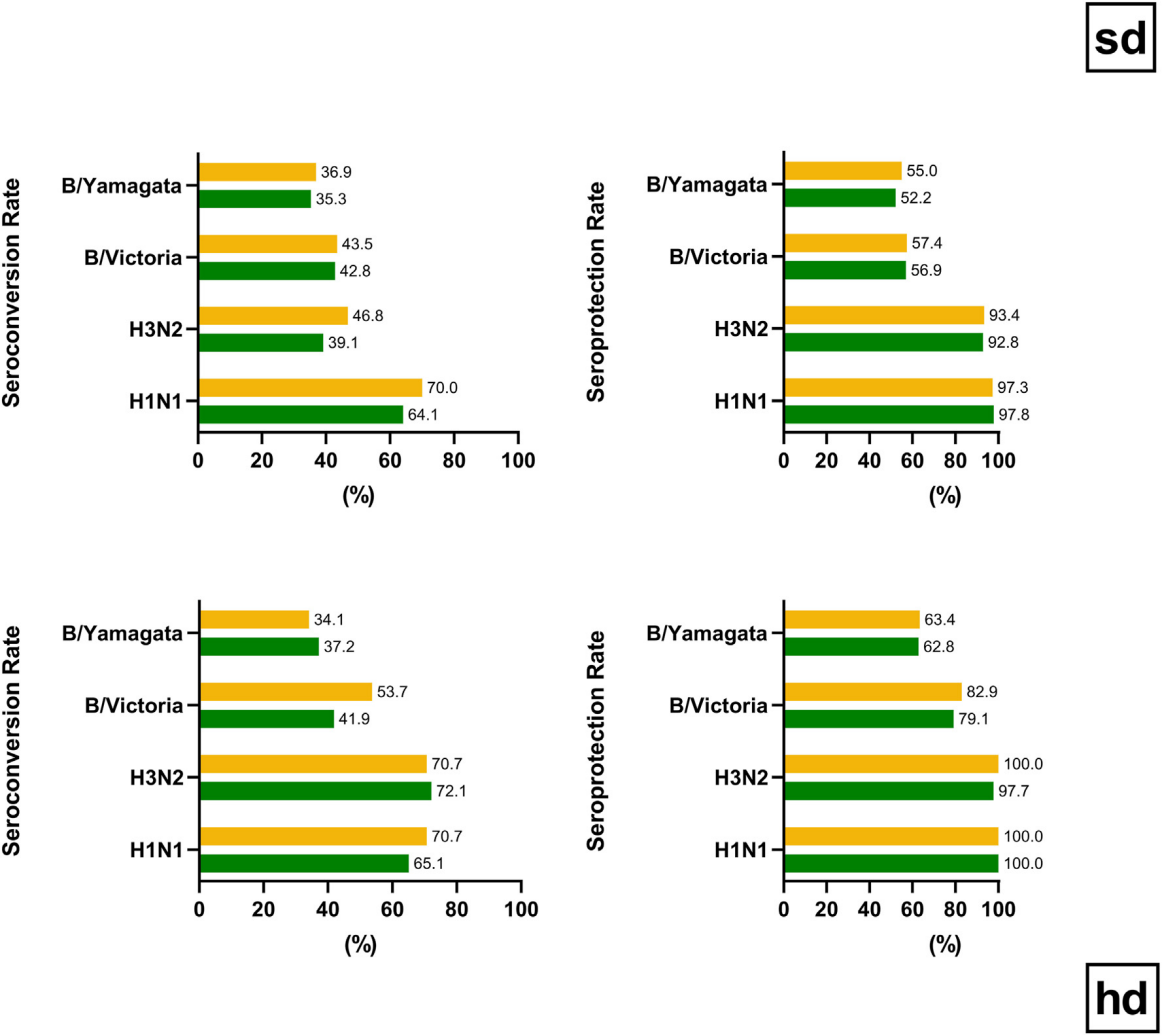
Haemagglutinin inhibition (HI) seroconversion and seroprotection rates at Day 29 in *standard dose* (sd) and *high dose* (hd) influenza vaccine groups with or without co-administered Ad26.COV2.S (Per Protocol Influenza Immunogenicity Set). Seroconversion was defined for each of the four influenza vaccine strains as an HI titre ≥1:40 in participants with a pre-vaccination HI titre of <1:10, or a ≥4-fold increase in HI titre in participants with a pre-vaccination HI titre of ≥1:10. Seroprotection was defined for each of the four influenza vaccine strains as a post-vaccination HI titre ≥1:40. Green refers to Coad groups. Orange refers to Control groups. Data are tabulated in Tables S2 and S4.

Robust immune responses against all four influenza vaccine strains were observed 28 days after vaccination with quadrivalent SD seasonal influenza vaccine with or without concomitant Ad26.COV2.S (Fig. 4). At 28 days post-vaccination, seroprotection rates in both SD study groups ranged between 92.8% to 97.8% for influenza/A strains, and from 52.2% to 57.4% for influenza/B strains (Fig. 5). Seroconversion rates ranged from 39.1% to 70.0% for influenza/A strains and 35.3% to 43.5% for influenza/B strains. The Day 29 HI GMT for A/Victoria (H1N1) was 306 (95% CI 271–346) in the Coad-SD group and 393 (95% CI 348–445) in the Control_SD group. For A/Cambodia (H3N2) the GMTs were 134 (95% CI 119–150) and 165 (95% CI 147–186), respectively, for B/Victoria (B/Victoria), 38 (95% CI 34–43) and 38 (95% CI 33–43), and for B/Phuket (B/Yamagata), 32 (95% CI 29–36) and 33 (95% CI 30–37), respectively (Fig. 4). By Day 181 (6 months after vaccination) HI GMTs of all four influenza strains had undergone similar declines in the Coad_SD and Control_SD groups. Seroprotection rates at Day 181 ranged between 82.7% to 94.2% for influenza/A strains and from 25.8% to 38.7% for influenza/B strains. Descriptive analyses suggested comparable responses in the Coad_SD and Control_SD groups at each timepoint (Table S2).

Descriptive analysis by baseline serostatus showed no differences between the Coad_SD and Control_SD groups irrespective of serostatus (Figs. S1 and S2).

#### Secondary objectives–HD groups

**Fig. 6:**
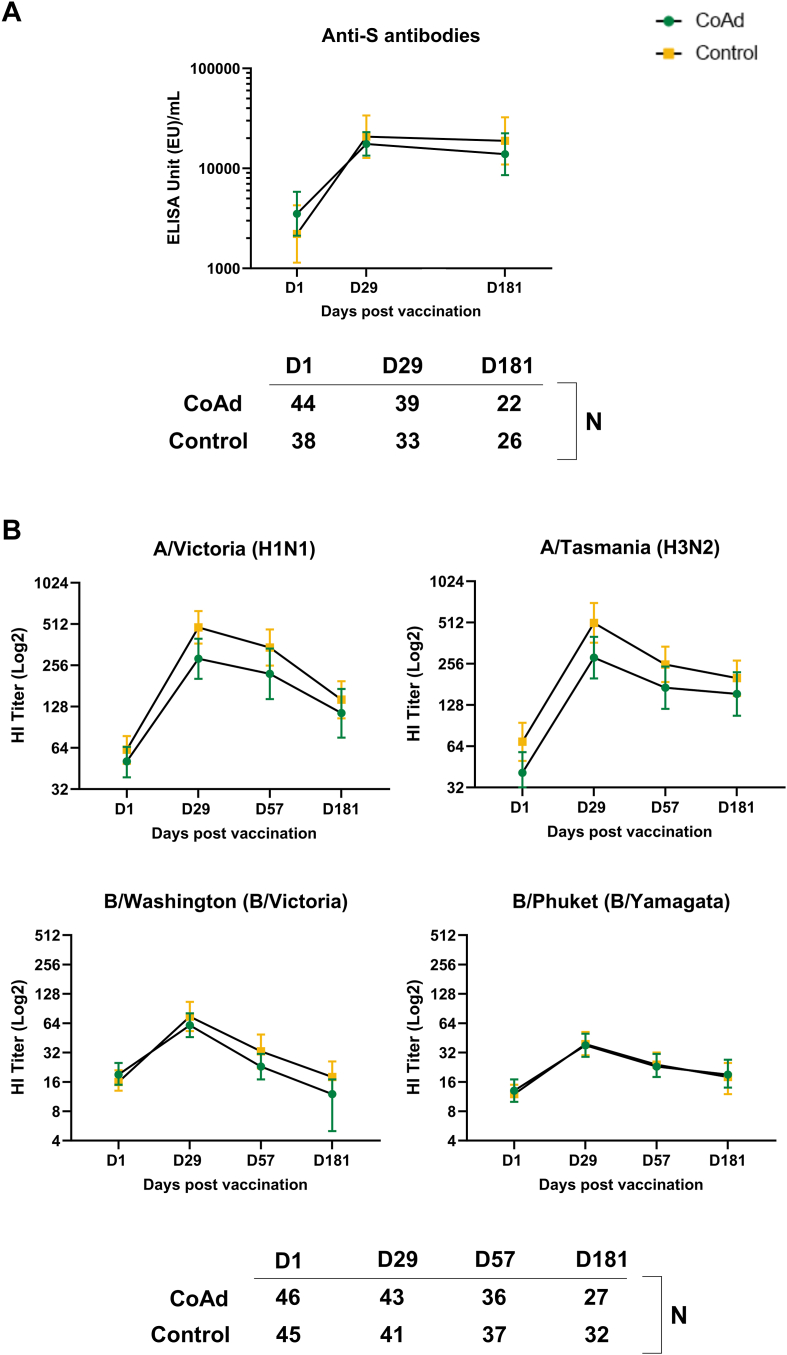
Immunogenicity until 6 months after dose 2 in participants who received *high dose* influenza vaccine with or without co-administered Ad26.COV2.S A) anti-S antibodies (Per Protocol SARS-CoV-2 Immunogenicity Set), B) haemagglutinin inhibition for vaccine influenza strains (Per Protocol Influenza Immunogenicity Set). D, day; HI, haemagglutinin inhibition; N, number of participants with data at each time point.

Concomitant administration of Ad26.COV2.S with quadrivalent HD seasonal influenza vaccine induced S-binding antibody responses that were similar to administration of Ad26.COV2.S alone (Fig. 6). At 28 days after vaccination with Ad26.COV2.S, the geometric mean increase from baseline was 4.3-fold in the Coad_HD group to 17,569 (95% CI 13,391–23,051) and 8.3-fold in the Control_HD group to 20,743 (95% CI 12,732–33,794). By Day 181, anti-S antibody concentrations had decline similarly in both groups but remined well above baseline levels.

Robust immune responses to all four influenza vaccine strains were observed 28 days after vaccination with quadrivalent HD seasonal influenza vaccine with or without concomitant Ad26.COV2.S (Fig. 6). At 28 days post-vaccination, seroprotection rates in both HD study groups ranged between 97.7%–100% for influenza/A strains, and from 62.8% to 82.9% for influenza/B strains, and seroconversion rates ranged from 65.1% to 72.1% and 34.1% to 53.7%, respectively. The Day 29 HI GMT for A/Victoria (H1N1) was 286 (95% CI 204–400) in the Coad-HD group and 484 (95% CI 369–636) in the Control_HD group. For A/Tasmania (H3N2) the GMTs were 284 (95% CI 200–402) and 509 (95% CI 365–711), respectively, for B/Washington (B/Victoria), 61 (95% CI 46–81) and 75 (95% CI 53–106), and for B/Phuket (B/Yamagata) 38 (95% CI 29–50) and 39 (95% CI 30–52), respectively. By Day 181 (6 months after vaccination) HI GMTs had declined similarly against all four influenza strains in both groups. Seroprotection rates at Day 181 ranged between 88.9% and 100% for influenza/A strains and from 11.1% to 33.3% for influenza/B strains (Fig. 5). Secondary endpoint analyses suggested comparable responses in the Coad_HD and Control_HD groups at each timepoint (Table S4).

Descriptive subgroup analysis by serostatus at baseline showed no differences between the Coad_HD and Control_HD groups irrespective of serostatus (Figs. S3 and S4), although more variation was observed versus the SD groups.

### Reactogenicity and safety

The incidence and intensity of solicited injection site and systemic AEs tended to be higher when seasonal influenza vaccine (SD or HD) was co-administered with Ad26.COV2.S than when administered alone. However, the reactogenicity profile of the Ad26.COV2.S dose administered to the Control groups at dose 2 was similar to co-administered influenza and Ad26.COV2.S vaccines at dose 1 in the Coad groups. Furthermore, when AEs reported after dose 1 and dose 2 were combined, the overall incidence of solicited injection site symptoms was very similar in both groups (Table S5).

**Fig. 7:**
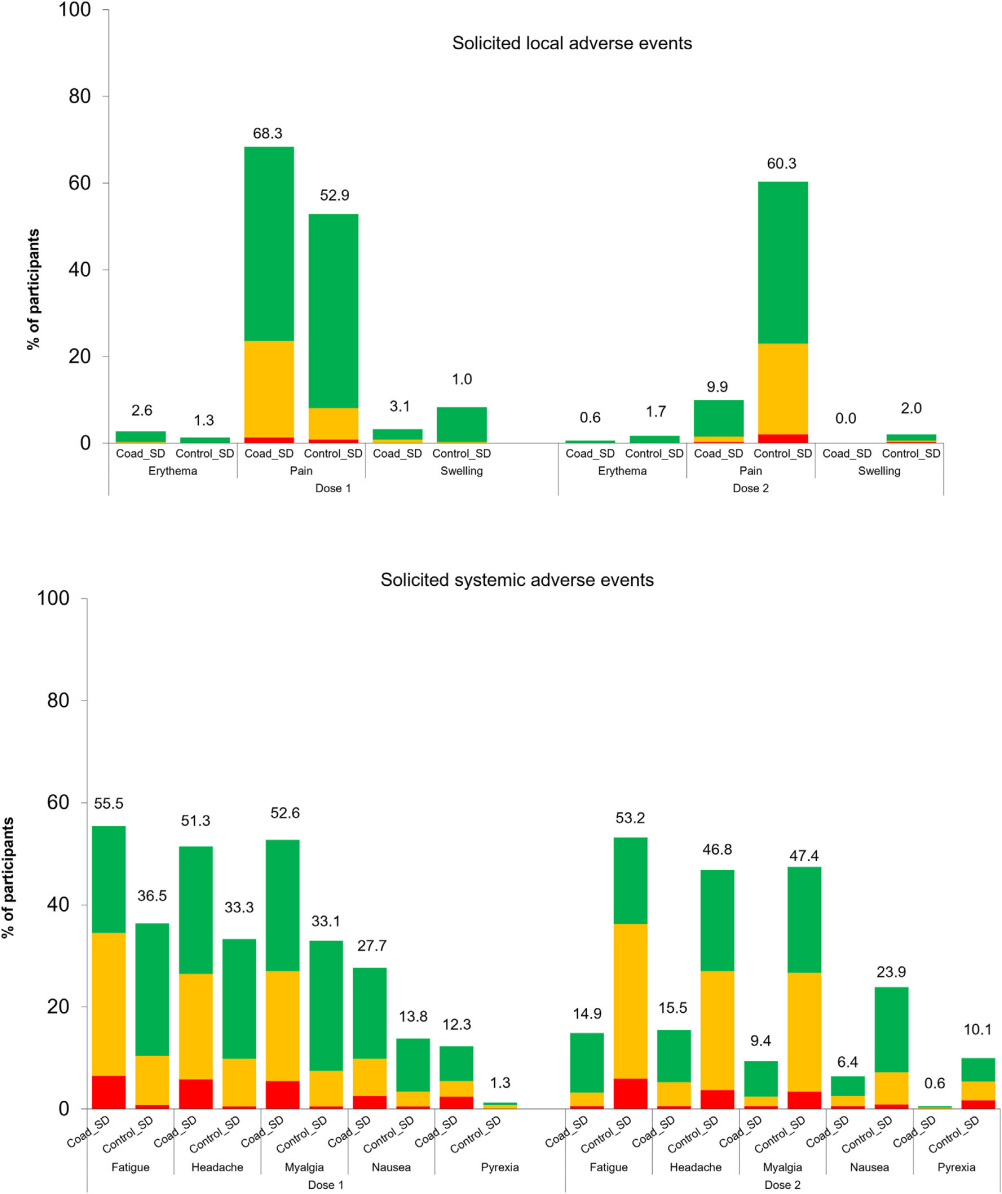
Solicited local (any injection site) and systemic adverse events reported within 7 days after vaccination in the SD groups. Coad-SD group received quadrivalent standard dose influenza vaccine and Ad26.COV2.S at Dose 1 and placebo at Dose 2. Control-SD group received quadrivalent standard dose influenza vaccine and placebo at Dose 1 and Ad26.COV2.S at Dose 2. Green = Grade 1 (mild), orange = Grade 2 (moderate), red = Grade 3 (severe). Pain: Grade 1 = Aware of symptoms but easily tolerated, does not interfere with activity, discomfort only to touch; Grade 2 = Notable symptoms, requires modification in activity or use of medications, discomfort with movement; Grade 3 = Incapacitating symptoms, inability to do work, school, or usual activities, use of narcotic pain reliever. Erythema and swelling: Grade 1 = 25–50 mm; Grad 3 = 51–100 mm; Grade 3 ≥ 100 mm. Nausea: Grade 1 = Minimal symptoms, causes minimal or no interference with work, school, or selfcare activities; Grade 2 = Notable symptoms, requires modification in activity or use of medications, does not result in loss of work, school, or cancellation of social activities; Grade 3 = Incapacitating symptoms, requires bed rest and/or results in loss of work, school, or cancellation of social activities. Fever: Grade 1 = 38.0–38.4 °C; Grade 2 = 38.5–38.9 °C; Grade 3 = 39.0–40.0 °C; Grade 4 ≥ 40.0 °C. Other symptoms: Grade 1 = Minimal symptoms causing no or minimal interference with usual social and functional activities; Grade 2 = Notable symptoms causing greater than minimal interference with usual social and functional activities (may require use of medications); Grade 3 = Severe symptoms causing inability to perform usual social and functional activities and requires medical intervention (may require use of narcotic pain reliever); Grade 4: Hospitalization, inability to perform basic self-care functions.

**Fig. 8:**
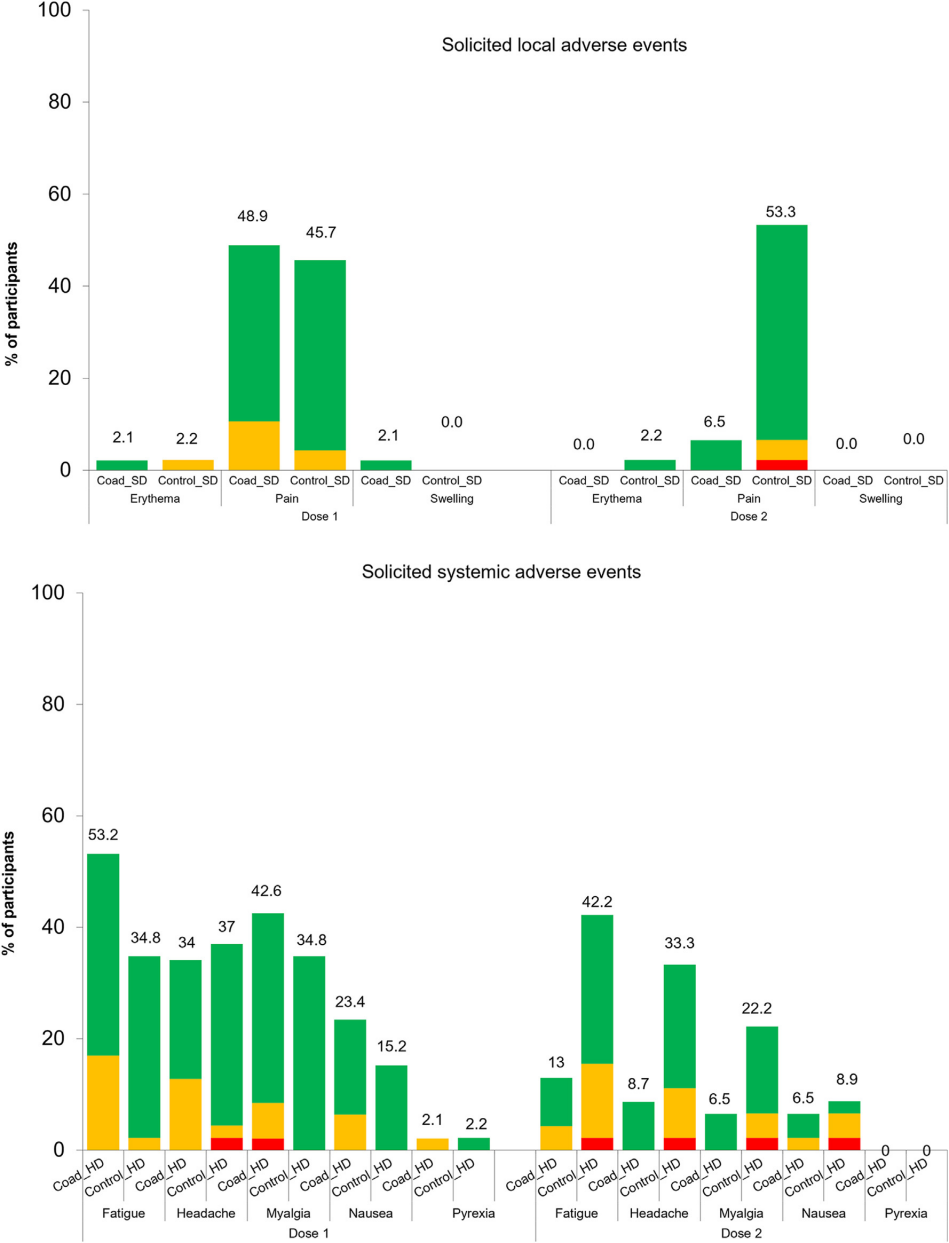
Solicited adverse events reported within 7 days after vaccination in the HD groups. Coad_HD group received quadrivalent high dose influenza vaccine and Ad26.COV2.S at Dose 1 and placebo at Dose 2. Control_HD group received quadrivalent high dose influenza vaccine and placebo at Dose 1 and Ad26.COV2.S at Dose 2. Green = Grade 1 (mild), orange = Grade 2 (moderate), red = Grade 3 (severe). See footnote to Fig. 7 for definitions of Grade 1, 2 and 3 severity.

Pain was the most frequently reported solicited injection site AE. After dose 1, injection site pain was reported by 68.3% of participants in the Coad_SD group, 52.9% in the Control_SD group, 48.9% in the Coad_HD group and 45.7% in the Control_HD group (Fig. 7, Fig. 8). After dose 2 (Ad26.COV2.S in Control groups), the percentage was 60.3% in the Control_SD group and 53.3% in the Control_HD group.

The most frequently reported systemic AEs were fatigue, headache, and myalgia in all groups, reported by 51.3% to 55.5% of participants in the Coad_SD group, 33.1% to 36.5% in the Control_SD group, 34.0% to 53.2% in the Coad_HD group, and 34.0% to 37.0% in the Control_HD group after dose 1. After dose 2 (Ad26.COV2.S in Control groups), the percentages were 46.8% to 53.2% in the Control_SD group and 22.2% to 42.2% in the Control_HD group. Most AEs had a duration of 1–2 days. Grade 3 AEs were uncommon in all study groups (Figs. 7 and 8).

Medically-attended AEs were reported for 13.4% of participants in the Coad_SD group, 13.3% in the Control_SD group, 23.4% in the Coad_HD group, and 34.8% in the Control_HD group. None of the medically attended AEs led to permanent discontinuation of vaccination.

Suspected AESIs (thrombotic events and/or thrombocytopenia) were reported by the investigator in 2.1% (8/382) of participants in the Coad_SD group and 3.1% (12/384) in the Control_SD group and included the following verbatim terms; deep vein thrombosis, pulmonary embolism, heparin-induced thrombocytopenia (positive anti-platelet factor 4 test result encoded by MedDRA as heparin-induced thrombocytopenia), pulmonary embolism, thrombocytopenia, and platelet count decreased. None of the participants had thrombosis and thrombocytopenia that occurred concomitantly; therefore, none of the suspected AESIs qualified for TTS assessment and no case of TTS was reported in this study.

SAEs were reported for nine participants in the Coad_SD group, seven in the Control_SD group, one in the Coad_HD group, and two in the Control_HD group. Three SAEs were considered related to study vaccine by the investigator; one 63-year-old male participant in the Coad_SD group with a history of overweight and past smoking developed a deep vein thrombosis 2 months after dose l. The AE was ongoing at study end and was considered not related to study vaccine by the study sponsor. A 57-year-old male in the Coad_SD group with no relevant history developed asymptomatic thrombocytopenia (platelet count 106,000/μL) 35 days after dose 1 which returned to the normal range 1 month later. A 61-year old male in the Contol_SD group with a history of obesity developed asymptomatic thrombocytopenia (Grade 1, platelet count not specified) 29 days after dose 1, on the day of the second vaccination with Ad26.COV2.S. The event had resolved 1 month later.

## Discussion

This study evaluated the immunological non-inferiority of co-administering Ad26.COV2.S with seasonal influenza vaccines. The anti-S antibody response was non-inferior when Ad26.COV2.S was co-administered with seasonal SD influenza vaccines compared to their sequential administration. Non-inferiority could not be concluded for HI as the pre-specified criterion was not met for one out of the four influenza vaccine strains (A/Victoria H1N1). Nevertheless, 98% of participants in the Coad_SD group and 97% in the Control_SD group were seroprotected after vaccination for A/Victoria (H1N1), and 64.3% and 70.0%, respectively, seroconverted. The responses to A/Victoria H1N1 as well as the three other influenza vaccine strains in the Coad_SD (and Coad-HD) group well exceeded immunological criteria for licensure of seasonal influenza vaccines as required by the US Center for Biologics Evaluation and Research.^23^ To our knowledge only three other studies have evaluated the immunological non-inferiority of concomitant administration of COVID-19 vaccines and influenza vaccines.^14^^,^^15^^,^^24^ In two studies, non-inferiority of the HI response was demonstrated for all four influenza strains when an mRNA COVID-19 booster dose was co-administered with inactivated influenza vaccine in mRNA-primed adults.^14^^,^^24^ Non-inferiority was demonstrated for three out of four influenza strains (except B/Yamagata) when primary vaccination with inactivated COVID-19 vaccine was co-administered with inactivated influenza virus in SARS-CoV-2-naïve adults aged ≥18 years.^15^ Immune interference due to co-administration cannot be excluded based on these data. Differences between studies in the age-groups studied, the study vaccines employed, and SARS-CoV-2 infection status, and COVID-19-vaccination history, and influenza seasonality, among others, may have contributed to different results observed.

Six months after vaccination, immunity waned in all study groups but seroprotection and seroconversion rates remained similar between the Coad and Control groups, indicating that co-administration did not impact the durability of the immune response.

Analysis of the groups that received HD influenza vaccine was descriptive and limited by a small sample size. Concomitant administration of Ad26.COV2.S and the HD influenza vaccine induced lower H1N1 and H3N2 HI titres and similar B/Victoria and B/Yamagata HI titres 28 days after vaccination compared to administration of the HD influenza vaccine alone in adults aged ≥65 years. Anti-S antibodies were similar in the Coad-HD and Control_HD groups.

The incidence and intensity of solicited injection site and systemic AEs tended to be higher at dose 1 when influenza vaccine was co-administered with Ad26.COV2.S. However, the reactogenicity profile following co-administration was similar to a single dose of Ad26.COV2.S given alone. There was no evidence that vaccine co-administration led to an increase in overall AE reports.

Study limitations include the relatively lower number of participants aged ≥65-years who received HD influenza vaccine. Recruitment of larger numbers was impeded by a delay in availability of the influenza vaccine for the study and the issue of recommendations for COVID-19 vaccination of this age-group by authorities; therefore, hypothesis testing in the ≥65-year age group could not be performed. T-cell responses and neutralising antibodies may be important indicators of immunity against SARS-CoV-2 but were not measured in our study.^25^ Finally, longer follow-up of immunogenicity and safety could have provided further insights into vaccine-induced antibody persistence and the occurrence of late or rare AEs in the context of co-administration.

In conclusion, co-administration of Ad26.COV2.S with seasonal SD and HD influenza vaccines induced marked increases in anti-S antibodies, and HI titres considered to indicate protection against all four influenza strains in a majority of participants. There was no evidence that co-administration negatively impacted the safety profile of either vaccine. The study shows that co-administration induced strong immune responses to all vaccine antigens, and support co-administration of Ad26.COV2.S with seasonal influenza vaccine. These data contribute to expanding the body of studies reporting co-administration of seasonal influenza vaccines with COVID-19 vaccines. More broadly, the results could contribute to the body of knowledge around the use of other adenovirus-vector vaccines co-administered with routinely recommended vaccines.

## Contributors

Gabriela Tapia-Calle: Accessed and verified the data, methodology, visualisation, writing—original draft.

Gloria Aguilar: Accessed and verified the data, conceptualization, methodology, writing–review & editing.

Nathalie Vaissiere: Data curation, software, formal analysis, visualisation.

Carla Truyers: Data curation, Software, Formal analysis.

Pedro Ylisastigui: Investigation, resources.

Erik Buntinx: Investigation, resources, writing–review & editing.

Mathieu Le Gars: Conceptualization, supervision, writing–review and editing.

Frank Struyf: Conceptualization, writing–review and editing.

Gert Scheper: Project administration, writing–review & editing.

Macaya Douoguih: Project administration, supervision, writing–review & editing.

Javier Ruiz-Guiñazú: Conceptualization, methodology, supervision.

## Data sharing statement

Janssen has an agreement with the Yale Open Data Access (YODA) Project to serve as the independent review panel for the evaluation of requests for clinical study reports and participant-level data from investigators and physicians for scientific research that will advance medical knowledge and public health. Data will be made available following publication and approval by YODA of any formal requests with a defined analysis plan. For more information on this process or to make a request, please visit the YODA Project site at http://yoda.yale.edu. The data sharing policy of Janssen Pharmaceutical Companies of Johnson & Johnson is available at https://www.janssen.com/clinical-trials/transparency.

## Declaration of interests

Gloria Aguilar is an employee of Johnson & Johnson and holds stock/stock options in Johnson & Johnson, LLC.

Nathalie Vaissiere was an employee of Johnson & Johnson at the time of this study.

Carla Truyers is an employee of Johnson & Johnson.

Mathieu Le Gars was an employee of Johnson & Johnson at the time of this study.

Frank Struyf was an employee of Johnson & Johnson at the time of this study and holds stock/stock options in Johnson & Johnson, LLC. He holds shares in GSK as renumeration for past employment.

Gert Scheper is an employee of Johnson & Johnson and holds stock/stock options in Johnson & Johnson, LLC.

Macaya Douoguih was an employee of Johnson & Johnson at the time of this study and holds stock/stock options in Johnson & Johnson, LLC.

Javier Ruiz-Guiñazú is an employee of Johnson & Johnson and holds stock/stock options in Johnson & Johnson, LLC. He holds shares in GSK as renumeration for past employment.

Pedro Ylisastigui, Gabriela Tapia-Calle, and Erik Buntinx report nothing to declare.

## Appendix A Supplementary data

Appendix and Tables

Redacted_Protocol-Amend
