# Supplementary material for: Safety, reactogenicity, and immunogenicity of Ad26.COV2.S co-administered with a quadrivalent standard-dose or high-dose seasonal influenza vaccine: a non-inferiority randomised controlled trial
Source: eClinicalMedicine. 2025 Jan 7;79:103016. doi: 10.1016/j.eclinm.2024.103016 (PMC11764035; doi:10.1016/j.eclinm.2024.103016)

**Safety, reactogenicity, and immunogenicity of Ad26.COV2.S co-administered with a quadrivalent standard-dose or high-dose seasonal influenza vaccine: a randomised controlled trial**

**Appendix**

**The COV3005 study group**

Robert Patrizi (rpatrizi@its.jnj.com)

Wai Ling (wmo5@its.jnj.com)

Sanne de Ridder (sderidde@its.jnj.com)

Marit de Groot (mgroot@its.jnj.com)

Maria Grazia Pau (mpau@its.jnj.com)

Gerald Weidinger (gweiding@its.jnj.com)

Srividya Pradeep (spradee2@its.jnj.com)

Nadine Salisch ([nsalisch@gmail.com](mailto:nsalisch@gmail.com))

Sophie Cambre (scambre3@its.jnj.com)

**Inclusion Criteria**

Each potential participant must satisfy all of the following criteria to be enrolled in the study:

1. Participant must sign an informed consent form (ICF) indicating that he or she understands the purpose, procedures, and potential risks and benefits of the study, and is willing to participate in the study.

2. Participant is willing and able to adhere to the prohibitions and restrictions specified in this protocol.

3. Participant is male or female aged ≥18 years of age, on the day of signing the ICF.

a. Groups 3 and 4 only: Participant is male or female aged ≥65 years of age on the day of signing the ICF.

4. Participant must be healthy, in the investigator’s clinical judgment, as confirmed by medical history, physical examination, and vital signs performed at screening. Participants may have underlying illnesses, as long as the symptoms and signs are medically controlled.

5. Participant either received complete primary vaccination with an authorized/licensed COVID-19 vaccine (completed ≥6 months prior to the last vaccination received against COVID-19) or is COVID19 -vaccine-naïve.

Note: The following authorized/licensed COVID-19 vaccines are permitted in this study: Vaxzevria (AstraZeneca), Comirnaty (Pfizer-BioNTech), SpikeVax (Moderna), and Ad26.COV2-S (Janssen).

6 In the investigator’s clinical judgment, the participant may have a stable and well-controlled medical condition including comorbidities associated with an increased risk of progression to severe COVID-19 (including stable/well-controlled HIV infection)*. If participants are on medication for a medical condition (including comorbidities associated with an increased risk of progression to severe COVID-19), the medication dose cannot have been modified within 4 weeks preceding vaccination. Participants will be included on the basis of relevant medical history at the investigator’s discretion.

* Stable/well-controlled HIV infection includes:

a. Documented CD4 cell count ≥300 cells/µL within 6 months prior to screening.

b. Documented HIV viral load <50 copies/mL within 6 months prior to screening.

c. Participant must be on a stable anti-retroviral treatment (ART) for 6 months (unless the change is due to tolerability, in which case the regimen can be for only the previous 3 months; changes in the formulation are allowed; nationwide guidelines that require transition from one ART regimen to another are allowed) and the participant must be willing to continue his/her ART throughout the study as directed by his/her local physician.

Note: Participants with ongoing and progressive comorbidities associated with HIV infection will be excluded but comorbidities associated with HIV infection that have been clinically stable for the past 6 months are not an exclusion criterion. Laboratory methods for confirming a diagnosis of HIV infection are: Any evidence (historic or current) from medical records, such as ELISA with confirmation with Western Blot or real-time reverse-transcriptase polymerase chain reaction (RT-PCR), or of a detectable viral load (country-specific regulatory approved tests).

If a potential participant does not have HIV viral load and CD4 cell count data in his/her medical records from the last 6 months, they will be instructed to go to their local health care provider and obtain the necessary data for potential entry into the study. A laboratory result within 6 months of screening does not need to be repeated.

7. Contraceptive (birth control) use by participants should be consistent with local regulations regarding the acceptable methods of contraception for those participating in clinical studies. Before randomization, participants who were born female must be either -

a. Not of childbearing potential

b. Of childbearing potential and practicing a highly effective method of contraception and agrees to remain on such a method of contraception from signing the informed consent until 3 months after the administration of the last study vaccine. Use of hormonal contraception should start at least 28 days before the first administration of study vaccine. The investigator should evaluate the potential for contraceptive method failure (eg, noncompliance, recently initiated) in relationship to the first vaccination.

Highly effective methods for this study include:

1. hormonal contraception;

a) combined (estrogen and progestogen containing) hormonal contraception associated with inhibition of ovulation (oral, intravaginal, or transdermal)

b) progestogen-only hormonal contraception associated with inhibition of ovulation (oral, injectable, or implantable)

2. intrauterine device;

3. intrauterine hormone-releasing system;

4. bilateral tubal occlusion/ligation procedure;

5. vasectomized partner (the vasectomized partner should be the sole partner for that participant);

6. sexual abstinence*.

*Sexual abstinence is considered an effective method only if defined as refraining from heterosexual intercourse from signing the informed consent until 3 months after the last dose of study vaccine. The reliability of sexual abstinence needs to be evaluated in relation to the duration of the study and the preferred and usual lifestyle of the participant.

8 All participants who were born female and are of childbearing potential must:

a. Have a negative highly sensitive urine pregnancy test at screening

b. Have a negative highly sensitive urine pregnancy test on the day of vaccination prior to each study vaccine administration.

9. Participant agrees to not donate or receive bone marrow, blood, and blood products from the administration of the study vaccine until 3 months after receiving the study vaccines.

10. Participant must be willing to provide verifiable identification to be contacted and to contact the investigator during the study.

11. Participant must be able to read, understand, and complete questionnaires in the participant’s diary.

12. Participant must have access to a consistent means of contact either by telephone contact or e-mail/computer.

**Exclusion Criteria**

Any potential participant who meets any of the following criteria will be excluded from participating in the study:

1. Participant has a clinically significant acute illness (this does not include minor illnesses such as diarrhoea or mild upper respiratory tract infection) or temperature ≥38.0ºC (100.4°F) within 24 hours prior to the planned dose of study vaccine; randomization at a later date is permitted at the discretion of the investigator.

2 Participant has a history of malignancy within 1 year before screening (exceptions are squamous and basal cell carcinomas of the skin and carcinoma in situ of the cervix, or malignancies considered cured with minimal risk of recurrence per investigator’s clinical judgment).

3. Participant has a known allergy or history of anaphylaxis or other serious adverse reactions to vaccines or their excipients (including specifically the excipients of the study vaccine)

4. Participant has a history of severe allergic reactions (eg, anaphylaxis) to any component of the seasonal quadrivalent influenza vaccines, including egg protein, or following a previous dose of any influenza vaccine.

5 Participant has an abnormal function of the immune system resulting from:

a. Clinical conditions (eg, autoimmune disease or immunodeficiency) are expected to have an impact on the immune response elicited by the study vaccine. Participants with autoimmune diseases (eg, autoimmune thyroiditis, autoimmune inflammatory rheumatic diseases such as rheumatoid arthritis and type 1 diabetes) that are stable and controlled without the use of systemic immunomodulators and glucocorticoids may be enrolled at the discretion of the investigator.

Note: Non-immunomodulatory treatment is allowed as well as steroids at a non-immunosuppressive dose or route of administration.

b. Chronic or recurrent use of systemic corticosteroids within 6 months before administration of the study vaccine and during the treatment period of the study at immunosuppressive doses.

b.1 An immunosuppressive steroid dose is considered as >20 mg prednisone or equivalent daily for 2 consecutive weeks.

Note: Ocular, topical, and inhaled steroids are allowed.

c. Administration of antineoplastic and immunomodulating agents or radiotherapy within 6 months before administration of the first study vaccine and during the treatment period of the study.

6. Participant has a history of any neurological disorders or seizures including Guillain-Barré syndrome, with the exception of febrile seizures during childhood.

8. Participant received treatment with immunoglobulins within 3 months or exogenous blood products (autologous blood transfusions are not exclusionary) or blood products within 4 months before the administration of the first study vaccine or plans to receive such treatment during treatment period of the study.

Note: The use of monoclonal antibodies (MABS) before administration of study vaccine and during the study conduct is permitted with the exception of those targeting T cells (anti-CD3, CD4, CD33, and CD52), B cells (anti-CD45, CD19, CD20, CD22, CD27, CD38, and CD138) and check point inhibitors (PD-1, PDL-1 and CTLA-4). Examples of not allowed MABS are: Muromonab-CD3, Gemtuzumab ozogamicin, Alemtuzumab, Bevacizumab, Rituximab, Ofatumumab, Ocrelizumab, Tositumomab, Veltuzumab, Obinutuzumab, Epratuzumab, Pembrolizumab, Nivolumab, Atezolizumab, avelumab, durvalumab and CTLA-4 targeting Ipilimumab. Participants receiving these MABS in the 2 months before the planned administration of the first study vaccine or who plan to receive these MABS during the treatment period of the study will be excluded from participating in the study.

9. Participant has history of TTS or heparin-induced thrombocytopenia and thrombosis (HITT).

10. Participant has history of capillary leak syndrome.

11. Participant received or plans to receive:

a. Licensed live attenuated vaccines - within 28 days before or after planned administration of the first or subsequent study vaccines.

b. Other licensed (not live) vaccines - within 14 days before or after planned administration of the first or subsequent study vaccines.

12 Participant received a licensed/registered SARS-CoV-2 vaccine less than 6 months prior to first study vaccination or during the course of this study (other than study vaccination).

13. Participant received vaccination with a seasonal influenza vaccine for the current influenza season in the Northern Hemisphere.

14. Participant received an investigational drug (including any investigational agent for COVID-19 prophylaxis) or used an invasive investigational medical device within 30 days or received an investigational vaccine within 6 months before the administration of the study vaccine or is currently enrolled or plans to participate in another investigational study during the course of this study.

Note: Participation in an observational clinical study (ie, without intervention) is allowed at the investigator’s discretion.

15. Participant is a woman who is pregnant, or breastfeeding, or planning to become pregnant while enrolled in this study or within 3 months after the administration of the last study vaccine.

16. Participant has a history of an underlying clinically significant acute or chronic medical condition or physical examination findings for which, in the opinion of the investigator, participation would not be in the best interest of the participant (eg, compromise the well-being) or that could prevent, limit, or confound the protocol-specified assessments.

17. Participant had or plans to have major surgery (per the investigator’s judgment) within 4 weeks before the administration of the first study vaccine or will not have recovered from surgery at the time of vaccination.

18. Participant has a contraindication to IM injections and blood draws (eg, bleeding disorders).

19. Participant is an employee of the investigator or study site, with direct involvement in the proposed study or other studies under the direction of that investigator or study site, as well as family members of the employees or the investigator, or an employee of the sponsor.

20. Participant has chronic active hepatitis B or hepatitis C infection per medical history.

21. Participant has had a major psychiatric illness or drug or alcohol abuse which in the investigator’s opinion would compromise the participant’s safety or compliance with the study procedures.

22. Participant cannot communicate reliably with the investigator.

23. Participant who, in the opinion of the investigator, is unlikely to adhere to the requirements of the study, or is unlikely to complete the full course of vaccination and observation.

24. Participant has a positive diagnostic test result for current (viral RNA detection) SARS-CoV-2 infection prior to vaccine administration on Day 1.

**Study vaccines**

Each dose of *Afluria* Quadrivalent (Seqirus) contained 60 µg of hemagglutinin: 15 µg of each of 4 influenza strains (2 influenza A strains and 2 influenza B strains). The two influenza A strains were: A/Victoria/2570/2019 IVR-215 (an A/Victoria/2570/2019 [H1N1]pdm09-like virus) and A/Cambodia/e0826360/2020 IVR-224 (an A/Cambodia/e0826360/2020 [H3N2]-like virus). The two influenza B strains were: B/Victoria/705/2018 BVR-11 (a B/Washington/02/2019-like virus) and B/Phuket/3073/2013 405 BVR-1B (a B/Phuket/3073/2013-like virus).

Each dose of *Fluzone* High-dose Quadrivalent (Sanofi Pasteur Inc) contained 240 µg of hemagglutinin: 60 µg of each of 4 influenza strains (2 influenza A strains and 2 influenza B strains). The two influenza A strains were: A/Victoria/2570/2019 IVR-215 (H1N1) and A/Tasmania/503/2020 IVR-221 (an A/Cambodia/e0826360/2020-like virus) (H3N2). The two influenza B strains were: B/Washington/02/2019 (B Victoria lineage) and B/Phuket/3073/2013 (B Yamagata lineage).

**Sample size**

Sample size calculations are performed under the following assumptions:

- No effect of coadministration of Ad26.COV2.S vaccine and seasonal influenza vaccine on the immune response against influenza as measured by HI antibody titres against the 4 influenza vaccine strains at 28 days after the administration of seasonal influenza vaccine
- The use of Afluria Quadrivalent for standard-dose (adults ≥18 to <64 years of age and older adults ≥65 years of age)
- A standard deviation of between 0.53 for the standard-dose at the log_10_ scale for HI antibody titres against the 4 influenza vaccine strains at 28 days after the administration of seasonal influenza vaccine (with or without Ad26.COV2.S)
- No effect of coadministration of Ad26.COV2.S vaccine and seasonal influenza vaccine on the immune response against SARS-COV-2 as measured by S-ELISA at 28 days after the administration of Ad26.COV2.S vaccine
- A standard deviation of 0.50 at the log_10_ scale for S-ELISA at 28 days after the administration of Ad26.COV2.S vaccine (with or without seasonal influenza vaccine)
- Log transformed (log_10_ scale) immune response data are normally distributed
- A non-inferiority margin of 1.5
- 2-sided α of 5%

A total of approximately 305 (standard-dose) participants per group are needed to have 97.45% power to show non-inferiority in HI antibody titres for each influenza vaccine strain. The sample size accounts for exclusions from the per protocol set, drop-outs and missing samples resulting in a total sample size up to approximately 610 participants who have completed a primary COVID-19 vaccine series. The N margin was ~10% (~30 per group).

With this sample size, the overall power to show non-inferiority in HI antibody titres against each of the 4 influenza vaccine strains at 28 days after the administration of seasonal influenza vaccine as well as non-inferiority in S-ELISA at 28 days after the administration of Ad26.COV2.S vaccine is at least 90%.

**Table S1**  Anti-S antibodies (ELISA Units per ml) in groups that received *standard dose* influenza vaccine descriptive statistics (Per Protocol SARS.CoV.2 Immunogenicity Set)

|  | **Coad_­SD**  **N=301** | **Control_SD**  **N=280** |
| --- | --- | --- |
| Baseline |  |  |
| Geometric mean (95% CI) | 4422 (3702; 5282) |  |
| Positive sample n (%) (95% CI) | 300 (99·7%) (98·2; 100) | - |
| Day 29 |  |  |
| Geometric mean (95% CI) | 22531 (20140; 25205) | 4297 (3511; 5260) |
| Positive sample n (%) (95% CI) | 257 (100%) (98·6; 100) | 247 (98·8%) (96·5; 99·8) |
| Geometric mean increase (95% CI) from Baseline | 5·3 (4·5; 6·3) |  |
| Responders n/N* (%) (95% CI) | 139/257 (54·1%) (47·8; 60·3) | - |
| Day 57 |  |  |
| Geometric mean (95% CI) | 21100 (18431; 24155) | 25035 (22189; 28246) |
| Positive sample n (%) (95% CI) | 193 (100%) (98·1; 100) | 209 (100%) (98·3; 100) |
| Geometric mean increase (95% CI) from Baseline | 4·6 (3·8; 5·6) | 5·7 (4·7; 7·0) |
| Responders n/N* (%) (95% CI) | 98/193 (50·8%) (43·5; 58·0) | 120/207 (58·0%) (50·9; 64·8) |
| Day 181 |  |  |
| Geometric mean (95% CI) | 14238 (12140; 16698) | 16072 (13623; 18960) |
| Positive sample n (%) (95% CI) | 165 (100%) (97·8; 100) | 155 (100%) (97·6; 100) |
| Geometric mean increase (95% CI) from Baseline | 3·1 (2·4; 3·9) | 2·9 (2·2; 3·7) |
| Responders n/N* (%) (95% CI) | 75/165 (45·5%) (37·7; 53·4) | 60/152 (39·5%) (31·6; 47·7) |

CI, confidence interval; N, number of participants with data

Responders are participants with a postbaseline sample >LLOQ in participants with a baseline sample value ≤LLOQ; or a ≥4-fold increase from the baseline sample when the baseline value was >LLOQ.

A positive sample refers to result that was strictly greater than the LLOQ (50.3)

Exact Clopper-Pearson 95% confidence intervals are shown for Positive sample and Responders.

**Table S2**  Haemagglutinin inhibition response against influenza vaccine strains; *standard dose*: descriptive statistics (Per Protocol Influenza Immunogenicity Set)

|  | **Coad_SD**  **N=359** | **Control_SD**  **N=367** | **Difference (95% CI)** | **Coad_SD**  **N=359** | **Control_SD**  **N=367** | **Difference (95% CI)** |
| --- | --- | --- | --- | --- | --- | --- |
|  | **A/Victoria (H1N1)** | | | **A/Cambodia (H3N2)** | | |
| **Baseline** |  |  |  |  |  |  |
| GM (95% CI) | 44 (39, 49) | 45 (39, 51) |  | 42 (37, 48) | 44 (39, 50) |  |
| Positive sample n (%) (95% CI) | 304 (84·9%) (80·8, 88·5) | 308 (83·9%) (79·8, 87·5) |  | 287 (80·2%) (75·7, 84·2) | 301 (82·0%) (77·7, 85·8) |  |
| **Day 29** |  |  |  |  |  |  |
| GM (95% CI) | 306 (271, 346) | 393 (348, 445) |  | 134 (119, 150) | 165 (147, 186) |  |
| Positive sample n (%) (95% CI) | 318 (99·4%) (97·8, 99·9) | 331 (99·4%) (97·8, 99·9) |  | 316 (98·8%) (96·8, 99·7) | 329 (98·8%) (97·0, 99·7) |  |
| SP n (%) (95% CI) | 313 (97·8%) (95·5, 99·1) | 324 (97·3%) (94·9, 98·8) | 0·5% (-1·9, 2·9) | 297 (92·8%) (89·4, 95·4) | 311 (93·4%) (90·2, 95·8) | -0·6% (-4·5, 3·3) |
| SC (%) (95% CI) | 205 (64·1%) (58·5, 69·3) | 233 (70·0%) (64·7, 74·8) | -5·9% (-13·1, 1·3) | 125 (39·1%) (33·7, 44·6) | 156 (46·8%) (41·4, 52·4) | -7·8% (-15·4, -0·2) |
| GMI (95% CI) | 6·8 (5·8, 7·9) | 8·3 (7·1, 9·7) |  | 3·0 (2·7, 3·4) | 3·5 (3·1, 4·0) |  |
| **Day 57** |  |  |  |  |  |  |
| GM (95% CI) | 174 (154, 197) | 207 (181, 236) |  | 124 (110, 141) | 146 (130, 165) |  |
| Positive sample n (%) (95% CI) | 260 (99·2%) (97·3, 99·9) | 280 (99·3%) (97·5, 99·9) |  | 257 (98·1%) (95·6, 99·4) | 276 (97·9%) (95·4, 99·2) |  |
| SP n (%) (95% CI) | 253 (96·6%) (93·6, 98·4) | 270 (95·7%) (92·7, 97·8) | 0·8% (-2·4, 4·0) | 240 (91·6%) (87·6, 94·7) | 261 (92·6%) (88·8, 95·3) | -1·0% (-5·5, 3·6) |
| SC (%) (95% CI) | 135 (51·5%) (45·3, 57·7) | 155 (55·0%) (49·0, 60·9) | -3·4% (-11·8, 4·9) | 106 (40·5%) (34·5, 46·7) | 120 (42·6%) (36·7, 48·6) | -2·1% (-10·4, 6·2) |
| GMI (95% CI) | 3·6 (3·1, 4·3) | 4·4 (3·7, 5·3) |  | 2·8 (2·4, 3·2) | 3·3 (2·8, 3·8) |  |
| **Day 181** |  |  |  |  |  |  |
| GM (95% CI) | 140 (123, 160) | 156 (136, 179) |  | 83 (72, 96) | 104 (90, 119) |  |
| Positive sample n (%) (95% CI) | 223 (99·1%) (96·8, 99·9) | 222 (98·7%) (96·2, 99·7) |  | 216 (96·0%) (92·5, 98·2) | 218 (96·9%) (93·7, 98·7) |  |
| SP n (%) (95% CI) | 212 (94·2%) (90·3, 96·9) | 208 (92·4%) (88·2, 95·5) | 1·8% (-2·8, 6·4) | 186 (82·7%) (77·1, 87·4) | 204 (90·7%) (86·1, 94·1) | -8·0% (-14·2, -1·8) |
| SC (%) (95% CI) | 96 (42·7%) (36·1, 49·4) | 103 (45·8%) (39·1, 52·5) | -3·1% (-12·3, 6·1) | 51 (22·7%) (17·4, 28·7) | 70 (31·1%) (25·1, 37·6) | -8·4% (-16·6, -0·3) |
| GMI (95% CI) | 2·9 (2·5, 3·5) | 3·2 (2·7, 3·8) |  | 1·8 (1·6, 2·1) | 2·4 (2·0, 2·7) |  |
|  | **B/Victoria (B/Victoria)** | | | **B/Phuket (B/Yamagata)** | | |
| **Baseline** |  |  |  |  |  |  |
| GM (95% CI) | < LLOQ | < LLOQ |  | < LLOQ (< LLOQ, 11) | 10 (< LLOQ, 11) |  |
| Positive sample n (%) (95% CI) | 93 (26·0%) (21·5, 30·8) | 103 (28·1%) (23·5, 33·0) |  | 117 (32·7%) (27·8, 37·8) | 116 (31·6%) (26·9, 36·6) |  |
| **Day 29** |  |  |  |  |  |  |
| GM (95% CI) | 38 (34, 43) | 38 (33, 43) |  | 32 (29, 36) | 33 (30, 37) |  |
| Positive sample n (%) (95% CI) | 263 (82·2%) (77·5, 86·2) | 268 (80·5%) (75·8, 84·6) |  | 252 (78·8%) (73·9, 83·1) | 270 (81·1%) (76·5, 85·1) |  |
| SP n (%) (95% CI) | 182 (56·9%) (51·2, 62·4) | 191 (57·4%) (51·8, 62·7) | -0·5% (-8·1, 7·1) | 167 (52·2%) (46·6, 57·8) | 183 (55·0%) (49·4, 60·4) | -2·8% (-10·4, 4·9) |
| SC (%) (95% CI) | 137 (42·8%) (37·3, 48·4) | 145 (43·5%) (38·1, 49·1) | -0.7% (-8.3, 6.9) | 113 (35·3%) (30·1, 40·8) | 123 (36·9%) (31·7, 42·4) | -1·6% (-9·0, 5·7) |
| GMI (95% CI) | 3·0 (2·7, 3·4) | 2·9 (2·6, 3·3) |  | 2·5 (2·2, 2·7) | 2·6 (2·4, 2·9) |  |
| **Day 57** |  |  |  |  |  |  |
| GM (95% CI) | 40 (36, 45) | 47 (41, 53) |  | 23 (20, 26) | 25 (22, 28) |  |
| Positive sample n (%) (95% CI) | 222 (84·7%) (79·8, 88·9) | 251 (89·0%) (84·8, 92·4) |  | 175 (66·8%) (60·7, 72·5) | 203 (72·0%) (66·4, 77·1) |  |
| SP n (%) (95% CI) | 163 (62·2%) (56·0, 68·1) | 186 (66·0%) (60·1, 71·5) | -3·7% (-11·8, 4·3) | 103 (39·3%) (33·4, 45·5) | 115 (40·8%) (35·0, 46·8) | -1·5% (-9·7, 6·8) |
| SC (%) (95% CI) | 124 (47·3%) (41·2, 53·6) | 149 (52·8%) (46·8, 58·8) | -5·5% (-13·9, 2·9) | 64 (24·4%) (19·3, 30·1) | 81 (28·7%) (23·5, 34·4) | -4·3% (-11·7, 3·1) |
| GMI (95% CI) | 3·1 (2·8, 3·5) | 3·5 (3·1, 4·0) |  | 1·8 (1·6, 2·0) | 2·1 (1·8, 2·3) |  |
| **Day 181** |  |  |  |  |  |  |
| GM (95% CI) | 22 (20, 25) | 23 (21, 26) |  | 16 (14, 18) | 18 (16, 20) |  |
| Positive sample n (%) (95% CI) | 169 (75·1%) (68·9, 80·6) | 161 (71·6%) (65·2, 77·4) |  | 120 (53·3%) (46·6, 60·0) | 137 (60·9%) (54·2, 67·3) |  |
| SP n (%) (95% CI) | 81 (36·0%) (29·7, 42·6) | 87 (38·7%) (32·3, 45·4) | -2·7% (-11·6, 6·3) | 58 (25·8%) (20·2, 32·0) | 63 (28·0%) (22·2, 34·4) | -2·2% (-10·4, 6·0) |
| SC (%) (95% CI) | 50 (22·2%) (17·0, 28·2) | 55 (24·4%) (19·0, 30·6) | -2·2% (-10·0, 5·6) | 29 (12·9%) (8·8, 18·0) | 35 (15·6%) (11·1, 21·0) | -2·7% (-9·1, 3·8) |
| GMI (95% CI) | 1·9 (1·7, 2·1) | 1·9 (1·7, 2·1) |  | 1·4 (1·3, 1·5) | 1·5 (1·4, 1·7) |  |

CI, confidence interval; GM, geometric mean; GMI, geometric mean increase from baseline; N, number of participants with data SC, seroconversion; SP, seroprotection.

A positive sample refers to result that was strictly greater than the LLOQ (10)

Responders are participants with a postbaseline sample >LLOQ in participants with a baseline sample value ≤LLOQ; or a ≥4-fold increase from the baseline sample when the baseline value was >LLOQ.

Seroconversion was defined as an HI titre ≥1:40 in participants with a pre-vaccination HI titre of <1:10, or a ≥4-fold increase in HI titre in participants with a pre-vaccination HI titre of ≥1:10.

Seroprotection was defined as a post-vaccination HI titre ≥1:40.

Exact Clopper-Pearson 95% confidence intervals are shown for Positive sample and Responders.

**Table S3**  Anti-S antibodies (ELISA Units per ml) in groups that received *high dose* influenza vaccine descriptive statistics (Per Protocol SARS.CoV.2 Immunogenicity Set)

|  | **Coad_­HD**  **N=44** | **Control_HD**  **N=42** |
| --- | --- | --- |
| Baseline |  |  |
| Geometric mean (95% CI) | 3521 (2125; 5836) |  |
| Positive sample n (%) (95% CI) | 43 (97·7%) (88·0; 99·9) | - |
| Day 29 |  |  |
| Geometric mean (95% CI) | 17569 (13391; 23051) | 2208 (1140; 4276) |
| Positive sample n (%) (95% CI) | 39 (100·0%) (91·0; 100·0) | 36 (94·7%) (82·3; 99·4) |
| Geometric mean increase (95% CI) from Baseline | 4·3 (2·7; 6·8) |  |
| Responders n/N* (%) (95% CI) | 21/39 (53·8%) (37·2; 69·9) | - |
| Day 57 |  |  |
| Geometric mean (95% CI) | 20133 (14324; 28299) | 20743 (12732; 33794) |
| Positive sample n (%) (95% CI) | 31 (100·0%) (88·8; 100·0) | 33 (100·0%) (89·4; 100·0) |
| Geometric mean increase (95% CI) from Baseline | 4·4 (2·7; 7·2) | 8·3 (4·7; 14·4) |
| Responders n/N* (%) (95% CI) | 17/31 (54·8%) (36·0; 72·7) | 23/33 (69·7%) (51·3; 84·4) |
| Day 181 |  |  |
| Geometric mean (95% CI) | 13861 (8565; 22432) | 18854 (10954; 32450) |
| Positive sample n (%) (95% CI) | 22 (100·0%) (84·6; 100·0) | 26 (100·0%) (86·8; 100·0) |
| Geometric mean increase (95% CI) from Baseline | 3·8 (2·2; 6·7) | 6·3 (3·2; 12·4) |
| Responders n/N* (%) (95% CI) | 9/22 (40·9%) (20·7; 63·6) | 19/26 (73·1%) (52·2; 88·4) |

CI, confidence interval; N, number of participants with data

A positive sample refers to result that was strictly greater than the LLOQ (50.3)

Responders are participants with a postbaseline sample >LLOQ in participants with a baseline sample value ≤LLOQ; or a ≥4-fold increase from the baseline sample when the baseline value was >LLOQ.

Exact Clopper-Pearson 95% confidence intervals are shown for Positive sample and Responders.

**Table S4**  Haemagglutinin inhibition response against influenza vaccine strains; *high* *dose*: descriptive statistics (Per Protocol Influenza Immunogenicity Set)

|  | **Coad_HD**  **N=46** | **Control_HD**  **N=45** | **Difference (95% CI)** | **Coad_HD**  **N=46** | **Control_HD**  **N=45** | **Difference (95% CI)** |
| --- | --- | --- | --- | --- | --- | --- |
|  | **A/Victoria (H1N1)** | | | **A/Tasmania (H3N2)** | | |
| **Baseline** |  |  |  |  |  |  |
| GM (95% CI) | 51 (39, 65) | 62 (49, 78) |  | 41 (29, 58) | 69 (50, 95) |  |
| Positive sample n (%) (95% CI) | 43 (93·5%) (82·1, 98·6) | 45 (100%) (92·1, 100) |  | 39 (84·8%) (71·1, 93·7) | 42 (93·3%) (81·7, 98·6) |  |
| **Day 29** |  |  |  |  |  |  |
| GM (95% CI) | 286 (204, 400) | 484 (369, 636) |  | 284 (200, 402) | 509 (365, 711) |  |
| Positive sample n (%) (95% CI) | 43 (100%) (91·8, 100) | 41 (100%) (91·4, 100) |  | 43 (100%) (91·8, 100) | 41 (100%) (91·4, 100) |  |
| SP n (%) (95% CI) | 43 (100%) (91·8, 100) | 41 (100%) (91·4, 100) | - | 42 (97·7%) (87·7, 99·9) | 41 (100%) (91·4, 100) | -2·3% (-6·8, 2·2) |
| SC (%) (95% CI) | 28 (65·1%) (49·1, 79·0) | 29 (70·7%) (54·5, 83·9) | -5·6% (-25·5, 14·3) | 31 (72·1%) (56·3, 84·7) | 29 (70·7%) (54·5, 83·9) | 1·4% (-18·0, 20·7) |
| GMI (95% CI) | 5·8 (3·9, 8·5) | 7·9 (5·6, 11·2) |  | 6·5 (4·4, 9·5) | 7·0 (4·5, 10·8) |  |
| **Day 57** |  |  |  |  |  |  |
| GM (95% CI) | 222 (145, 339) | 345 (255, 467) |  | 171 (120, 244) | 253 (188, 341) |  |
| Positive sample n (%) (95% CI) | 36 (100%) (90·3, 100) | 37 (100%) (90·5, 100) |  | 36 (100%) (90·3, 100) | 37 (100%) (90·5, 100) | -8·3% (-17·4, 0·7) |
| SP n (%) (95% CI) | 34 (94·4%) (81·3, 99·3) | 37 (100%) (90·5, 100) | -5·6% (-13·0, 1·9) | 33 (91·7%) (77·5, 98·2) | 37 (100%) (90·5, 100) | 1·4% (-21·6, 24·3) |
| SC (%) (95% CI) | 19 (52·8%) (35·5, 69·6) | 24 (64·9%) (47·5, 79·8) | -12·1% (-34·5, 10·3) | 18 (50·0%) (32·9, 67·1) | 18 (48·6%) (31·9, 65·6) |  |
| GMI (95% CI) | 4·7 (3·0, 7·4) | 5·9 (4·0, 8·6) |  | 4·0 (2·6, 6·2) | 3·5 (2·3, 5·6) |  |
| **Day 181** |  |  |  |  |  |  |
| GM (95% CI) | 115 (76, 172) | 144 (105, 196) |  | 154 (107, 222) | 201 (149, 270) |  |
| Positive sample n (%) (95% CI) | 27 (100%) (87·2, 100) | 32 (100%) (89·1, 100) | -8·0% (-21·3, 5·3) | 27 (100%) (87·2, 100) | 32 (100%) (89·1, 100) | -3·7% (-10·8, 3·4) |
| SP n (%) (95% CI) | 24 (88·9%) (70·8, 97·6) | 31 (96·9%) (83·8, 99·9) | 13·2% (-11·5, 37·9) | 26 (96·3%) (81·0, 99·9) | 32 (100%) (89·1, 100) | 5·0% (-20·6, 30·6) |
| SC (%) (95% CI) | 12 (44·4%) (25·5, 64·7) | 10 (31·3%) (16·1, 50·0) |  | 14 (51·9%) (31·9, 71·3) | 15 (46·9%) (29·1, 65·3) |  |
| GMI (95% CI) | 2·4 (1·4, 3·9) | 2·6 (1·8, 3·8) |  | 3·3 (2·3, 5·0) | 3·1 (2·0, 4·9) |  |
|  | **B/Washington (B/Victoria)** | | | **B/Phuket (B/Yamagata)** | | |
| **Baseline** |  |  |  |  |  |  |
| GM (95% CI) | 19 (15, 25) | 16 (13, 21) |  | 13 (10, 17) | 12 (10, 15) |  |
| Positive sample n (%) (95% CI) | 28 (60·9%) (45·4, 74·9) | 25 (55·6%) (40·0, 70·4) |  | 20 (43·5%) (28·9, 58·9) | 21 (46·7%) (31·7, 62·1) |  |
| **Day 29** |  |  |  |  |  |  |
| GM (95% CI) | 61 (46, 81) | 75 (53, 106) |  | 38 (29, 50) | 39 (30, 52) |  |
| Positive sample n (%) (95% CI) | 41 (95·3%) (84·2, 99·4) | 39 (95·1%) (83·5, 99·4) |  | 38 (88·4%) (74·9, 96·1) | 38 (92·7%) (80·1, 98·5) |  |
| SP n (%) (95% CI) | 34 (79·1%) (64·0, 90·0) | 34 (82·9%) (67·9, 92·8) | -3·9% (-20·6, 12·9) | 27 (62·8%) (46·7, 77·0) | 26 (63·4%) (46·9, 77·9) | -0·6% (-21·3, 20·0) |
| SC (%) (95% CI) | 18 (41·9%) (27·0, 57·9) | 22 (53·7%) (37·4, 69·3) | -11·8% (-33·0, 9·4) | 16 (37·2%) (23·0, 53·3) | 14 (34·1%) (20·1, 50·6) | 3·1% (-17·4, 23·5) |
| GMI (95% CI) | 2·9 (2·2, 3·9) | 4·1 (2·8, 6·0) |  | 2·4 (1·9, 3·1) | 2·7 (2·1, 3·5) |  |
| **Day 57** |  |  |  |  |  |  |
| GM (95% CI) | 23 (17, 31) | 33 (22, 49) |  | 23 (18, 31) | 24 (18, 32) |  |
| Positive sample n (%) (95% CI) | 26 (72·2%) (54·8, 85·8) | 28 (75·7%) (58·8, 88·2) |  | 27 (75·0%) (57·8, 87·9) | 29 (78·4%) (61·8, 90·2) |  |
| SP n (%) (95% CI) | 12 (33·3%) (18·6, 51·0) | 18 (48·6%) (31·9, 65·6) | -15·3% (-37·6, 7·0) | 12 (33·3%) (18·6, 51·0) | 13 (35·1%) (20·2, 52·5) | -1·8% (-23·6, 20·0) |
| SC (%) (95% CI) | 5 (13·9%) (4·7, 29·5) | 12 (32·4%) (18·0, 49·8) | -18·5% (-37·4, 0·3) | 5 (13·9%) (4·7, 29·5) | 6 (16·2%) (6·2, 32·0) | -2·3% (-18·7, 14·1) |
| GMI (95% CI) | 1·2 (0·9, 1·7) | 1·8 (1·2, 2·8) |  | 1·6 (1·3, 2·0) | 1·7 (1·3, 2·2) |  |
| **Day 181** |  |  |  |  |  |  |
| GM (95% CI) | 12 (< LLOQ, 17) | 18 (12, 26) |  | 19 (14, 27) | 18 (12, 25) |  |
| Positive sample n (%) (95% CI) | 15 (55·6%) (35·3, 74·5) | 18 (56·3%) (37·7, 73·6) |  | 16 (59·3%) (38·8, 77·6) | 19 (59·4%) (40·6, 76·3) |  |
| SP n (%) (95% CI) | 3 (11·1%) (2·4, 29·2) | 9 (28·1%) (13·7, 46·7) | -17·0% (-36·6, 2·6) | 9 (33·3%) (16·5, 54·0) | 7 (21·9%) (9·3, 40·0) | 11·5% (-11·4, 34·3) |
| SC (%) (95% CI) | 0 | 6 (18·8%) (7·2, 36·4) | -18·8% (-32·3, -5·2) | 1 (3·7%) (0·1, 19·0) | 3 (9·4%) (2·0, 25·0) | -5·7% (-18·0, 6·7) |
| GMI (95% CI) | 0·7 (0·5, 0·9) | 1·2 (0·8, 1·8) |  | 1·2 (0·9, 1·6) | 1·4 (1·0, 1·8) |  |

A positive sample refers to result that was strictly greater than the LLOQ (10)

CI, confidence interval; GM, geometric mean; GMI, geometric mean increase from baseline; N, number of participants with data SC, seroconversion; SP, seroprotection.

Responders are participants with a postbaseline sample >LLOQ in participants with a baseline sample value ≤LLOQ; or a ≥4-fold increase from the baseline sample when the baseline value was >LLOQ.

Seroconversion was defined as an HI titre ≥1:40 in participants with a pre-vaccination HI titre of <1:10, or a ≥4-fold increase in HI titre in participants with a pre-vaccination HI titre of ≥1:10.

Seroprotection was defined as a post-vaccination HI titre ≥1:40.

Exact Clopper-Pearson 95% confidence intervals are shown for Positive sample and Responders.

**Table S5**  Solicited local and systemic adverse events reported within 7 days after vaccination in all study groups, post dose 1 and post-dose 2 combined (Full Analysis Set)

|  | **Coad_­SD**  **N=382** | **Control_SD**  **N=384** | **Coad_­HD**  **N=47** | **Control_HD**  **N=46** |
| --- | --- | --- | --- | --- |
| **Injection site AEs** |  |  |  |  |
| Subjects with 1 or more AEs |  |  |  |  |
| Any | 265 (69.4%) | 275 (71.6%) | 24 (51.1%) | 33 (71.7%) |
| Grade 1 | 174 (45.5%) | 182 (47.4%) | 19 (40.4%) | 28 (60.9%) |
| Grade 2 | 86 (22.5%) | 82 (21.4%) | 5 (10.6%) | 4 (8.7%) |
| Grade 3 | 5 (1.3%) | 11 (2.9%) | 0 | 1 (2.2%) |
| Vaccination site erythema |  |  |  |  |
| Any | 12 (3.1%) | 9 (2.3%) | 1 (2.1%) | 2 (4.3%) |
| Grade 1 | 11 (2.9%) | 9 (2.3%) | 1 (2.1%) | 1 (2.2%) |
| Grade 2 | 1 (0.3%) | 0 | 0 | 1 (2.2%) |
| Vaccination site pain |  |  |  |  |
| Any | 265 (69.4%) | 275 (71.6%) | 24 (51.1%) | 33 (71.7%) |
| Grade 1 | 174 (45.5%) | 182 (47.4%) | 19 (40.4%) | 29 (63.0%) |
| Grade 2 | 86 (22.5%) | 83 (21.6%) | 5 (10.6%) | 3 (6.5%) |
| Grade 3 | 5 (1.3%) | 10 (2.6%) | 0 | 1 (2.2%) |
| Vaccination site swelling |  |  |  |  |
| Any | 12 (3.1%) | 8 (2.1%) | 1 (2.1%) | 0 |
| Grade 1 | 9 (2.4%) | 6 (1.6%) | 1 (2.1%) | 0 |
| Grade 2 | 3 (0.8%) | 1 (0.3%) | 0 | 0 |
| Grade 3 | 0 | 1 (0.3%) | 0 | 0 |
|  |  |  |  |  |
| **Systemic AEs** |  |  |  |  |
| Subjects with 1 or more AEs |  |  |  |  |
| Any | 268 (70.2%) | 274 (71.4%) | 31 (66.0%) | 34 (73.9%) |
| Grade 1 | 98 (25.7%) | 106 (27.6%) | 18 (38.3%) | 24 (52.2%) |
| Grade 2 | 127 (33.2%) | 129 (33.6%) | 12 (25.5%) | 8 (17.4%) |
| Grade 3 | 43 (11.3%) | 39 (10.2%) | 1 (2.1%) | 2 (4.3%) |
| Fatigue |  |  |  |  |
| Any | 226 (59.2%) | 232 (60.4%) | 28 (59.6%) | 27 (58.7%) |
| Grade 1 | 87 (22.8%) | 94 (24.5%) | 18 (38.3%) | 19 (41.3%) |
| Grade 2 | 113 (29.6%) | 115 (29.9%) | 10 (21.3%) | 7 (15.2%) |
| Grade 3 | 26 (6.8%) | 23 (6.0%) | 0 | 1 (2.2%) |
| Headache |  |  |  |  |
| Any | 212 (55.5%) | 209 (54.4%) | 19 (40.4%) | 24 (52.2%) |
| Grade 1 | 101 (26.4%) | 99 (25.8%) | 13 (27.7%) | 17 (37.0%) |
| Grade 2 | 89 (23.3%) | 96 (25.0%) | 6 (12.8%) | 5 (10.9%) |
| Grade 3 | 22 (5.8%) | 14 (3.6%) | 0 | 2 (4.3%) |
| Myalgia |  |  |  |  |
| Any | 209 (54.7%) | 217 (56.5%) | 22 (46.8%) | 22 (47.8%) |
| Grade 1 | 104 (27.2%) | 110 (28.6%) | 18 (38.3%) | 19 (41.3%) |
| Grade 2 | 83 (21.7%) | 93 (24.2%) | 3 (6.4%) | 2 (4.3%) |
| Grade 3 | 22 (5.8%) | 14 (3.6%) | 1 (2.1%) | 1 (2.2%) |
| Nausea |  |  |  |  |
| Any | 116 (30.4%) | 115 (29.9%) | 13 (27.7%) | 10 (21.7%) |
| Grade 1 | 72 (18.8%) | 80 (20.8%) | 9 (19.1%) | 7 (15.2%) |
| Grade 2 | 33 (8.6%) | 30 (7.8%) | 4 (8.5%) | 2 (4.3%) |
| Grade 3 | 11 (2.9%) | 5 (1.3%) | 0 | 1 (2.2%) |
| Pyrexia |  |  |  |  |
| Any | 49 (12.8%) | 38 (9.9%) | 1 (2.1%) | 1 (2.2%) |
| Grade 1 | 27 (7.1%) | 17 (4.4%) | 0 | 1 (2.2%) |
| Grade 2 | 13 (3.4%) | 15 (3.9%) | 1 (2.1%) | 0 |
| Grade 3 | 9 (2.4%) | 6 (1.6%) | 0 | 0 |

Coad_HD group received quadrivalent high dose influenza vaccine and Ad26.COV2.S at Dose 1 and placebo at Dose 2.

Control­_HD group received quadrivalent high dose influenza vaccine and placebo at Dose 1 and Ad26.COV2.S at Dose 2.

Pain: Grade 1 = Aware of symptoms but easily tolerated, does not interfere with activity, discomfort only to touch; Grade 2 = Notable symptoms, requires modification in activity or use of medications, discomfort with movement; Grade 3 = Incapacitating symptoms, inability to do work, school, or usual activities, use of narcotic pain reliever.

Erythema and swelling: Grade 1 = 25–50 mm; Grad 3 = 51–100 mm; Grade 3 = >100 mm

Nausea: Grade 1 = Minimal symptoms, causes minimal or no interference with work, school, or selfcare activities; Grade 2 = Notable symptoms, requires modification in activity or use of medications, does not result in loss of work, school, or cancellation of social activities; Grade 3 = Incapacitating symptoms, requires bed rest and/or results in loss of work, school, or cancellation of social activities

Fever: Grade 1 = 38.0–38.4°C; Grade 2 = 38.5–38.9°C; Grade 3 = 39.0– 40.0°C; Grade 4 = >40.0°C

Other symptoms: Grade 1= Minimal symptoms causing no or minimal interference with usual social and functional activities; Grade 2 = Notable symptoms causing greater than minimal interference with usual social and functional activities (may require use of medications); Grade 3 = Severe symptoms causing inability to perform usual social and functional activities and requires medical intervention (may require use of narcotic pain reliever); Grade 4: Hospitalization, inability to perform basic self-care functions.

**Figure S1** Individual plots of hemagglutinin inhibition (HI) antibody response against each of the 4 influenza vaccine strains by SARS-CoV-2 serostatus* (positive or negative) at baseline; *standard dose* (Per Protocol Influenza Immunogenicity Set)

Concomitant administration of the Ad26.COV2.S and influenza vaccine (standard-dose) induced similar H3N2, B/Victoria, and B/Yamagata HI GMTs 28 days after vaccination versus to administration of the influenza vaccine (standard dose) alone, irrespective of SARS-CoV-2 serostatus at baseline. There was a trend towards a lower H1N1 HI GMT in the Coad_SD group versus the Control_SD group in participants who were SARS-CoV-2 seronegative at baseline that was less pronounced in those who were initially SARS-CoV-2 seropositive. HI titres induced in SARS-CoV-2 seronegative and SARS-CoV-2 seropositive participants were similar over the duration of the study.

Seroconversion and seroprotection rates against vaccine influenza strains were comparable in the Coad_SD and Control_SD groups in both participants who were SARS CoV-2 seropositive and seronegative at baseline.

GMT, geometric mean titre; LLOQ, lower limit of quantitation; ULOQ, upper limit of quantitation

*serostatus determined by N-ELISA or S-ELISA at baseline.

Dotted black line shows a titre of 40. Mean and 95% CIs shown

**A/Victoria (H1N1)**


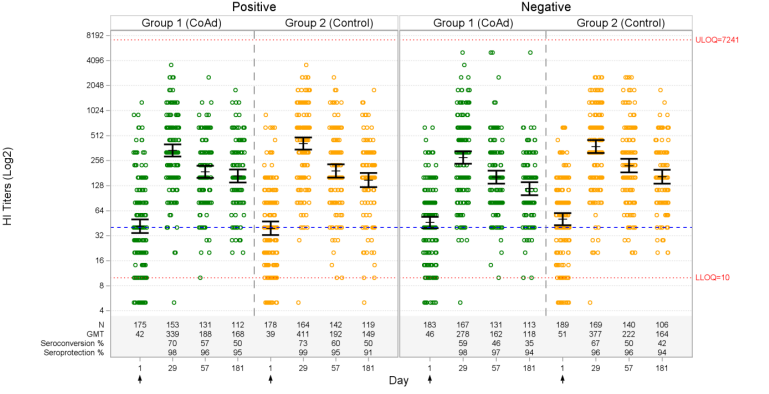


**A/Cambodia (H3N2)**


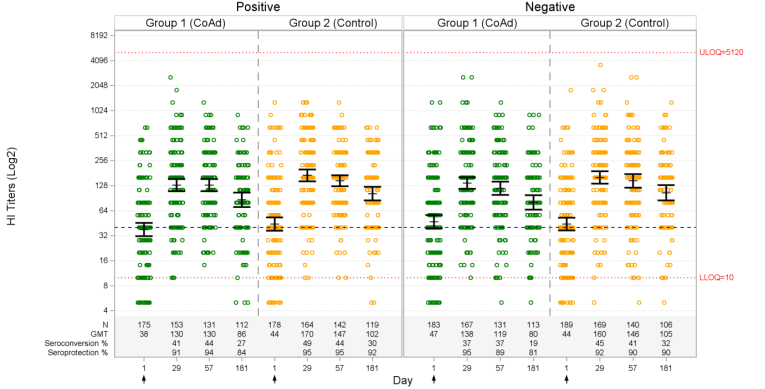


**B/Victoria (B/Victoria)**


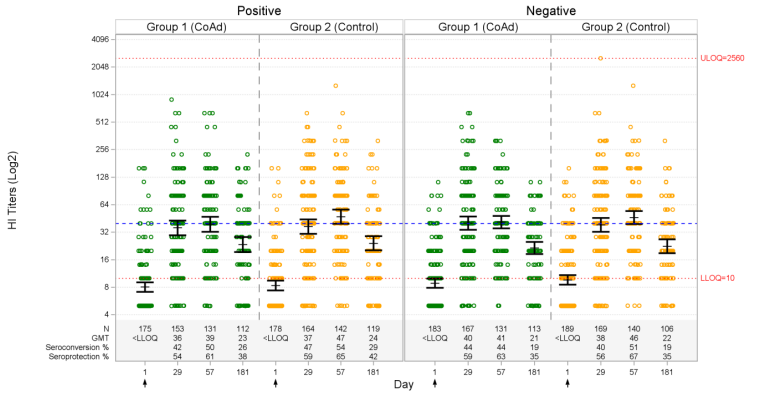


**B/Phuket (B/Yamagata)**


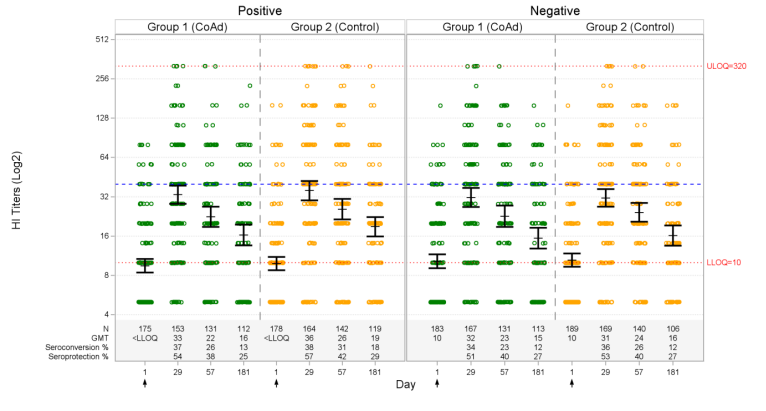


**Figure S2** Individual plots of Spike-binding antibody responses by SARS-CoV-2 serostatus* (positive or negative) at baseline; *standard dose groups* (Per Protocol SARS-CoV-2 Immunogenicity Set)

Anti-S antibody GMCs and responder rates were comparable in the Coad_SD and Control_SD groups in both participants who were SARS CoV-2 seropositive and seronegative at baseline.

GMT, geometric mean titre; LLOQ, lower limit of quantitation; ULOQ, upper limit of quantitation

*serostatus determined by N-ELISA or S-ELISA at baseline.

Mean and 95% CIs shown


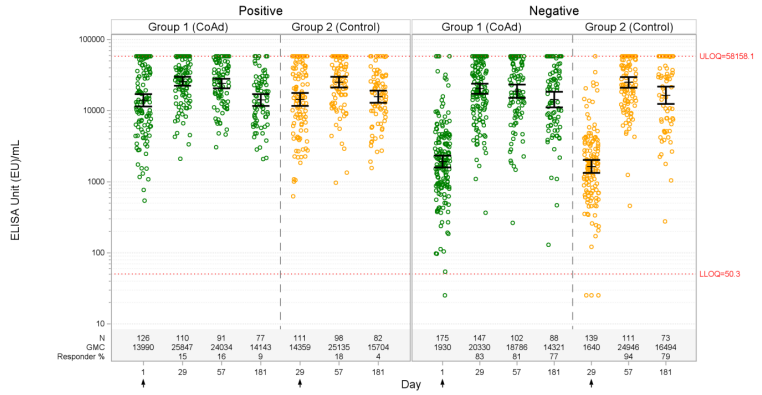


**Figure S3** Individual plots of hemagglutinin inhibition (HI) antibody response against each of the 4 influenza vaccine strains by SARS-CoV-2 serostatus* (positive or negative) at baseline; *high dose* (Per Protocol Influenza Immunogenicity Set)

HI GMTs tended to be higher in the Control_HD group than the Coad-HD group in initially seropositive participants for H1N1 and H3N2 HI strains, although the number of participants was limited. HI GMTs were similar in the Coad-HD and Control_HD groups for all vaccine influenza strains for initially SARS-CoV-2 seronegative participants.

Seroconversion and seroprotection rates against vaccine influenza strains were comparable in the Coad_HD and Control_HD groups in both participants who were SARS CoV-2 seropositive and seronegative at baseline.

GMT, geometric mean titre; LLOQ, lower limit of quantitation; ULOQ, upper limit of quantitation

*serostatus determined by N-ELISA or S-ELISA at baseline.

Dotted black line shows a titre of 40. Mean and 95% CIs shown

**A/Victoria (H1N1)**


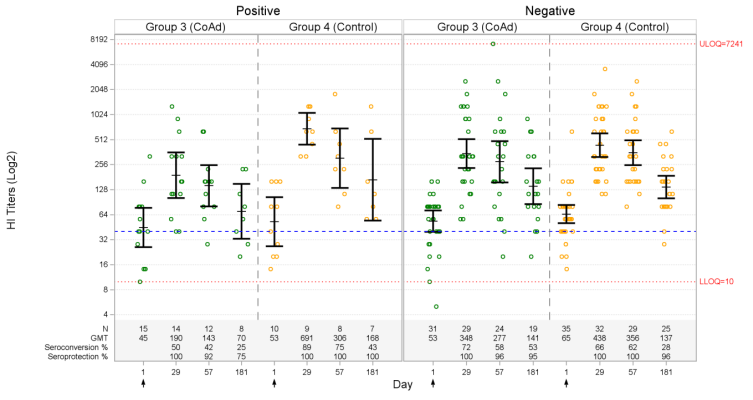


**A/Tasmania (H3N2)**


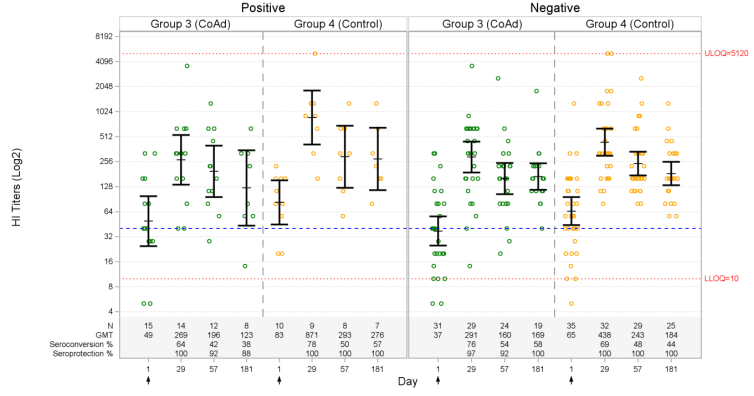


**B/Washington (B/Victoria)**


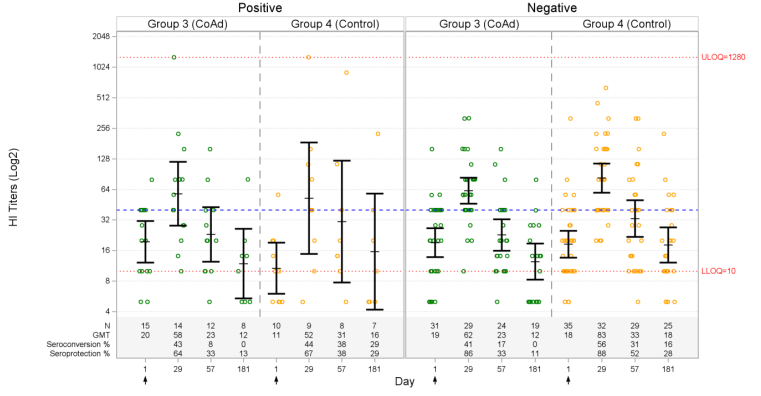


**B/Phuket (B/Yamagata)**


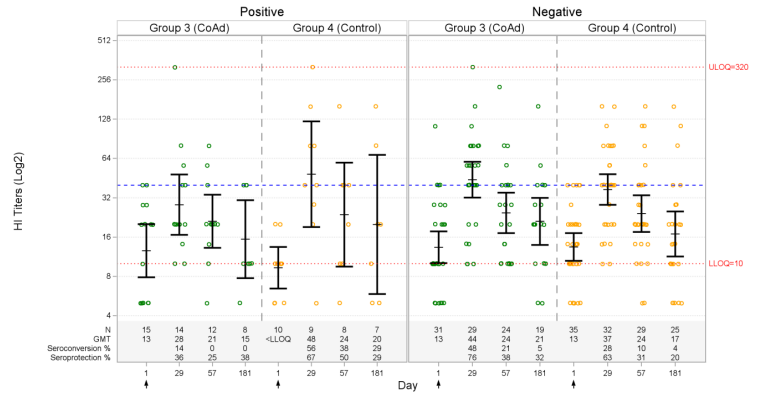


**Figure S4** Individual plots of Spike-binding antibody responses by SARS-CoV-2 serostatus* (positive or negative) at baseline; *high dose groups* (Per Protocol SARS-CoV-2 Immunogenicity Set)

Anti-S antibody GMCs to be higher in the Control_HD group than the Coad_HD in participants who were SARS CoV-2 seropositive at baseline but were similar in participants who were seronegative at baseline. Responder rates were similar in both populations.

GMT, geometric mean titre; LLOQ, lower limit of quantitation; ULOQ, upper limit of quantitation

*serostatus determined by N-ELISA or S-ELISA at baseline.

Mean and 95% CIs shown


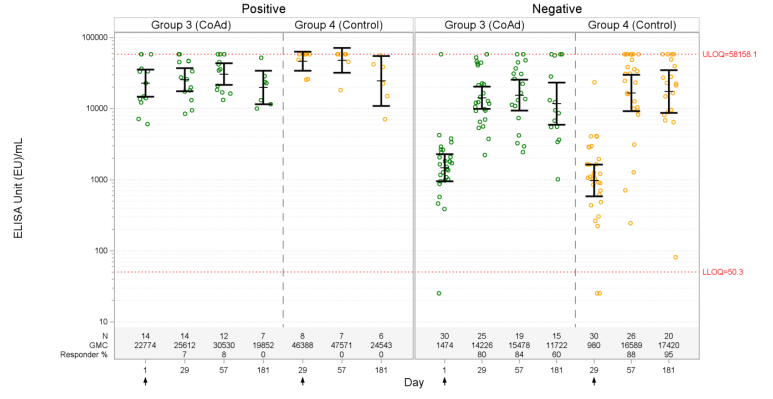

Supplement: Appendix and Tables [file mmc1.docx]
