# Supplementary material for: Safety, reactogenicity, and immunogenicity of Ad26.COV2.S co-administered with a quadrivalent standard-dose or high-dose seasonal influenza vaccine: a non-inferiority randomised controlled trial
Source: eClinicalMedicine. 2025 Jan 7;79:103016. doi: 10.1016/j.eclinm.2024.103016 (PMC11764035; doi:10.1016/j.eclinm.2024.103016)
Supplement: Redacted_Protocol-Amend [file mmc2.pdf]

Janssen Vaccines & Prevention B.V.*

Clinical Protocol

A Randomized, Double-blind, Phase 3 Study to Evaluate Safety, Reactogenicity, and Immunogenicity of Co-administration of Ad26.COV2.S and Influenza Vaccines in Healthy Adults 18 Years of Age and Older

Protocol VAC31518COV3005; Phase 3
Version: Amendment 1

VAC31518 (JNJ-78436735)

* Janssen Vaccines & Prevention B.V. is a Janssen pharmaceutical company of Johnson & Johnson and is hereafter referred to as the sponsor of the study. The sponsor is identified on the Contact Information page that accompanies the protocol.

United States (US) sites of this study will be conducted under US Food & Drug Administration Investigational New Drug (IND) regulations (21 CFR Part 312).

Regulatory Agency Identifier Number(s):

IND: 22657
EudraCT NUMBER: 2021-003953-43

**Status:**	Approved
**Date:**	15 February 2022
**Prepared by:**	Janssen Vaccines & Prevention B.V.
**EDMS number:**	EDMS-RIM-453567, 2.0

GCP Compliance: This study will be conducted in compliance with Good Clinical Practice, and applicable regulatory requirements.

Confidentiality Statement
The information provided herein contains Company trade secrets, commercial or financial information that the Company customarily holds close and treats as confidential. The information is being provided under the assurance that the recipient will maintain the confidentiality of the information under applicable statutes, regulations, rules, protective orders or otherwise.

PROTOCOL AMENDMENT SUMMARY OF CHANGES TABLE

DOCUMENT HISTORY	
**Document**	**Date**
Amendment 1	This document
Original Protocol	20 July 2021

Amendment 1 (This document)

Overall Rationale for the Amendment:

The study has encountered unexpected barriers and limitations in the recruitment of participants. In view of the challenges, the non-inferiority assessment will be limited to the groups receiving the standard dose only (Groups 1 and 2). The non-inferiority assessment of the *high-dose* (Groups 3 and 4) will be evaluated as a secondary endpoint due to a decrease in the sample size and a descriptive analysis will be performed instead.

As the study considered a timeframe of 6 months since the primary COVID-19 vaccination, the global availability of COVID-19 booster doses along with recommendations from advisory committees to administer booster vaccinations has decreased the projected pool of participants eligible for the study.

It has become more difficult to find eligible candidates to populate the *high-dose* groups due to early influenza campaigns prioritizing vaccination for older adults.

The overall sample size and objectives for the study have been revised to reflect the changes described above.

Section Number and Name	Description of Change	Brief Rationale
1.1 Synopsis<br>3 OBJECTIVES AND ENDPOINTS<br>4.1 Overall Design<br>9.1 Statistical Hypotheses<br>9.2 Sample Size Determination<br>9.4.2 Inferential Endpoints<br>9.4.3 Other Secondary Endpoints	The non-inferiority objectives for the high-dose influenza vaccine were moved from Primary to Secondary, with data for the high-dose vaccine to be presented descriptively.<br><br>2-sided alpha for the non-inferiority (NI) analysis was modified to 5%, the CIs were adjusted to 95%, and the sample size was modified accordingly.	It is unlikely that enough participants receiving the high-dose influenza vaccine will be recruited to power the assessment for the primary analysis.<br><br>The NI assessment will be limited to the standard-dose groups.

Section Number and Name	Description of Change	Brief Rationale
1.1 Synopsis<br>3 OBJECTIVES AND ENDPOINTS	Text was added to the primary objectives to specify the assay and NI margins.	Clarification
	An exploratory objective was added to assess the correlation between the binding antibody (ELISA) titers and neutralizing antibody (VNA) titers to SARS-CoV-2, in a subset of participants at selected timepoints.	Consistency with other COVID-19 program studies
	Two exploratory objectives were added:<br><br>1. To assess humoral responses to Ad26.COV2.S and influenza vaccine based on the previous history of COVID-19 vaccination at entry.<br><br>2. To explore the humoral responses to Ad26.COV2.S and influenza vaccine based on the SARS-CoV-2 serostatus at baseline (N-serology).	At the request of health authorities
1.1 Synopsis<br>4.1 Overall Design<br>9.2 Sample Size Determination	The cap on 60% of participants ≤64 years of age was replaced with language that allows for greater flexibility in the enrollment of patients ≤64 years of age, and sample sizes were adjusted accordingly.	Difficulty in recruitment of participants 65 years of age and older due to new COVID-19 booster recommendations and influenza vaccine campaigns will compromise the feasibility and will not allow study endpoints to be met.
5.1 Inclusion Criteria	Inclusion criterion #5 was modified to include wording about the “last” COVID-19 vaccination.	Most potential study participants have now received at least one booster vaccination.
	Inclusion criterion #6 was modified to reduce the timeframe in which medication must remain stable prior to study vaccination.	The target population is healthy individuals or those with stable comorbidities. For individuals with chronic comorbidities is not infrequent that physicians make dose changes to adjust the adequate dose level regimen. Therefore, only recent dose changes within the past 4 weeks will be exclusionary.
	Inclusion criterion #8 was modified to indicate that pregnancy test should occur prior to vaccination.	Clarification

Section Number and Name	Description of Change	Brief Rationale
5.2 Exclusion Criteria	Exclusion criterion #2 was modified from a history of malignancy within 5 years before screening to a history of malignancy within 1 year before screening.	To align with the Phase 3 studies in the program.
	Wording for Exclusion Criterion #5 was modified to indicate the definition of immunosuppressive dose as stated per (>20 mg prednisone or equivalent daily for 2 consecutive weeks). Intraarticular steroids have been removed from this sentence.	Intraarticular steroids are typically use as a single shot indicated as long-term pain and inflammation reliever. The criteria defines an immunosuppressive steroid dose is considered to be >20 mg prednisone or equivalent daily for 2 consecutive weeks.<br>That definition is not consistent with the typical frequency of use in clinical practice.
	Exclusion criterion #7 was deleted.	Eczema is a common side effect of medication and not always an indication of an autoimmune disease; therefore, clinical judgment is required to establish the difference. These conditions are covered under exclusion criterion #5.
	Exclusion criterion #12 was modified to indicate that planning to receive a licensed/registered SARS-CoV-2 vaccine less than 6 months prior to first study vaccination is not exclusionary.	Only having actually received a vaccine less than 6 months prior or during the study would exclude an individual from participating in this study.
	Exclusion criterion #24 was modified to indicate that diagnostic test result should be obtained prior to vaccination.	Clarification
2.3 Benefit-Risk Assessment<br>2.3.3 Benefit-Risk Assessment of Study Participation<br>8.2 Safety Assessments	Text regarding reporting of clinically significant abnormalities was modified.	Clarification
6.1 Study Vaccines Administered	Text was modified regarding injection location and visit windows.	Alternative injection locations are allowed under some circumstances however, the text was modified to indicate that the participant should be able to differentiate between the 2 injections in order to accurately report local reactogenicity symptoms. Vaccination visit windows are critical for the study endpoints therefore the sponsor encourages communication from investigators when necessary.

Section Number and Name	Description of Change	Brief Rationale
6.1.1 Combination Products	Section was added because influenza vaccines used in this study are considered drug/device combinations.	To provide guidance on combination products
6.8 Concomitant Therapy	The sentence regarding history of SARS-CoV-2 vaccination has been deleted, and a statement was added that in the event that a participant receives a COVID-19 vaccine outside of the study, it must be recorded as a concomitant medication.<br><br>The text regarding antipyretic use was modified to include "per the investigator's discretion."<br><br>The text regarding systemic corticosteroids was updated to include the definition of immunosuppressive dose, to replace "within 6 months before planned administration of the first dose of study vaccine" to "treatment phase," and to replace "must take priority over the study vaccine" with "medical judgment should prevail."	The history of COVID-19 vaccines is not listed as concomitant therapy but as history of SARS-CoV-2 vaccination.<br><br>To complete the sentence<br><br>Use of an immunosuppressive dose of steroids at study entry is exclusionary. If it becomes necessary due to standard of care during the treatment phase, it is subject to medical judgment.
7.1 Discontinuation of Study Vaccination	A condition was added for influenza vaccine (other than the one administered in the study) for the current influenza season in the Northern Hemisphere.	To avoid confounding the study endpoints
8 STUDY ASSESSMENTS AND PROCEDURES	Physical examination was added to the paragraph regarding the preferred sequence for multiple assessments.<br><br>Investigator assessment of reactogenicity was added to the paragraph regarding participant diary completion.	To correct prior omissions<br><br>To clarify the timepoint for reactogenicity data collection
2.3.3 Benefit-Risk Assessment of Study Participation<br>8.2.3 Clinical Safety Laboratory Assessments<br>8.3.1 Time Period and Frequency for Collecting Adverse Event, Medically-attended Adverse Event, Adverse Event of Special Interest, and Serious Adverse Event Information<br>8.3.6.1 Thrombosis with Thrombocytopenia Syndrome	Text was clarified regarding procedures for laboratory testing in the event of post-vaccination thrombocytopenia.<br><br>"Post vaccination" was added to thrombocytopenia.	For consistency of reporting requirements of suspected AESI

Section Number and Name	Description of Change	Brief Rationale
1.3.1 All Participants<br>8.3.1 Time Period and Frequency for Collecting Adverse Event, Medically-attended Adverse Event, Adverse Event of Special Interest, and Serious Adverse Event Information	A statement was added that previous SARS-CoV-2 infection should be documented as medical history.<br><br>A statement was added that AESIs occurring during the study period must be reported to the sponsor by study site personnel within 24 hours of their knowledge of the event	To be able to capture previous SARS-COV-2 infections
8.3.2 Method of Detecting Adverse Events, Medically-attended Adverse Events, Adverse Events of Special Interest, and Serious Adverse Events<br>11 REFERENCES	Cross-references to Appendix 6 were added for solicited AEs; reference to Marcy 2004 was removed.	To maintain consistent toxicity grading criteria
1.3.1 All Participants<br>1.3.2 Participants with a Suspected AESI<br>8.3.6 Adverse Events of Special Interest	Anti-PF4 was deleted from the third paragraph of Section 8.3.6 and the first sentence in footnote k of the Schedule of Activities for All Participants, and the first sentence of footnote d in the Schedule of Activities for Participants with a Suspected AESI.	Anti-PF4 is not part of a complete blood count.
1.3.1 All Participants<br>1.3.2 Participants with a Suspected AESI<br>8.3.6.1 Thrombosis with Thrombocytopenia Syndrome<br>10.2 Appendix 2: Clinical Laboratory Tests	Anti-PF4 testing was changed from local to central laboratory testing.	Anti-PF4 is a supportive parameter for the investigation of a suspected AESIs. The sponsor will test those samples centrally, as it is not always feasible locally.
1.1 Synopsis<br>4.1 Overall Design<br>6.3 Measures to Minimize Bias: Randomization and Blinding<br>9.3 Populations for Analysis Sets<br>9.4.3 Other Secondary Endpoints	Text was added to describe the handling of vaccine-naïve participants in the analysis.	At the request of health authorities
6.7 Treatment of Overdose	Text was added that in the event of an overdose, the investigator could contact the sponsor or Medical Monitor.	Clarification

Section Number and Name	Description of Change	Brief Rationale
9.3 Populations for Analysis Sets	PPII population: "Ad26.COV2.S" was corrected to "seasonal influenza vaccine" for the control group.<br><br>PPSI population: Text was added to indicate that samples obtained from participants after natural SARS-CoV-2 infection or after vaccination with another COVID-19 vaccine will be excluded from the per protocol analysis.	Corrections
9.4.2 Inferential Endpoints	Definition of baseline was added.	To align with SAP
9.4.6 Other Analyses	Text was modified to indicate that site personnel and participants will be blinded during the treatment phase, rather than until the end of the study.	Once participants have received both study vaccines, they can be unblinded. For practical reasons, study participants need to know whether they received Ad26.COV2.S on Day 1 or Day 29, first, to enable them to count a number of months to receive a booster vaccination and second, to receive certification in some countries, HCPs must complete a form with the vaccination date and manufacturer.
Throughout the protocol	Minor grammatical, formatting, or spelling changes were made.	Minor errors were noted.

TABLE OF CONTENTS

PROTOCOL AMENDMENT SUMMARY OF CHANGES TABLE 2

TABLE OF CONTENTS 8

LIST OF IN-TEXT TABLES AND FIGURES 10

1. PROTOCOL SUMMARY 11
1.1. Synopsis 11
1.2. Schema 19
1.3. Schedule of Activities 20
1.3.1. All Participants 20
1.3.2. Participants with a Suspected AESI 23

2. INTRODUCTION 25
2.1. Study Rationale 27
2.2. Background 28
2.3. Benefit-Risk Assessment 32
2.3.1 Risks Related to Study Participation 32
2.3.2 Benefits of Study Participation 37
2.3.3 Benefit-Risk Assessment of Study Participation 37

3. OBJECTIVES AND ENDPOINTS 39

4. STUDY DESIGN 43
4.1. Overall Design 43
4.2. Scientific Rationale for Study Design 45
4.2.1. Study-Specific Ethical Design Considerations 45
4.3. Justification for Dose 46
4.4. End of Study Definition 46

5. STUDY POPULATION 46
5.1. Inclusion Criteria 47
5.2. Exclusion Criteria 49
5.3. Lifestyle Considerations 52
5.4. Screen Failures 52
5.5. Criteria for Temporarily Delaying Administration of Study Vaccine 52

6. STUDY VACCINATION AND CONCOMITANT THERAPY 53
6.1. Study Vaccines Administered 53
6.1.1. Combination Products 56
6.2. Preparation/Handling/Storage/Accountability 56
6.3. Measures to Minimize Bias: Randomization and Blinding 57
6.4. Study Vaccination Compliance 58
6.5. Dose Modification 58
6.6. Access to Licensed/Authorized Study Vaccine After the End of the Study 58
6.7. Treatment of Overdose 59
6.8. Concomitant Therapy 59

7. DISCONTINUATION OF STUDY VACCINATION AND PARTICIPANT DISCONTINUATION/WITHDRAWAL 61
7.1. Discontinuation of Study Vaccination 61
7.2. Participant Discontinuation/Withdrawal From the Study 61
7.2.1. Withdrawal From the Use of Research Samples 62
7.3. Lost to Follow-up 62

8. STUDY ASSESSMENTS AND PROCEDURES 63
8.1. Immunogenicity Assessments 65

8.2. Safety Assessments ... 66
8.2.1. Physical Examinations ... 66
8.2.2. Vital Signs ... 66
8.2.3. Clinical Safety Laboratory Assessments ... 67
8.2.4. Pregnancy Testing ... 67
8.3. Adverse Events, Serious Adverse Events, Medically-attended Adverse Events, Adverse Events of Special Interest, and Other Safety Reporting ... 67
8.3.1. Time Period and Frequency for Collecting Adverse Event, Medically-attended Adverse Event, Adverse Event of Special Interest, and Serious Adverse Event Information ... 68
8.3.2. Method of Detecting Adverse Events, Medically-attended Adverse Events, Adverse Events of Special Interest, and Serious Adverse Events ... 69
8.3.3. Follow-up of Adverse Events, Medically-attended Adverse Events, Adverse Events of Special Interest, and Serious Adverse Events ... 71
8.3.4. Regulatory Reporting Requirements for Serious Adverse Events ... 71
8.3.5. Pregnancy ... 71
8.3.6. Adverse Events of Special Interest ... 72
8.3.6.1. Thrombosis with Thrombocytopenia Syndrome ... 72

9. STATISTICAL CONSIDERATIONS ... 73
9.1. Statistical Hypotheses ... 73
9.2. Sample Size Determination ... 74
9.3. Populations for Analysis Sets ... 75
9.4. Statistical Analyses ... 76
9.4.1. General Considerations ... 76
9.4.2. Inferential Endpoints ... 76
9.4.3. Other Secondary Endpoints ... 77
9.4.4. Exploratory Endpoints ... 78
9.4.5. Safety Analyses ... 78
9.4.6. Other Analyses ... 79
9.5. Interim Analysis ... 79

10. SUPPORTING DOCUMENTATION AND OPERATIONAL CONSIDERATIONS ... 80
10.1. Appendix 1: Abbreviations and Definitions ... 80
10.2. Appendix 2: Clinical Laboratory Tests ... 82
10.3. Appendix 3: Regulatory, Ethical, and Study Oversight Considerations ... 83
10.3.1. Regulatory and Ethical Considerations ... 83
10.3.2. Financial Disclosure ... 86
10.3.3. Informed Consent Process ... 86
10.3.4. Data Protection ... 87
10.3.5. Long-Term Retention of Samples for Additional Future Research ... 88
10.3.6. Committees Structure ... 88
10.3.7. Publication Policy/Dissemination of Clinical Study Data ... 88
10.3.8. Data Quality Assurance ... 89
10.3.9. Case Report Form Completion ... 90
10.3.10. Source Documents ... 90
10.3.11. Monitoring ... 91
10.3.12. On-Site Audits ... 92
10.3.13. Record Retention ... 92
10.3.14. Study and Site Start and Closure ... 92
10.4. Appendix 4: Adverse Events, Serious Adverse Events, Adverse Events of Special Interest, Product Quality Complaints, and Other Safety Reporting: Definitions and Procedures for Recording, Evaluating, Follow-up, and Reporting ... 94
10.4.1. Adverse Event Definitions and Classifications ... 94
10.4.2. Attribution Definitions ... 95
10.4.3. Severity Criteria ... 95
10.4.4. Special Reporting Situations ... 96
10.4.5. Procedures ... 96
10.4.6. Product Quality Complaint Handling ... 98

10.4.7. Contacting Sponsor Regarding Safety, Including Product Quality 98
10.5. Appendix 5: Contraceptive Guidance and Collection of Pregnancy Information 99
10.6. Appendix 6: Toxicity Grading Scale 100
10.7. Appendix 7: Summary of Guidance from CDC Website on Underlying Medical Conditions That Lead or Might Lead to Increased Risk for Severe Illness From COVID-19 104
10.8. Appendix 8: TTS AESI Form 105
10.9. Appendix 9: Thrombotic Events to be Reported as AESIs 109
10.10. Appendix 10: Protocol Amendment History 110

11. REFERENCES 111

INVESTIGATOR AGREEMENT 115

LIST OF IN-TEXT TABLES AND FIGURES

TABLES

Table 1: Adverse Drug Reactions Associated With Ad26.COV2.S 32
Table 2: Overview of the Groups and Vaccination Days 43
Table 3: Description of Interventions 55
Table 4: Summary of Humoral Immunogenicity Assays 65

FIGURES

Figure 1: Schematic Overview of the Study 19

1. PROTOCOL SUMMARY

1.1. Synopsis

A Randomized, Double-blind, Phase 3 Study to Evaluate Safety, Reactogenicity, and Immunogenicity of Co-administration of Ad26.COV2.S and Influenza Vaccines in Healthy Adults 18 Years of Age and Older.

Ad26.COV2.S (also known as VAC31518, JNJ-78436735) is a monovalent vaccine composed of a recombinant, replication-incompetent adenovirus type 26 (Ad26) vector, constructed to encode the Spike (S) protein derived from a SARS-CoV-2 clinical isolate (Wuhan, 2019, whole-genome sequence NC 045512, referred to as the original strain), stabilized in its prefusion conformation.

The study aims to assess the safety, reactogenicity, and immunogenicity of the Ad26.COV2.S vaccine co-administered with a quadrivalent *standard-dose* or *high-dose* seasonal influenza vaccine compared to administration of each vaccine separately to explore whether Ad26.COV2.S and the influenza vaccines can be administered concomitantly.

The primary objective (for the NI assessment) will be evaluated in approximately 610 participants aged 18 and above who have completed a primary COVID-19 vaccine series, the co-administration of the Ad26.COV2.S and the seasonal quadrivalent *standard-dose* influenza vaccine. In a cohort of participants aged 65 and above, the co-administration of the Ad26.COV2.S and the seasonal quadrivalent *high-dose* influenza vaccine will be assessed descriptively. In another cohort of COVID-19 vaccine-naïve individuals, immune responses will be evaluated descriptively.

OBJECTIVES AND ENDPOINTS

Objectives	Endpoints
**Primary**	
To demonstrate the non-inferiority (NI) of the humoral immune response of the 4 influenza vaccine strains after concomitant administration of the Ad26.COV2.S vaccine and a seasonal quadrivalent *standard-dose* influenza vaccine versus the administration of a seasonal quadrivalent *standard-dose* influenza vaccine administered alone.	• Antibody hemagglutination inhibition (HI) titers as measured by hemagglutinin inhibition (HAI) assay titers (geometric mean titers [GMTs]) against each of the 4 influenza vaccine strains, 28 days after the administration of a seasonal quadrivalent *standard-dose* influenza vaccine<br>*Success criteria for NI*<br>• the upper bound of the 2-sided 95% confidence interval (CI) for the geometric mean titer (GMT) ratio (Control group/co-administration [CoAd] group) lies below 1.5
To demonstrate the NI of the binding antibody response after concomitant administration of Ad26.COV2.S vaccine and a seasonal quadrivalent *standard-dose* influenza vaccine versus the administration of Ad26.COV2.S vaccine administered alone.	• Antibody titers as measured by S enzyme-linked immunosorbent assay (S-ELISA) titers (GMC), 28 days after administration of Ad26.COV2.S vaccine.<br>*Success criteria for NI*<br>• the upper bound of the 2-sided 95% confident interval (CI) for the GMC ratio (Control group/CoAd group) lies below 1.5

Objectives	Endpoints
**Secondary**	
To assess the safety and reactogenicity of a single dose of Ad26.COV2.S vaccine when administered separately or concomitantly with a seasonal quadrivalent *high-dose* influenza vaccine in participants aged 65 years and older.	• Solicited local (injection site) and systemic adverse events (AEs) for 7 days after each vaccination.<br>• Unsolicited adverse events (AEs) for 28 days after each vaccination.<br>• Serious adverse events (SAEs), medically attended adverse events (MAAEs), and adverse events of special interest (AESIs) throughout the study.<br>• AEs leading to withdrawal from the study throughout the study.
To assess the safety and reactogenicity of a single dose of Ad26.COV2.S vaccine when administered separately or concomitantly with a seasonal quadrivalent *standard-dose* influenza vaccine in participants aged 18 years and older.	• Solicited local (injection site) and systemic AEs for 7 days after each vaccination.<br>• Unsolicited AEs for 28 days after each vaccination.<br>• SAEs, MAAEs, and AESIs throughout the study.<br>• AEs leading to withdrawal from the study throughout the study.
To assess the humoral immune response against each of the 4 influenza vaccine strains after concomitant administration of the Ad26.COV2.S vaccine and a seasonal quadrivalent *high-dose* influenza vaccine versus the administration of a seasonal quadrivalent *high-dose* influenza vaccine administered alone.	• Antibody HI titers as measured by HAI assay titers (GMTs) against each of the 4 influenza vaccine strains, 28 days after the administration of a seasonal quadrivalent *standard-dose* influenza vaccine.
To assess the humoral immune response of Ad26.COV2.S vaccine and a seasonal quadrivalent *high-dose* standard-dose influenza vaccine versus the administration of Ad26.COV2.S vaccine administered alone.	• Antibody titers as measured by S enzyme-linked immunosorbent assay (S-ELISA) titers (GMC), 28 days after administration of Ad26.COV2.S vaccine.
To assess the humoral response to SARS-CoV-2 after the concomitant administration of Ad26.COV2.S vaccine and a seasonal quadrivalent *high-dose* influenza vaccine versus the administration of Ad26.COV2.S vaccine alone *in COVID-19 vaccine-naïve individuals*.	• Antibody titers as measured by S enzyme-linked immunosorbent assay (S-ELISA) titers (GMC), 28 days after administration of Ad26.COV2.S vaccine.
To assess the humoral response to SARS-CoV-2 after the concomitant administration of Ad26.COV2.S vaccine and a seasonal quadrivalent *standard-dose* influenza vaccine versus the administration of Ad26.COV2.S vaccine alone *in COVID-19 vaccine-naïve individuals*	• Antibody titers as measured by S enzyme-linked immunosorbent assay (S-ELISA) titers (GMC), 28 days after administration of Ad26.COV2.S vaccine.

Objectives	Endpoints
To compare seroconversion rates against the 4 influenza vaccine strains after the concomitant administration of Ad26.COV2.S vaccine and a seasonal quadrivalent (*high-dose* and *standard-dose*) influenza vaccine versus a vaccination of a seasonal quadrivalent (*high-dose* and *standard-dose*) influenza vaccine administered alone.	• Seroconversion is defined for each of the 4 influenza vaccine strains at 28 days after the administration of a seasonal quadrivalent (*high-dose* and *standard-dose*) influenza vaccine:<br>HI titer ≥1:40 in participants with a pre-vaccination HI titer of <1:10, or<br>a ≥4-fold HI titer increase in participants with a pre-vaccination HI titer of ≥1:10.
To assess seroprotection rates against the 4 influenza vaccine strains after the concomitant administration of Ad26.COV2.S vaccine and a seasonal quadrivalent (*high-dose* and *standard-dose*) influenza vaccine versus a vaccination of a seasonal quadrivalent (*high-dose* and *standard-dose*) influenza vaccine administered alone.	• Seroprotection is defined for each of the 4 influenza vaccine strains as HI titer ≥1:40 at 28 days after the administration of a seasonal quadrivalent (*high-dose* and *standard-dose*) influenza vaccine.
**Exploratory**	
To evaluate the durability of influenza-specific humoral immune responses after concomitant administration of the vaccines versus the influenza vaccine alone.	• GMT: Geometric mean of HI antibodies at Day 181.<br>• Percentages of participants with HI titers ≥1:40 on Day 181.
To evaluate the durability of SARS-CoV-2 specific humoral immune responses after concomitant administration of the vaccines versus the SARS-CoV-2 vaccine alone.	• Antibody GMC by S-ELISA at Day 181 after the administration of Ad26.COV2.S vaccine.
To assess the magnitude and durability of humoral immune responses specific to influenza.	• Serological responses to vaccination as measured by HAI at Day 181 after the administration of influenza vaccine.
To further assess humoral responses to Ad26.COV2.	• Serological response to vaccination, as measured by virus neutralization assay (VNA) (SARS-CoV-2 VNA and/or pseudovirion [ps] VNA expressing S protein) titers.<br>• Adenovirus 26 neutralization responses measured by VNA.<br>• Functional and molecular antibody characterization including Fc-mediated viral clearance, avidity, Fc characteristics, immunoglobulin (Ig) subclass, and IgG isotype.<br>• Correlation between ELISA (S-ELISA) and VNA (wild-type virus [wt]VNA and/or pseudovirion [ps]VNA) titers at selected timepoints.

Objectives	Endpoints
To explore the humoral responses to Ad26.COV2.S and influenza vaccine based on the SARS-CoV-2 serostatus at baseline (N-serology).	• Serological response to vaccination as measured by S-ELISA antibody concentration 28 days after the administration of Ad26.COV2.S vaccine.<br>• Antibody GMC by S-ELISA at 28 days after the administration of Ad26.COV2.S vaccine.<br>• Antibody HI titers as measured by HAI assay titers (GMTs) against the 4 influenza strains.
To explore the humoral responses to Ad26.COV2.S and influenza vaccine based on the previous history of COVID-19 vaccination at entry (viral vector vaccine or mRNA vaccine or vaccine-naïve).	• Serological response to vaccination as measured by S-ELISA antibody concentration 28 days after the administration of Ad26.COV2.S vaccine.<br>• Antibody GMC by S-ELISA at 28 days after the administration of Ad26.COV2.S vaccine.<br>• Antibody HI titers as measured by HAI assay titers (GMTs) against SARS-CoV-2.

Hypotheses

The primary hypotheses of this study are:

The concomitant administration of the Ad26.COV2.S vaccine and a seasonal quadrivalent influenza vaccine (*standard-dose*) is non-inferior than the administration of the seasonal quadrivalent influenza vaccine (*standard-dose*) alone as measured by HI titers against each of the 4 influenza vaccine strains at 28 days after the administration of a quadrivalent seasonal influenza vaccine,

AND

The concomitant administration of the Ad26.COV2.S vaccine and a seasonal quadrivalent influenza vaccine (*standard-dose*) is non-inferior than the administration of the Ad26.COV2.S vaccine alone as measured by S-ELISA antibody titers at 28 days after the administration of the Ad26.COV2.S vaccine.

For the high-dose groups, no formal hypothesis testing is planned.

No formal statistical testing of safety data is planned. Safety data will be analyzed descriptively by vaccine group.

OVERALL DESIGN

This is a randomized, double-blind, parallel, multicenter, interventional study in healthy (including stable comorbidities) adults ≥18 years of age. In this study, the safety, reactogenicity, and immunogenicity of Ad26.COV2.S co-administered with an influenza vaccine will be evaluated.

Approximately 1,100 participants will be randomized in parallel in this study. Participants aged ≥65 will be randomized in a 1:1:1:1 ratio to 1 of 4 groups. Participants aged ≤64 will be randomized in a 1:1 ratio to 1 of 2 groups (Group 1 or Group 2). Efforts will be made to ensure good representation in terms of race, gender, and ethnicity.

Participants will receive Ad26.COV2.S and a seasonal quadrivalent (*standard-dose* or *high-dose*) influenza vaccine either concomitantly on Day 1 and placebo on Day 29 (co-administration groups 1 and 3) or a seasonal quadrivalent (*standard-dose* or *high-dose*) influenza vaccine and placebo on Day 1 and Ad26.COV2.S on Day 29 (control groups 2 and 4).

An overview of the groups and vaccinations is given in the table below.

Table: Overview of the Groups and Vaccinations

Group	Day 1	Day 29
1 (CoAd group)	Ad26.COV2.S + Q SD influenza vaccine	Placebo
2 (Control group)	Placebo + Q SD influenza vaccine	Ad26.COV2.S
3 (CoAd group)	Ad26.COV2.S + Q HD influenza vaccine	Placebo
4 (Control group)	Placebo + Q HD influenza vaccine	Ad26.COV2.S

CoAd = co-administration; HD = high-dose; Q = quadrivalent; SD = standard-dose.

The seasonal quadrivalent *standard-dose* influenza vaccine (Groups 1 and 2) can be administered to all participants. Age is included as stratification factor for Groups 1 and 2 (≥18 to ≤64 years of age and ≥65 years of age).

The seasonal quadrivalent *high-dose* influenza vaccine (Groups 3 and 4) will only be administered to participants ≥65 years of age.

The previous SARS-CoV-2 vaccination history (Vaxzevria [AstraZeneca], Comirnaty [Pfizer-BioNTech], SpikeVax [Moderna], Ad26.COV2-S [Janssen]or COVID-19 vaccine naïve) will be used as second stratification factor. This stratification factor will aim to obtain a balanced distribution of SARS-CoV-2 vaccination history across the 4 treatment groups.

Participants can either have received a complete primary vaccination with an authorized/licensed COVID-19 vaccine[a] (completed ≥6 months prior to first study vaccination) or be COVID-19 vaccine-naïve. The type of primary vaccination will be documented and accounted for according to interactive web response system (IWRS) procedures.

The study duration from screening until the last follow-up visit will be approximately 7-8 months. The study comprises screening on Day -28 to 1, vaccination visits on Days 1 and 29 with a 28-day follow-up period after each vaccination. AEs leading to withdrawal, MAAEs, SAEs, and AESIs will be collected throughout the study.

End of Study Definition

The end of the study is considered as the last visit for the last participant in the study.

NUMBER OF PARTICIPANTS

Overall, approximately 1,100 adult participants aged ≥18 years will be enrolled in the study.

[a] The following authorized/licensed COVID-19 vaccines are permitted in this study: Vaxzevria (AstraZeneca), Comirnaty (Pfizer-BioNTech), SpikeVax (Moderna), and Ad26.COV2.S (Janssen).

DOSAGE AND ADMINISTRATION

Ad26.COV2.S will be supplied at a concentration of 1×10^{11} vp/mL in single-use vials, with an extractable volume of 0.5 mL, and dosed at 5×10^{10} vp. Placebo (0.9% NaCl) will be supplied in single-use vials.

The seasonal influenza vaccines to be used in this study are Afluria Quadrivalent (*standard-dose*) and Fluzone HD Quadrivalent (*high-dose*) or equivalent formulated for the 2021-2022 influenza season:

- Afluria Quadrivalent (*standard-dose*) is a suspension for intramuscular (IM) injection supplied in a 0.5mL single-dose prefilled syringe. Each dose of Afluria Quadrivalent contains 60 µg of hemagglutinin (HA): 15 µg of each of 4 influenza strains (2 influenza A strains and 2 influenza B strains). For details, refer to the Afluria Quadrivalent package insert.
- Fluzone HD Quadrivalent (*high-dose*) is a suspension for IM injection supplied in a 0.7mL single-dose prefilled syringe. Each dose of Fluzone HD Quadrivalent contains 240 µg of HA: 60 µg of each of 4 influenza strains (2 influenza A strains and 2 influenza B strains). For details, refer to the Fluzone HD Quadrivalent package insert.

Study vaccines will be administered by IM injection into the deltoid muscle. On Day 1, each participant will receive 2 IM injections. Each injection should be administered in opposite arms. It is recommended to use the right arm for the seasonal influenza vaccine on Day 1 and the left arm for the Ad26.COV2.S vaccine or placebo on Days 1 and 29.

On Day 29, each participant will receive 1 IM injection, preferably into the deltoid muscle of the non-dominant upper arm.

If an injection cannot be given in the deltoids due to a medical or other contraindication (for example, tattooed upper arms rendering it difficult to assess site reactogenicity), use alternative locations such as the hip, thigh, or buttocks (to be avoided in overweight participants). When selecting an alternative location, the participant's ability to assess injection site events should be considered.

In all circumstances, IM injections in other locations than the upper arm are not considered protocol deviations.

IMMUNOGENICITY EVALUATIONS

Venous blood samples will be collected for assessment of humoral immune responses. Sample volumes and time points are detailed in the Schedule of Activities. Humoral immunogenicity assays may include, but are not limited to, the assays in the table below.

Table: Summary of Humoral Immunogenicity Assays

Assay	Purpose
Primary endpoints	
HI assay[a]	Analysis of HI to influenza strains
SARS-CoV-2 binding antibodies (ELISA)	Analysis of antibodies binding to SARS-CoV-2 or individual SARS-CoV-2 proteins (eg, S protein)
Secondary endpoints	
HI assay [a]	Analysis of HI to influenza strains
SARS-CoV-2 binding antibodies (ELISA)	Analysis of antibodies binding to SARS-CoV-2 or individual SARS-CoV-2 proteins (eg, S protein)
Exploratory endpoints	
HI assay	Analysis of HI to influenza strains
SARS-CoV-2 binding antibodies (ELISA)	Analysis of antibodies binding to SARS-CoV-2 or individual SARS-CoV-2 proteins (eg, S protein)

Assay	Purpose
Primary endpoints	
SARS-CoV-2 neutralization (VNA)	Analysis of neutralizing antibodies to the wild-type virus and/or pseudovirion expressing S protein
Adenovirus neutralization	Adenovirus 26 neutralization assay to evaluate neutralizing antibody responses against the Ad26 vector
SARS-CoV-2 binding antibodies (ELISA and/or SARS-CoV-2 immunoglobulin assay)	Analysis of antibodies binding to the SARS-CoV-2 N protein
Functional and molecular antibody characterization	Analysis of antibody characteristics including Fc-mediated viral clearance, avidity, Fc characteristics, Ig subclass and IgG isotype

ELISA = enzyme-linked immunosorbent assay; Fc = Fragment crystallizable; HI assay = Hemagglutination inhibition (HI) assay; Ig = immunoglobulin; S = Spike; SARS-CoV2 = severe acute respiratory syndrome coronavirus-2; VNA = virus neutralization assay.

a. The HI antibody titers against influenza virus strains recommended by the World Health Organization (WHO) for use in 2021-2022 in the Northern Hemisphere season will be measured using a validated HI assay platform.

SAFETY EVALUATIONS

After each vaccination, participants will remain under observation at the study site for at least 15 minutes for presence of any acute reactions and solicited events. Participants will be asked to note in the diary occurrences of local reactions at injection site namely pain/tenderness, erythema, swelling, and systemic reactions namely fatigue, headache, nausea, and myalgia daily for 7 days post-vaccination (or until resolution).

Participants will be instructed on how to record daily temperature and local reactions using a thermometer and a ruler provided for home use. Participants should record the temperature in the diary starting in the evening of the day of vaccination, and then daily for the next 7 days approximately at the same time each day. If more than one measurement is made on any given day, the highest temperature of that day should be recorded.

Adverse events and special reporting situations, whether serious or non-serious, that are related to study procedures or that are related to non-investigational sponsor products will be reported from the time a signed and dated informed consent form (ICF) is obtained until the end of the study/early withdrawal.

Thrombosis with thrombocytopenia syndrome is considered to be an adverse event of special interest (AESI). Suspected AESIs (thrombotic events and thrombocytopenia [defined as platelet count below 150,000/µL]) will be monitored from the moment of vaccination until the end of the study/early withdrawal. An AESI Adjudication Committee with appropriate expertise will be established to evaluate each suspected AESI and determine whether it is a case of thrombosis with thrombocytopenia syndrome (TTS).

All unsolicited AEs will be reported for each vaccination from the time of vaccination until 28 days post-vaccination. All SAEs, AESIs, and AEs leading to discontinuation from the study/vaccination (regardless of the causal relationship) are to be reported from the moment of first vaccination until the end of the study. The investigator’s assessment of ongoing AEs at the time of each participant’s last visit should be documented in the participant’s medical chart.

MAAEs are defined as AEs with medically-attended visits including hospital, emergency room, urgent care clinic, or other visits to or from medical personnel for any reason. New onset of chronic diseases will be collected as part of the MAAEs. Routine study visits will not be considered medically-attended visits. MAAEs will be reported throughout the study.

STATISTICAL METHODS

Sample size calculations are performed under the following assumptions:

- no effect of co-administration of Ad26.COV2.S vaccine and seasonal influenza vaccine on the immune response against influenza as measured by HI antibody titers against the 4 influenza vaccine strains at 28 days after the administration of seasonal influenza vaccine
- the use of Afluria Quadrivalent for *standard-dose* (adults ≥18 to <64 years of age and older adults ≥65 years of age)
- a standard deviation of 0.53 for the *standard-dose* at the $\log_{10}$ scale for HI antibody titers against the 4 influenza vaccine strains at 28 days after the administration of seasonal influenza vaccine (with or without Ad26.COV2.S)
- no effect of co-administration of Ad26.COV2.S vaccine and seasonal influenza vaccine on the immune response against SARS-CoV-2 as measured by S-ELISA at 28 days after the administration of Ad26.COV2.S vaccine
- a standard deviation of 0.50 at the $\log_{10}$ scale for S-ELISA at 28 days after the administration of Ad26.COV2.S vaccine (with or without seasonal influenza vaccine)
- a NI margin of 1.5
- 2-sided α of 5%

A total of approximately 305 (*standard-dose*) participants per group are needed to have 97.45% power to show NI in HI antibody titers for 1 influenza vaccine strain. The sample size accounts for exclusions from the per protocol set, drop-outs and missing samples resulting in a total sample size up to approximately 610 participants who have completed a primary COVID-19 vaccine series.

The overall power to show NI in HI antibody titers against each of the 4 influenza vaccine strains at 28 days after the administration of seasonal influenza vaccine as well as NI in S-ELISA at 28 days after the administration of Ad26.COV2.S vaccine is at least 90%.

Planned Analyses

The primary analysis of safety and immunogenicity will be performed when all participants have completed the 28 days after the second study vaccination visit or discontinued earlier. The primary analysis will be performed based on sponsor's unblinded data (sponsor, study-site personnel and participants will remain blinded during the treatment phase up to the database lock). No interim analyses will be performed.

A final analysis will be performed when all participants have completed the 6 month follow-up period after the second vaccination or discontinued earlier.

1.2. Schema

Figure 1: Schematic Overview of the Study

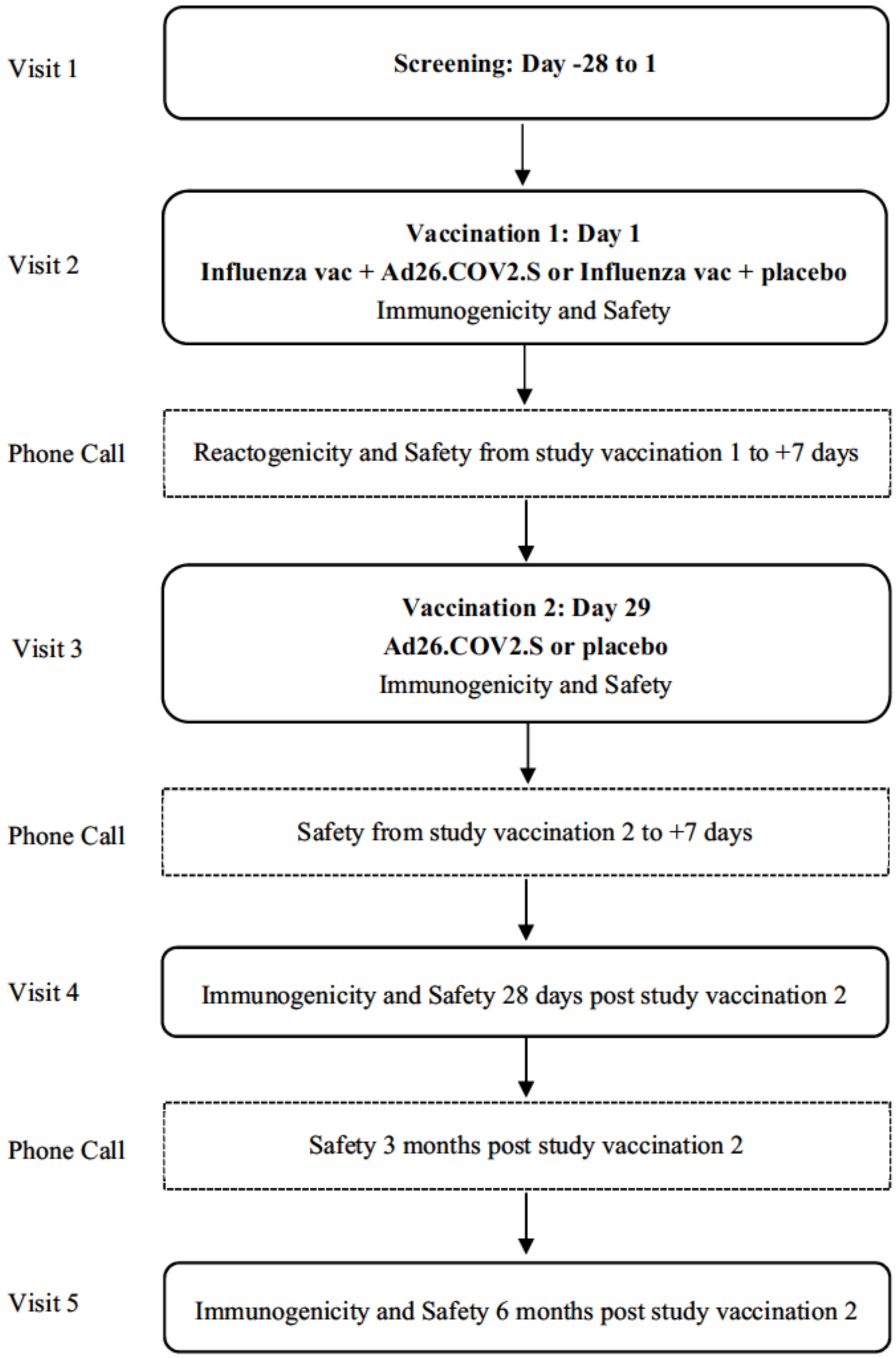

vac = vaccine

1.3. Schedule of Activities

1.3.1. All Participants

Phase	Screening[a]	Treatment Phase[b]					Follow Up[b]		Early termination[b,d]
Clinic Visit #	1	2	Phone Call[c]	3	Phone Call[c]	4	Phone Call	5	Exit[d]
Visit Timing		Vac 1	Vac 1 + 7 d	Vac 2	Vac 2 + 7 d	Post Vac 2 + 28 d	Post Vac 2 + 90 d	Post Vac 2 + 180 d	
Visit Day/Week	-28 to 1	Day 1	Day 8	Day 29	Day 36	Day 57	Week 16 (3 mo post Vac 2)	Week 28 (6 mo post Vac 2)	
Visit Window		N/A	±3 d	-3/+7 d	±3 d	-3/+7 d	±7 d	±7 d	
Visit Type	Screening	Vaccination, Immunogenicity and Safety	Safety	Vaccination, Immunogenicity and Safety	Safety	Immunogenicity and Safety	Safety	Immunogenicity and Safety	End of Study
Written informed consent[e]	●								
Inclusion/exclusion criteria	●	●[1]							
Demographics	●								
Medical history/prestudy medications	●								
History of SARS CoV 2 vaccination [f]	●								
Physical examination[g]	●								
Vital signs[h] including body temperature	●	●[2]		●[2]		●		●	●
Randomization		●[1]							
Pre vaccination check[i]		●[1]		●[1]					
Urine pregnancy test[j]	●	●[1]		●[1]					
Humoral Immunity (serum), blood draw, 15 mL		●[1]		●[1]		●		●	●[3]
Laboratory assessments: blood draw (whole blood / serum/ plasma), approx. 15 mL[k]		●[1]		●[1]					
Vaccination		●		●					
15 minutes post vaccination observation[l]		●		●					
Solicited AE recording		*From Vac 1 +7 d*		*From Vac 2 +7 d*					●[4]

Phase	Screening[a]	Treatment Phase[b]					Follow Up[b]		Early termination[b,d]
Clinic Visit #	1	2	Phone Call[c]	3	Phone Call[c]	4	Phone Call	5	Exit[d]
Visit Timing		Vac 1	Vac 1 + 7 d	Vac 2	Vac 2 + 7 d	Post Vac 2 + 28 d	Post Vac 2 + 90 d	Post Vac 2 + 180 d	
Visit Day/Week	-28 to 1	Day 1	Day 8	Day 29	Day 36	Day 57	Week 16 (3 mo post Vac 2)	Week 28 (6 mo post Vac 2)	
Visit Window		N/A	±3 d	-3/+7 d	±3 d	-3/+7 d	±7 d	±7 d	
Visit Type	Screening	Vaccination, Immunogenicity and Safety	Safety	Vaccination, Immunogenicity and Safety	Safety	Immunogenicity and Safety	Safety	Immunogenicity and Safety	End of Study
Unsolicited AE recording[m]		*- - - - - From Vac 1 through Vac 2 +28 d - - - - -*							●[5]
SAE[n] and AESI[o] recording		*- - - - - Throughout the study - - - - -*							●
AEs leading to withdrawal		*- - - - - Throughout the study - - - - -*							●
COVID 19 recording[p]		*- - - - - Throughout the study - - - - -*							●
MAAE recording[q]		*- - - - - Throughout the study - - - - -*							●
Concomitant medications[r]		*- - - - - Throughout the study - - - - -*							●
Participant diary distribution[s]		●							
Investigator's assessment of reactogenicity[t]			●		●				
Approx. blood draw per day, mL:		30		30		15		15	●[3] 15
Approx. cumulative blood draw, mL:		30		60		75		90	

●[1] pre vaccination; ●[2] pre and post vaccination; ●[3] blood samples for immunogenicity will only be taken if the early exit visit is at least 10 days after the previous immunogenicity blood draw; ●[4] if within 7 days of the vaccination; ●[5] if within 28 days of the vaccination.

The phone call or these visits (including their window) are to be scheduled relative to the actual day of the previous vaccination, indicated as Visit Timing days/weeks post vaccination.

a. Screening can be performed within 28 days prior to the first study vaccination or on the day of vaccination. If screening is performed on the day of vaccination, Visit 1 and Visit 2 will coincide on Day 1. Screening must be completed, and all eligibility criteria must be fulfilled prior to randomization and vaccination.
b. If a participant shows COVID 19 like symptoms, the participant should contact the site for guidance and must follow their local country and site level recommendations for COVID 19.
c. The site will contact the participant 7 days post vaccination 1, and 7 days post vaccination 2 to enquire about compliance and severe symptoms reported on the participant diary.

d. For those participants who are unable to continue participation in the study, but for whom consent is not withdrawn, an early exit visit could be conducted. Participants who wish to withdraw consent from participation in the study could be followed up for safety purposes if desired. This includes safety assessments of the early exit visit (no blood sampling for immunogenicity) and documenting the reason.
e. Signing of the ICF should be done before any study related activities.
f. History of SARS CoV 2 vaccination (name/manufacturer of the vaccine and date of administration) ≥6 months prior to the study will be collected in the SARS CoV 2 Vaccination page in the eCRF.
g. A full physical examination, including height and body weight, will be carried out at screening. At other clinic visits, a symptom directed physical examination can be performed in case of participant's complaints.
h. Heart rate, systolic and diastolic blood pressure, respiratory rate, and body temperature. Heart rate and blood pressure measurements should be taken in the supine position and preceded by at least 5 minutes of rest. Vital signs measurements should be performed before blood sampling. Body temperatures will be measured preferably via the oral route.
i. Investigator must check for acute illness or body temperature ≥38.0°C/100.4°F at the time of vaccination. If any of these events occur within 24 hours prior to planned vaccination, the vaccination can be rescheduled in alignment with the protocol visit windows. If the vaccination visit cannot be rescheduled within the allowed window or the contraindications to vaccination persist, the reason should be documented. The investigator should also check whether any other reasons for Discontinuation of Study Vaccination have been met.
j. For women of childbearing potential only.
k. Whole blood samples will be used for a complete blood count (CBC), including platelet count in a local laboratory or substitute for local laboratory, depending on local feasibility towards turnaround time of sample processing. Serum and plasma samples will be collected and stored for potential future coagulation related testing (including anti PF4) in a central laboratory if the participant experiences an AESI (see Section 10.2, Appendix 2).
l. Participants will be observed for a minimum of 15 minutes post vaccination or according to local regulations. Any solicited local (at injection site) and systemic AEs, unsolicited AEs, SAEs, concomitant medications, and vital signs should be documented by study site personnel. Participants will be allowed to leave the study site after the 15 minutes post vaccination observation period is completed and documented.
m. AEs and special reporting situations that are related to study procedures or vaccine products will be reported from the time a signed and dated ICF is obtained until the end of the study/early withdrawal. Unsolicited AEs should be reported from the from the time of vaccination until 28 days post vaccination.
n. All SAEs related to study procedures or vaccine products will be reported from the time a signed and dated ICF is obtained until the end of the study/early withdrawal. All other SAEs are to be reported for all participants from the moment of the first vaccination until the end of the study.
o. Suspected AESIs are to be reported from the moment of vaccination until the end of the study (see Section 8.3.1).
p. At each visit the participant will be asked if they have had a private/off study COVID 19 test. If a participant receives a positive SARS CoV 2 result from a private/off study test (whether through PCR or serological testing) the event should be reported as medical history, AE or SAE if the event meets the criteria for an SAE. The participant can continue in the study in accordance with country and site level recommendations for COVID 19.
q. MAAEs are to be reported throughout the study. New onset of chronic diseases will be collected as part of the MAAEs.
r. Concomitant therapies such as analgesic/antipyretic medications and non steroidal anti inflammatory drugs, corticosteroids, antihistamines, and vaccinations must be recorded (including dose and frequency) from the first dose of study vaccine until 28 days after administration of the last study vaccine. (See protocol Section 6.8 for collection and recording of concomitant therapies associated with SAEs, solicited and unsolicited AEs, suspected AESIs, and MAAEs. All other concomitant therapies should be recorded if administered in conjunction with new or worsening AEs reported per protocol requirements outlined in Section 8.3.1).
s. On Day 1, the participant's diary, a ruler, and a thermometer will be distributed to each participant. On Day 29, the site is to conduct the last vaccination visit and confirm any missing solicited safety data points.
t. The reactogenicity vaccination folder should be completed and local and systemic symptoms should be verified and entered in eDC. If a solicited event is still ongoing 7 days after vaccination, the participant should continue reporting symptoms until resolution. If symptoms continue beyond Day 7, the diary should be reviewed again at the next visit.

AE adverse event; AESI adverse event of special interest; COVID 19 coronavirus disease 2019; d day(s); eCRF electronic case report form; ICF informed consent form; MAAE medically attended adverse event; meds medication; mo months; N/A not applicable; PCR polymerase chain reaction; SAE serious adverse event; SARS CoV 2 severe acute respiratory syndrome coronavirus 2; vac vaccination; vp virus particles.

1.3.2. Participants with a Suspected AESI

Thrombosis with thrombocytopenia syndrome (TTS) is considered to be an adverse event of special interest (AESI). Suspected AESIs (thrombotic events and thrombocytopenia [defined as platelet count below 150,000/μL (Brighton Collaboration 2021)]) will be monitored from the moment of vaccination until the end of the study/early withdrawal. An AESI Adjudication Committee with appropriate expertise will be established to evaluate each suspected AESI and determine whether it is a case of TTS (further details are provided in Section 8.3.6.1).

Participants should be instructed to seek immediate medical attention if they develop symptoms such as shortness of breath, chest pain, leg swelling, persistent abdominal pain, severe or persistent headaches, blurred vision, and skin bruising and/or petechiae beyond the site of vaccination.

The medical management of thrombotic events with thrombocytopenia is different from the management of isolated thromboembolic diseases. Study site personnel and/or treating physicians should follow available guidelines for treatment of thrombotic thrombocytopenia (eg, from the American Society of Hematology 2021, British Society of Haematology 2021, and the CDC 2021). The use of heparin may be harmful and alternative treatments may be needed. Consultation with a hematologist is strongly recommended. Management of the participant should not be delayed by decision-making of the Janssen Adjudication Committee.

In the event of a suspected post-vaccination thrombotic event, thrombocytopenia, or TTS, laboratory assessments (to be performed locally) might be needed to facilitate diagnosis and determine treatment options, including but not limited to platelet count and anti- platelet factor 4(PF4) tests.

Additional blood samples should be collected as detailed below. However, results of central laboratory testing will not be available to guide immediate treatment decisions.

Timing relative to onset of a suspected AESI	AESI Day 1[a]	AESI Day 29[b]
Visit Window		±7 days
Site to report suspected AESI[c]	●	●
Clinical laboratory blood sample (whole blood / serum / plasma), blood draw, approx. mL[d]	● 15	● 15
TTS AESI form[e]	- - - - - *Continuous* - - - - -	
Concomitant therapies[f]	●	●

a. Day 1 refers to first awareness of the event, which might be later than the date of onset. Every effort should be made to report as much information as possible about the event to the sponsor in a reasonable timeframe. The investigator should contact the sponsor for input on the feasibility of collecting blood samples, including the need for additional samples based on the nature of the event.

b. Day 29 is to be calculated relative to the actual day of onset of the event. If the event is not resolved on Day 29, subsequent follow-up assessments can be performed at unscheduled visits as needed until resolution of the event.

c. Suspected AESIs must be reported to the sponsor within 24 hours of awareness irrespective of seriousness (ie, serious and non-serious AEs) or causality assessment (see Section 8.3.6). Investigators should report potential thrombotic events (if any) and enter confirmation into the participant's eCRF.

d. Whole blood samples will be used for a complete blood count (CBC), including platelet count in a local laboratory or substitute for local laboratory, depending on local feasibility towards turnaround time of sample

processing. Serum and plasma samples will be collected for coagulation-related testing in a central laboratory including anti-PF4 (see Section 10.2).

e. Medical information on local case management will be collected. Upon becoming aware of the suspected AESI, study site personnel should provide information on an ongoing basis. See Section 8.3.6 and Section 10.8, for further details.

f. Refer to Section 6.8 for collection and recording of concomitant therapies associated with a suspected AESI.

AESI = adverse event of special interest; CDC = Centers for Disease Control and Prevention; PF4 = platelet factor 4; TTS = thrombosis with thrombocytopenia syndrome.

2. INTRODUCTION

Ad26.COV2.S (also known as VAC31518, JNJ-78436735) is a monovalent vaccine composed of a recombinant, replication-incompetent human adenovirus type 26 (Ad26) vector, constructed to encode the Spike (S) protein derived from a severe acute respiratory syndrome coronavirus-2 (SARS-CoV-2) clinical isolate (Wuhan, 2019, whole-genome sequence NC_045512, referred to as the original strain), with 2 proline substitutions which stabilize the prefusion conformation of the S-2P protein thereby increasing the induction of neutralizing antibodies.

The Ad26.COV2.S is designed to elicit a protective immune response to the SARS-CoV-2 S protein, the major structural protein of the virus that has an essential role in attachment and infection of host cells (Zhou 2004; Zumla 2016). In the absence of an established immune marker predictive of protection against coronavirus disease-2019 (COVID-19), neutralizing antibodies, primarily directed to the SARS-CoV-2 S protein, are thought to contribute to the protective immune response elicited by the vaccine.

For the most comprehensive nonclinical and clinical information regarding Ad26.COV2.S, refer to the latest version of the Investigator's Brochure (IB) (and Addenda) for Ad26.COV2.S (IB Ad26.COV2.S 2021).

The term “study vaccine” throughout the protocol, refers to Ad26.COV2.S, placebo, and the seasonal quadrivalent *standard-dose* and *high-dose* influenza vaccines as defined in Section 6.1.

Study VAC31518COV3005 is being conducted under the sponsorship of Janssen (Janssen Vaccines & Prevention B.V.).

The term "sponsor" used throughout this document refers to the entities listed in the Contact Information page(s), which will be provided as a separate document. The term “participant” throughout the protocol refers to the common term “subject.”

Adenoviral-vectored Vaccines

Recombinant, replication-incompetent adenoviral vectors are attractive candidates for expression of foreign genes for a number of reasons. The adenoviral genome is well characterized and comparatively easy to manipulate. Adenoviruses exhibit broad tropism, infecting a variety of dividing and non-dividing cells. The adenoviral vaccine (AdVac®) vector platform, developed by Crucell Holland B.V. (now Janssen Vaccines & Prevention B.V.) allows for high-yield production of replication-incompetent adenovirus vectors, eg, Ad26, with desired inserts. The adenovirus E1 region is deleted to render the vector replication-incompetent and create space for transgenes, with viral replication taking place in cells that complement for the E1 deletion in the virus genome. Ad26 has been selected as a potential vaccine vector because there is substantial nonclinical and clinical experience with Ad26-based vaccines that demonstrate their capacity to elicit strong humoral and cellular immune responses and their acceptable safety profile, irrespective of the antigen transgene (see also Section 2.3.1).

The immunogenicity profile of adenoviral vectors is illustrated by data obtained following the immunization of adults with Ad26-vectored human immunodeficiency virus (HIV) vaccines (Ad26.ENVA.01, Ad26.Mos.HIV and Ad26.Mos4.HIV), an Ad26-vectored Ebola virus vaccine (Ad26.ZEBOV), Ad26-vectored respiratory syncytial virus (RSV) vaccines (Ad26.RSV.FA2 and Ad26.RSV.preF), an Ad26-vectored Zika virus vaccine (Ad26.ZIKV.001), and an Ad26-vectored malaria vaccine (Ad26.CS.01). Antigen-specific antibody responses are observed in almost all participants after 1 dose, in both naïve and pre-immune individuals (RSV). These antibodies may persist for a year or more (RSV) after a single vaccination in pre-immune participants. They have functional properties of neutralization (RSV, Zika), crystallizable fragment (Fc)-mediated antibody-dependent cellular cytotoxicity (ADCC) and antibody-dependent cellular phagocytosis (HIV, malaria). Furthermore, these data support an immunogenicity profile with emphasis on T-helper cell type 1 (Th)1 responses and demonstrate predominantly interferon gamma (IFN-γ) and tumor necrosis factor alpha (TNF-α) production in cluster of differentiation (CD) 4^+ and $CD8^+$ T cells (Barouch 2013; Milligan 2016; Mutua 2019; Salisch 2021).

Ad26.COV2.S Candidate Vaccine

The Ad26.COV2.S vaccine has been developed in response to the global outbreak of COVID-19 caused by SARS-CoV-2. In response to the public health emergency caused by the global SARS-CoV-2 pandemic, the Ad26.COV2.S vaccine was granted Emergency Use Authorization (EUA) from the Food and Drug Administration (FDA) on 27 February 2021 for emergency use in the United States (FDA 2021), and Conditional Marketing Authorization approval by the European Medicine Agency (EMA) on 11 March 2021 for emergency use within the European Union (EU). On 12 March 2021, the Ad26.COV2.S vaccine was recommended for emergency use under the World Health Organization (WHO) Emergency Use Listing Procedure.

COVID-19 Disease Burden

SARS-CoV-2 is a highly transmissible and pathogenic coronavirus that has spread rapidly and globally since its emergence in late December 2019. The WHO declared that the outbreak constituted a public health emergency of international concern on 30 January 2020 and declared the outbreak to be a pandemic on 11 March 2020 (WHO 2020; WHO 2020a). As of 11 July 2021, approximately 186,410,350 confirmed global cases of COVID-19 and approximately 4,024,946 COVID-19-related deaths have been reported worldwide. Within the United States alone, as of 11 July 2021 approximately 33,847,474 confirmed cases of COVID-19 and approximately 607,135 COVID-19 related deaths COVID-19 have been reported since the start of the outbreak (Johns Hopkins CSSE 2021). Within the EU/European Economic Area (EEA) countries, as of 11 July 2021, a total of 33,270,049 cases of COVID-19 and 740,809 COVID-19-related deaths were reported (ECDC 2021).

Influenza Disease Burden

Influenza is a worldwide public health problem, responsible for significant morbidity and mortality, as well as a significant resource burden on countries. In a typical year, about 3 to 5 million cases of severe influenza illness and about 290,000 to 650,000 deaths are estimated to occur worldwide (WHO 2018). In the US, more than 200,000 patients are hospitalized as a result

of seasonal influenza-associated illnesses each year (CDC 2021b) and influenza-related hospitalization rate over 15 seasons was estimated to be 309 per 100,000 in individuals 65 years and older (Zhou 2012). Notably, the incidence of pneumonia and influenza hospitalizations increased with age during adulthood, with a dramatic increase seen above the age of 65 years. In the US, older adults aged 85 years and older had the highest rates of influenza-associated primary respiratory and circulatory hospitalizations, ie, 1,194.9 per 100,000 persons. (Bartozsko 2019)

Comorbidities including chronic lung disease and cardiovascular disease were found to be important predictors of hospital admission. There was a 3-fold increase in the risk of death among elderly people compared with non-elderly people. Elderly participants also had a higher risk of admission to the hospital. (Mertz 2013)

Ad26.COV2.S Co-administration With Influenza Vaccines

There is currently no available data for the co-administration of the Ad26.COV2.S vaccine with other vaccines. However, during the COVID-19 pandemic, reducing the overall burden of respiratory illnesses is important to protect vulnerable populations at risk for severe illness, the healthcare system, and other critical infrastructure.

According to the Centers of disease control and prevention (CDC), efforts to reduce the spread of COVID-19, such as stay-at-home and shelter-in-place orders, have led to decreased use of routine preventive medical services, including immunization services. Ensuring that people continue or start getting routine vaccinations during the COVID-19 pandemic is essential for protecting people and communities from vaccine-preventable diseases and outbreaks, including influenza (CDC 2020). This recommendation is particularly relevant for individuals who are more susceptible to respiratory viruses due to risk factors such as comorbidities and old age.

It is recognized that older adults and patients with comorbidities are at greater risk of COVID-19 infection and that they are also more likely to develop severe illness after infection (Perrotta 2020; CDC 2020). Annual influenza vaccination is highly encouraged especially during the ongoing COVID-19 pandemic. Though influenza vaccines do not protect against COVID-19, vaccination may reduce the burden of influenza disease on the health care system, including hospitalizations, severe outcomes, and deaths (Huang 2021). There is an urgent unmet medical need for co-administration of prophylactic vaccines for these 2 deadliest respiratory diseases.

It was recently reported, that vaccine efficacy appeared to be preserved when co-administrating the COVID-19 vaccine NVX-CoV2373 with an influenza vaccine in participants 18 to <65 years of age, and no early safety concerns were observed (Toback 2021).

2.1. Study Rationale

The study aims to assess the safety, reactogenicity, and immunogenicity of the Ad26.COV2.S vaccine co-administered with a quadrivalent *standard-dose* or *high-dose* seasonal influenza vaccine compared to administration of each vaccine separately to explore whether Ad26.COV2.S and the influenza vaccines can be administered concomitantly.

According to the CDC, COVID-19 vaccines were previously recommended to be administered alone, with a minimum interval of 14 days before or after administration of any other vaccines. The initial recommendation was not due to safety or immunogenicity concerns and substantial data have been collected on the safety of authorized COVID-19 vaccines. Guidelines have been updated to allow COVID-19 vaccines and other vaccines to be administered without regard to timing, including co-administration of COVID-19 vaccines and other vaccines on the same day (CDC 2021c).

It is unknown whether the reactogenicity of Ad26.COV2.S can be influenced by co-administration with another vaccine. In certain cases, it has been observed that vaccine co-administration can increase systemic reactogenicity (Sadoff 2020). However, extensive experience with non-COVID-19 vaccines has demonstrated that immunogenicity and adverse event profiles are similar when vaccines are administered simultaneously as when they are administered alone (CDC 2021c). Vaccine's co-administration is generally a safe and effective practice in which the benefits outweigh the risks (Brosio 2018).

The seasonal influenza vaccines that will be used in this study are a quadrivalent *high-dose* influenza vaccine approved for use in persons ≥65 years of age and a quadrivalent *standard-dose* influenza vaccine approved for use in persons ≥18 years of age. These vaccines are indicated for active immunization for prevention of disease caused by influenza A subtype viruses and type B viruses contained in the vaccine. For more details, refer to Section 6.1.

2.2. Background

Nonclinical Pharmacology

Nonclinical pharmacology of Ad26.COV2.S was evaluated in murine, rabbit, Syrian hamster, and non-human primate (NHP) animal models for immunogenicity, including assessment of immunological parameters relevant to the theoretical risk of vaccine-associated enhanced respiratory disease (VAERD). In addition, vaccine efficacy of Ad26.COV2.S including lung histopathology assessment was evaluated in Syrian hamsters and NHPs. Details are provided in the IB (IB Ad26.COV2.S 2021).

Nonclinical Safety

Biodistribution

Ad26 viral vectors (Ad26-based HIV vaccine Ad26.ENVA.01 and Ad26-based RSV vaccine Ad26.RSV.preF) showed a similar pattern of biodistribution and clearance, despite differences in the encoded antigen transgenes when delivered via the intramuscular (IM) route at full human dose levels in the rabbit. Therefore, the biodistribution results are considered sufficient to inform on the biodistribution profile of Ad26.COV2.S, for which the same (replication-incompetent) Ad26 vector backbone is used. The target organs or tissues identified in the Ad26.COV2.S toxicology study (ie, injection site, [draining] lymph nodes, and spleen) are consistent with the tissues that tested mostly positive for Ad26 DNA in the biodistribution studies for the Ad26 platform. Details are provided in the IB (IB Ad26.COV2.S 2021).

Toxicology

In a Good Laboratory Practice (GLP)-compliant repeat-dose toxicity and local tolerance study in rabbits, Ad26.COV2.S was well tolerated when administered on 3 occasions over 4 weeks (ie, every 2 weeks) at 1×10^{11} virus particles (vp)/dose. The observed changes were related to a normal, anticipated (local and systemic) immunologic response to vaccination. Overall, the findings were considered non-adverse and were partially or completely reversible after a 3-week treatment-free period. Details are provided in the IB (IB Ad26.COV2.S 2021).

Reproductive and Developmental Toxicology

There was no harmful effect of Ad26.COV2.S concerning female reproductive toxicity and fertility as assessed in a combined embryo-fetal and pre- and postnatal development toxicity study in the rabbit. No adverse effects were observed on reproductive performance, fertility, ovarian and uterine examinations, or parturition. In addition, there was no adverse effect of vaccination on fetal body weights, external, visceral and skeletal evaluations, or on postnatal development of the offspring. The parental females as well as their fetuses and offspring exhibited SARS-CoV-2 S protein-specific antibody titers, indicating that maternal antibodies were transferred to the fetuses during gestation. Details are provided in the IB (IB Ad26.COV2.S 2021).

Clinical Studies

At the time of protocol writing, Phase 1/2a (COV1001, COV1002) and Phase 2a (COV2001) clinical studies to assess the safety, reactogenicity, and immunogenicity of Ad26.COV2.S, as well as Phase 3 (COV3001, COV3009, COV3003) clinical studies to assess the efficacy of Ad26.COV2.S in the prevention of molecularly confirmed moderate to severe/critical COVID-19 in adults are ongoing and data from interim analyses from these studies, except COV3009, are available.

Efficacy

A single dose of Ad26.COV2.S at 5×10^{10} vp protects against COVID-19 in adults ≥18 years of age, including adults ≥60 years of age. Based on the primary efficacy analysis of the pivotal Phase 3 study COV3001, including 19,630 participants who received Ad26.COV2.S and 19,691 participants who received placebo, vaccine efficacy (adjusted 95% confidence interval [CI]) for the co-primary endpoints against molecularly confirmed moderate to severe/critical COVID-19 in participants who were seronegative at time of vaccination was 66.9% (59.03; 73.40) when considering cases from at least 14 days after vaccination and 66.1% (55.01; 74.80) when considering cases from at least 28 days after vaccination. Ad26.COV2.S has been shown to be effective against all symptomatic COVID-19, highly effective in the prevention of severe/critical COVID-19 (particularly in the prevention of hospitalization and death, across all countries and all ages), and effective against newly emerging strains, such as the 20H/501Y.V2 strain.

Safety

The most extensive safety information of the single-dose regimen of Ad26.COV2.S at $5×10^{10}$ vp is available from ≥43,000 participants, 18 years of age and above, including adults 60 years of age and above, enrolled in the ongoing pivotal Phase 3 study COV3001. A 2-dose vaccine regimen of Ad26.COV2.S at $5×10^{10}$ vp is currently under evaluation in the ongoing Phase 3 study COV3009. Supportive safety information (adverse events [AEs], clinical laboratory abnormalities, vital signs, and physical examination findings) is available from the interim analysis of the Phase 1 and 2 studies COV1001, COV1002, and COV2001. In COV2001, Ad26.COV2.S is being evaluated at a range of doses and intervals ($1.25×10^{10}$ vp, $2.5×10^{10}$ vp, $5×10^{10}$ vp, and $1×10^{11}$ vp) in adolescents 12 to 17 years of age, adults 18 to 55 years of age, and ≥65 years of age.

Ad26.COV2.S has an acceptable safety and reactogenicity profile when administered as a single-dose or as a 2-dose regimen in adults ≥18 years of age, including adults ≥60 years of age. No significant safety issues were identified. In general, a lower reactogenicity profile was observed in older adults compared to younger adults.

Overall, up to 22 January 2021, no safety concerns were identified after vaccination with Ad26.COV2.S as a single-dose or 2-dose vaccine regimen at dose levels up to $1×10^{11}$ vp. There was a trend towards a decrease in the frequency of solicited local and systemic adverse events (AEs) (reactogenicity) with a decreasing dose level of Ad26.COV2.S (from $1×10^{11}$ vp to $2.5×10^{10}$ vp). Reactogenicity was demonstrated to be transient and most solicited AEs, including pyrexia, generally resolved in 1 to 2 days post-vaccination. In the ongoing Phase 3 study COV3001, out of over 43,000 participants, 19 deaths were reported, 3 in the Ad26.COV2.S group and 16 in the placebo group, all of which were considered unrelated to the study vaccine by the investigator. No AEs with a fatal outcome have been reported in the supportive Phase 1 and Phase 2 studies COV1001, COV1002, and COV2001. In the COV3001 study, at the time of the primary analysis, 0.4% participants in the Ad26.COV2.S group and 0.6% participants in the placebo group reported 1 or more serious adverse events (SAEs). Ten (<0.1%) participants reported SAEs that were considered to be related to the study vaccine: 7 SAEs were reported in 7 participants in the Ad26.COV2.S group: Grade 4: Guillain-Barré syndrome and pericarditis (one of each); Grade 3: radiculitis brachial, post-vaccination syndrome, and Type IV hypersensitivity (one of each); Grade 2: facial paralysis (Bell's Palsy - 2 cases reported); 3 SAEs were reported in 2 participants in the placebo group: Grade 4: deep vein thrombosis (DVT) (one participant); Grade 3: Epstein-Barr virus infection and atrial flutter (both were reported in the same participant). No participants in either vaccine group (Ad26.COV2.S or placebo) were withdrawn from the study due to AEs. Across studies COV1001, COV1002, and COV2001, 2 participants reported SAEs which were considered to be related to the study vaccine (both occurred in study COV1001). Early discontinuations of vaccination or the study due to (S)AEs were infrequent in all groups.

Since then, 6 cases of severe allergic reactions were reported in study COV3012 (Sisonke [Together]), an open-label, single-arm Phase 3b vaccine implementation study sponsored by the South African Medical Research Council and conducted in collaboration with Janssen, which is currently ongoing in the Republic of South Africa. One case met the Brighton Collaboration case

definition of anaphylaxis with level 2 of diagnosis certainty (Rüggeberg 2007). The review of the cases of severe allergic reactions reported from this study identified a plausible causal relationship between the administration of the Ad26.COV2.S vaccine and the occurrence of severe allergic reactions including anaphylaxis.

Cases of thrombotic events with thrombocytopenia have very rarely been observed in individuals who received Ad26.COV2.S. At the time of primary analysis of study COV3001 (Data lock point 22 January 2021), one case of cerebral venous sinus thrombosis (CVST) with thrombocytopenia had been reported which triggered a temporary pause in vaccinations across Ad26.COV2.S clinical studies. One additional case of DVT with thrombocytopenia was since reported in study COV3001. As of 17 April 2021, 6 spontaneous reports of CVST with thrombocytopenia and 1 spontaneous report of DVT with thrombocytopenia have been reported from the US in the context of the routine vaccination programs, one of which had a fatal outcome. At that time, more than 7.9 million people had received Ad26.COV2.S in the US.

Immunogenicity

Across studies COV1001, COV1002, and COV2001, a single dose of Ad26.COV2.S was shown to elicit SARS-CoV-2 neutralizing antibodies in both the 18 to 55 years age group and in adults ≥65 years of age. In studies COV1001 and COV3001, SARS-CoV-2 S protein binding antibodies were elicited in the vast majority of the 18 to 55 years age group and in adults ≥65 years of age.

In study COV1001, Ad26.COV2.S induced CD4+ T cell responses with a T-helper cell (Th)1 dominant phenotype, and CD8+ T cell responses in both 18 to 55 and ≥65 years of age participants.

Refer to the latest IB (and Addenda) for more details on the ongoing clinical studies with Ad26.COV2.S (IB Ad26.COV2.S 2021).

Clinical Safety Experience With Ad26-based Vaccines

Safety of Ad26-vectored vaccines has been evaluated in adults in clinical studies by the sponsor. Replication-incompetent Ad26 is being used as a vector in the development of vaccines against diseases such as malaria, RSV, HIV, Zika virus, filovirus, and human papillomavirus (HPV), and has been used in the now licensed Ebola virus vaccine (Zabdeno/Ad26.ZEBOV) and for emergency use authorized COVID-19 vaccine (Ad26.COV2.S).

As of 17 April 2021, Ad26-based vaccines developed by the sponsor have been administered to more than 8 million participants. The majority of these participants are enrolled in an ongoing immunization campaign in the US (COVID-19 Vaccine Program Campaign) (more than 7.9 million) and an ongoing Ebola vaccine study in the Democratic Republic of the Congo and an ongoing immunization campaign in Rwanda (UMURINZI Ebola Vaccine Program campaign) (more than 156,000).

Overall, the Ad26-based vaccines were well tolerated irrespective of the antigen transgene, without significant safety issues. Recently, rare disorders including blood clots in combination

with low platelets have been observed after vaccination with Ad26-based vaccines, including the Ad26.COV2.S vaccine (refer to Section 2.3.1 for more details).

2.3. Benefit-Risk Assessment

More detailed information about the known and expected benefits and risks of Ad26.COV2.S may be found in the IB and its addenda (IB Ad26.COV2.S 2021), and package insert.

2.3.1 Risks Related to Study Participation

The following potential risks for Ad26.COV2.S will be monitored during the study and are specified in the protocol:

Risks Related to Ad26.COV2.S

At the time of initial protocol writing, efficacy, immunogenicity, and safety data were available from the ongoing clinical studies COV1001, COV1002, COV2001, COV3001, COV3009, and COV3012.

Adverse reactions are defined as AEs for which a causal relationship between vaccination with Ad26.COV2.S and its occurrence is reasonably established, based on a comprehensive assessment of the available AE information. The AR analysis was performed for individuals ≥18 to <65 years of age and ≥65 years of age. The dataset for AR identification (see Table 1) was derived from the ongoing Phase 3 study COV3001, including 43,783 adults aged ≥18 years of age (35,222 adults ≥18 to <65 years of age and 8,561 adults ≥65 years of age). The most common local AR reported was injection site pain (48.7%). The most common systemic ARs were headache (39.0%), fatigue (38.3%), myalgia (33.2%), and nausea (14.2%). Pyrexia was observed in 9.1% of participants. Most ARs occurred within 1-2 days following vaccination and were mild to moderate in severity and of short duration (1-2 days).

Reactogenicity was generally milder and reported less frequently in older adults (≥65 years of age).

Table 1: Adverse Drug Reactions Associated With Ad26.COV2.S

Solicited ADRs	Adverse Events	Number of participants in the safety cohort exposed to Ad26.COV2.S (N) = 3,356	
**Local Reactogenicity Symptoms System Organ Class (SOC)**	**Preferred Term**	**Frequency (%)**	**Frequency Category**
General disorders and administration site conditions	Injection site pain	1634 (48.7%)	Very common
	Vaccination site erythema	245 (7.3%)	Common
	Vaccination site swelling	178 (5.3%)	Common
**Systemic Reactogenicity Symptoms System Organ Class (SOC)**	**Preferred Term**	**Frequency (%)**	**Frequency Category**
Nervous system disorders	Headache	1308 (39.0%)	Very common
Gastrointestinal disorders	Nausea	478 (14.2%)	Very common
Musculoskeletal and connective tissue disorders	Myaglia	1115 (33.2%)	Very common
General disorders and administration site conditions	Fatigue	1286 (38.3%)	Very common
	Pyrexia (Fever)	302 (9,1%)	Common

Unsolicited ADRs	Adverse Event	Number of participants in the full cohort exposed to Ad26.COV2.S (N) = 21,895	
**System Organ Class (SOC)**	**Preferred Term**	**Frequency (%)**	**Frequency Category**
Skin and subcutaneous tissue disorders	Rash	24 (0.1%)	Uncommon
Immune system disorders	Hypersensitivity	6 (<0.1%)	Rare
	Urticaria	8 (<0.1%)	Rare
**Unsolicited ADRs**	**Adverse Events**	**Number of participants in the safety cohort exposed to Ad26.COV2.S (N) = 3,356**	
General disorders and administration site conditions	Chills	67 (2.0%)	Common
	Malaise	26 (0.8%)	Uncommon
	Asthenia	18 (0.5%)	Uncommon
Musculoskeletal and connective tissue disorders	Arthralgia	35 (1.0%)	Common
	Muscular weakness	10 (0.3%)	Uncommon
	Pain in extremity	9 (0.3%)	Uncommon

Allergic Reactions (Hypersensitivity) and Severe Allergic Reactions (Anaphylaxis)

Severe allergic reactions, including anaphylaxis, have been reported following the administration of Ad26.COV2.S, 1 case in Study COV3001 (as of a cutoff of 05 March 2021), and 6 cases in Study COV3012 (Sisonke [Together]) (as of a cutoff of 23 March 2021). The review of the cases of severe allergic reactions reported from Study COV3012 identified a plausible causal relationship between the administration of the Ad26.COV2.S vaccine and the occurrence of severe allergic reactions including anaphylaxis, with 1 case meeting the Brighton Collaboration case definition of anaphylaxis with Level 2 of diagnosis certainty. Based on these findings of severe allergic reactions including anaphylaxis and clear biological plausibility, and in consultation with EMA and FDA, anaphylaxis is considered an AR with unknown frequency.

Individuals should be observed by a healthcare provider after vaccination per protocol requirements.

Adverse Events of Interest

Thrombosis with Thrombocytopenia Syndrome [TTS]

Thrombosis in combination with thrombocytopenia (thrombosis with thrombocytopenia syndrome [TTS]), has been observed very rarely following vaccination with Ad26.COV2.S. These cases occurred approximately 1-2 weeks following vaccination, mostly in adult women (CDC 2021b). Even though thrombosis in combination with thrombocytopenia can be fatal,the exact physiology of TTS is unclear. TTS is considered a post-vaccination risk for Ad26.COV2.S vaccine.

An analysis of TTS cases identified by the Vaccine Adverse Event Reporting System (VAERS) following administration of the COVID-19 vaccine Janssen in the US was performed by the CDC. It identified 28 TTS cases as of 07 May 2021 after administration of almost 9 million doses of the Ad26.COV2.S vaccine. Based on their own risk/benefit analysis, the CDC endorsed continued support of the use of Ad26.COV2.S. COVID-19 vaccine Janssen vaccine (Shimabukuro 2021). In May 2021, the CDC presented the updated Risk/Benefit analysis with the updated incidence of TTS and concluded that the benefits of Ad26.COV2.S. COVID-19 vaccine Janssen vaccine outweighed the risks (Lee 2021). Following the recommendation from the US CDC Advisory

Committee on Immunization Practices (ACIP), which confirmed a positive benefit-risk profile for the Janssen COVID-19 vaccine (MacNeil 2021), the US CDC and the FDA, have recommended vaccinations with the Ad26.COV2.S vaccine for all adults aged 18 years and older. Healthcare professionals should be mindful of signs and symptoms of thromboembolism and/or thrombocytopenia.

Participants should be instructed to seek immediate medical attention if they develop symptoms such as shortness of breath, chest pain, leg swelling, persistent abdominal pain, severe or persistent headaches, blurred vision, and skin bruising and/or petechiae beyond the site of vaccination. The medical treatment of thrombosis with thrombocytopenia is different from standard blood clot treatments. Study site personnel and/or treating physicians should follow available guidelines for the treatment of thrombotic thrombocytopenia (eg, from the American Society of Hematology 2021, British Society of Haematology 2021, and the CDC 2021). The use of heparin may be harmful and alternative treatments may be needed. Consultation with a hematologist is strongly recommended. Management of the participant should not be delayed by the decision-making of the Janssen Adjudication Committee. Due to the possibility of the occurrence of TTS after vaccination with Ad26.COV2.S, additional reporting, and data collection procedures have been included in the study for thrombotic events, thrombocytopenia, and TTS (see Section 8.3.6), which may facilitate diagnosis and clinical management of the event.

Heparin-induced Immune Thrombotic Thrombocytopenia

TTS has been found to resemble heparin-induced thrombocytopenia with thrombosis (HITT), which is mediated by antibodies against complexes of heparin and platelet factor 4 (PF4) that induce Fc receptor-mediated activation of platelets and hypercoagulation (Arepally 2021). Current evidence suggests that these 2 conditions may be linked (Streiff 2021). The exact mechanisms remain to be investigated, but anti-PF4 antibodies are suspected to be involved in the pathogenesis of TTS associated with vaccination.

Risks Related to Adenoviral-vectored Vaccines

The sponsor's clinical AdVac® safety database contains pooled safety data from 32 completed and ongoing unblinded clinical studies using the following Ad26-based vaccines: Ad26.ZEBOV (Ebola; 10 studies), Ad26.ENVA.01, Ad26.Mos.HIV, and Ad26.Mos4.HIV (HIV; 8 studies), Ad26.CS.01 (malaria; 1 study), Ad26.RSV.FA2 and Ad26.RSV.preF (RSV; 10 studies), Ad26.Filo (filovirus; 1 study), Ad26.ZIKV.001 (Zikavirus; 1 study), and Ad26.HPV16 and Ad26.HPV18 (HPV; 1 study). A total of 8,152 adult participants (in 32 studies; 2,087 adults aged 18-30 years, 1,738 adults aged 31-50 years, 182 adults aged 51-59 years, and 4,145 adults aged 60 years and older) and 674 children (in 3 studies; 168 children aged 1-3 years, 252 children aged 4-11 years, and 254 children aged 12-17 years) were vaccinated with an Ad26-based vaccine. The total number of Ad26-based vaccine doses administered was 10,867 (10,169 for adults, and 698 for children) administered to 8,826 individuals. Overall, the Ad26-based vaccines were well tolerated, without significant safety issues identified.

Risks Related to Afluria Quadrivalent and Fluzone High-dose Quadrivalent

Afluria Quadrivalent is licensed to be used for individuals aged ≥6 months and Fluzone High-dose Quadrivalent is licensed to be used for individuals aged ≥65 years. These seasonal quadrivalent *standard-dose* and *high-dose* influenza vaccines will be administered in the study following the approved prescribing information.

Adverse Reactions Afluria Quadrivalent

In adults 18 to 64 years of age, the most common (≥10%) solicited local AR was pain (47.9%); the most common solicited systemic ARs were myalgia (25.5%) and headache (21.7%).

In adults 65 years of age and older, the most common (≥10%) solicited local AR was pain (24.6%); the most common solicited systemic AR was myalgia (12.7%).

Adverse Reactions Fluzone High-dose Quadrivalent

In adults 65 years of age and older, the most common (≥10%) solicited local AR was pain (41.3%), the most common solicited systemic ARs were myalgia (22.7%), headache (14.4%), and malaise (13.2%).

For further details, refer to the Afluria Quadrivalent and Fluzone High-dose Quadrivalent package inserts.

General Risks Related to Vaccination

In general, intramuscular (IM) injection may cause local itching, warmth, pain, tenderness, erythema/redness, induration, swelling, arm discomfort, or bruising of the skin. Participants may exhibit general signs and symptoms associated with IM injection of a vaccine and/or placebo, including fever, chills, rash, myalgia, nausea/vomiting, headache, dizziness, arthralgia, general itching, and fatigue. These side effects will be monitored, but are generally short-term and do not require treatment. Instructions regarding the use of antipyretic medication can be found in Section 6.8.

Syncope can occur in association with the administration of injectable vaccines. Syncope can be accompanied by falls. Procedures should be in place to avoid falling injury. If syncope develops, participants should be observed until the symptoms resolve. Fear of injection might lead to fainting and fast breathing.

Participants may have an allergic reaction to the vaccination. An allergic reaction may cause a rash, urticaria, or even anaphylaxis (see above risks related to Ad26.COV2.S). Severe reactions are rare. Participants with a known allergy or history of anaphylaxis, or other serious adverse reactions to vaccines or their excipients (including specifically the excipients of the study vaccine) will be excluded from the study per the investigator's judgment.

After vaccination, participants will remain at the study site for at least 15 minutes and will be closely observed by the study staff. Necessary emergency equipment and medications must be available in the clinic to treat severe allergic reactions.

Pregnancy and Birth Control

The effect of the Ad26.COV2.S vaccine on the fetus or a nursing baby is unknown. Active vaccination of pregnant women is currently under evaluation in the COV2004 clinical study. Despite the cumulative review of pregnancy cases from completed and ongoing clinical studies as well as a post-marketing mass vaccination campaign for Ad26 vectored vaccine products does not reveal any safety concern related to vaccine exposure during pregnancy, pregnant women will not take part in this study.

Participants of childbearing potential will be required to agree to practice a highly effective method of contraception and agree to remain on such a method of contraception from providing consent until 3 months after receiving the last study vaccine (see Section 5.1). Participants who are pregnant at screening will be excluded from the study. Participants who become pregnant during the study may continue study vaccination if allowed by local regulations for emergency use of vaccines and if the investigator considers that the potential benefits outweigh any potential risks to the mother and fetus. Participants will remain in the study and will continue to undergo all procedures as outlined in the protocol (see also Section 8.3.5).

Participants who are breastfeeding are not allowed to participate in the study.

Risks from Blood Draws

Blood draws may cause pain, tenderness, bruising, bleeding, dizziness, vasovagal response, syncope, and rarely, infection at the site where the blood is taken.

Concomitant Vaccination

Concomitant vaccination of the Ad26.COV2.S vaccine with a seasonal quadrivalent influenza vaccine might influence both the safety profile and immunogenicity of the Ad26.COV2.S vaccine and/or the seasonal quadrivalent influenza vaccine.

Theoretical Risk of Enhanced Disease

Vaccine-associated enhanced disease (VAED) has been linked to a Th2-polarized immune response and inadequate induction of neutralizing antibody responses and it was characterized by enhanced lung histopathology in nonclinical challenge models of other coronaviruses or RSV (Agrawal 2016; Bolles 2011; Deming 2006; Honda-okubo 2015; Houser 2017). There is, therefore, a theoretical risk VAED, including VAERD, for SARS-CoV-2 vaccines. The potential of Ad26.COV2.S to predispose for VAERD and VAED has been evaluated in nonclinical challenge models established by the sponsor and partners. Ad26.COV2.S immunized Syrian hamsters and NHP showed the absence of enhanced lung pathology and clinical signs of disease compared with controls after SARSCoV2 inoculation, even under conditions of suboptimal immunity allowing breakthrough infection. Together with induction of neutralizing antibodies and a Th1 dominant

immune response after Ad26.COV2.S dosing these data suggest that the theoretical risk of VAERD and VAED for Ad26.COV2.S is low (He 2021; van der Lubbe 2021). In both species, no signs of enhanced respiratory tract pathology were observed in animals dosed with Ad26.COV2.S compared with challenge control groups.

VAERD has so far not been reported in any of the clinical studies, including interim data reported from Phase 2/3 clinical trials of mRNA-based vaccines and an adenovector-based vaccine, expressing SARS-CoV-2 S protein as antigen (Baden 2020; Polack 2020; Voysey 2021). Similarly, no cases of VAERD have been reported in any of the ongoing clinical studies conducted to date by the sponsor, including the Phase 1, Phase 2a and Phase 3 studies. The Ad26 vaccine vector platform has been assessed in several nonclinical and clinical studies so far, and uniformly induced potent humoral and cellular immune responses with a clear Th1 bias (Barouch 2018; Salisch 2019; van der Fits 2020; Widjojoatmodjo 2015). The Ad26.COV2.S vaccine candidate, which encodes the SARS-CoV-2 S protein, has been shown to induce S protein binding antibodies and virus neutralizing antibodies in mice, rabbits, hamsters and NHP. Ad26.COV2.S was shown to induce a Th1 skewed immune response in mice and in NHP including aged NHP. Immunogenicity data from Study COV1001 demonstrated that a single vaccination with Ad26.COV2.S induces robust neutralizing and binding antibody, CD4+ T cell responses with a Th1-dominant phenotype, and CD8+ T cell responses (Anywaine 2019; Barouch 2013; Barouch 2018; Sadoff 2021)

Unknown Risks

There may be other risks that are not known. If any significant new risks are identified, the investigators and participants will be informed.

2.3.2 Benefits of Study Participation

Participants may benefit from clinical testing and physical examination.

The efficacy, immunogenicity, and safety data to date support a favorable benefit-risk profile for Ad26.COV2.S in the proposed indication, ie, active immunization to prevent COVID-19 caused by SARS-CoV-2 in adults ≥18 years of age (see Section 2.2). The overall benefit and risk balance for individual participants is ongoing.

Vaccination with a seasonal quadrivalent influenza vaccine may provide protection against the influenza A subtype viruses and type B viruses contained in the vaccine during the influenza season.

2.3.3 Benefit-Risk Assessment of Study Participation

Based on the available data and proposed safety measures, the overall benefit-risk assessment for this clinical study is considered acceptable for the following reasons:

- A single dose of Ad26.COV2.S protects against moderate to severe/critical COVID-19 as early as 14 days after vaccination as demonstrated in adults ≥18 years of age, including adults ≥60 years of age.

- Only participants who meet all inclusion criteria and none of the exclusion criteria (specified in Section 5) will be allowed to participate in this study. The selection criteria include adequate provisions to minimize the risk and protect the well-being of participants in the study.
- Safety will be closely monitored throughout the study:

  In general, safety evaluations will be performed at scheduled visits during the study, as indicated in the Schedule of Activities (Section 1.3).

  After each vaccination, participants will remain at the study site for at least 15 minutes and will be closely observed by the study staff. Necessary emergency equipment and medications must be available in the clinic to treat severe allergic reactions. Participants will use a diary to document solicited signs and symptoms. Details are provided in Section 8.2.

  The investigator or the designee will document unsolicited AEs, SAEs, adverse events of special interest (AESIs), and medically attended adverse events (MAAEs), as indicated in Section 8.2, Section 8.3, and Section 10.4.

  TTS is considered to be an AESI (Section 8.3.6). Suspected AESIs (thrombotic events and thrombocytopenia [defined as platelet count below 150,000/μL] [Brighton Collaboration 2021] post-vaccination) must be reported to the sponsor within 24 hours of awareness. Suspected AESIs will be followed-up as described in the Schedule of Activities in Section 1.3.2.

  Any clinically significant abnormalities will be followed by the investigator until resolution or until clinically stable. For those abnormalities persisting at the end of the study/early withdrawal, the outcome should be documented in the participant's medical chart.
- Several safety measures are included in this protocol to minimize the potential risk to participants, including the following:

  Eligibility will be reassessed pre-vaccination on Day 1.

  Study vaccinations will be discontinued in participants for the reasons included in Section 7.

  Conditions necessitating a delay in vaccination are included in Section 5.5.

3. OBJECTIVES AND ENDPOINTS

Objectives	Endpoints
**Primary**	
To demonstrate the non-inferiority (NI) of the humoral immune response of the 4 influenza vaccine strains after concomitant administration of the Ad26.COV2.S vaccine and a seasonal quadrivalent *standard-dose* influenza vaccine versus the administration of a seasonal quadrivalent *standard-dose* influenza vaccine administered alone.	• Antibody hemagglutination inhibition (HI) titers as measured by hemagglutinin inhibition (HAI) assay titers (geometric mean titers [GMTs]) against each of the 4 influenza vaccine strains, 28 days after the administration of a seasonal quadrivalent *standard-dose* influenza vaccine<br>*Success criteria for NI*<br>• the upper bound of the 2-sided 95% confidence interval (CI) for the geometric mean titer (GMT) ratio (Control group/co-administration [CoAd] group) lies below 1.5
To demonstrate the NI of the binding antibody response after concomitant administration of Ad26.COV2.S vaccine and a seasonal quadrivalent *standard-dose* influenza vaccine versus the administration of Ad26.COV2.S vaccine administered alone.	• Antibody titers as measured by S enzyme-linked immunosorbent assay (S-ELISA) titers (GMC), 28 days after administration of Ad26.COV2.S vaccine.<br>*Success criteria for NI*<br>• the upper bound of the 2-sided 95% CI for the GMC ratio (Control group/CoAd group) lies below 1.5
**Secondary**	
To assess the safety and reactogenicity of a single dose of Ad26.COV2.S vaccine when administered separately or concomitantly with a seasonal quadrivalent *high-dose* influenza vaccine in participants aged 65 years and older.	• Solicited local (injection site) and systemic AEs for 7 days after each vaccination.<br>• Unsolicited adverse events (AEs) for 28 days after each vaccination.<br>• SAEs, MAAEs, and AESIs throughout the study.<br>• AEs leading to withdrawal from the study throughout the study.

Objectives	Endpoints
To assess the safety and reactogenicity of a single dose of Ad26.COV2.S vaccine when administered separately or concomitantly with a seasonal quadrivalent *standard-dose* influenza vaccine in participants aged 18 years and older.	• Solicited local (injection site) and systemic AEs for 7 days after each vaccination.<br>• Unsolicited AEs for 28 days after each vaccination.<br>• SAEs, MAAEs, and AESIs throughout the study.<br>• AEs leading to withdrawal from the study throughout the study.
To assess the humoral immune response against each of the 4 influenza vaccine strains after concomitant administration of the Ad26.COV2.S vaccine and a seasonal quadrivalent *high-dose* influenza vaccine versus the administration of a seasonal quadrivalent *high-dose* influenza vaccine administered alone.	• Antibody HI titers as measured by HAI assay titers (GMTs) against each of the 4 influenza vaccine strains, 28 days after the administration of a seasonal quadrivalent *standard-dose* influenza vaccine.
To assess the humoral immune response of Ad26.COV2.S vaccine and a seasonal quadrivalent *high-dose* standard-dose influenza vaccine versus the administration of Ad26.COV2.S vaccine administered alone.	• Antibody titers as measured by S enzyme-linked immunosorbent assay (S-ELISA) titers (GMC), 28 days after administration of Ad26.COV2.S vaccine.
To assess the humoral response to SARS-CoV-2 after the concomitant administration of Ad26.COV2.S vaccine and a seasonal quadrivalent *high-dose* influenza vaccine versus the administration of Ad26.COV2.S vaccine alone *in COVID-19 vaccine-naïve individuals*.	• Antibody titers as measured by S enzyme-linked immunosorbent assay (S-ELISA) titers (GMC), 28 days after administration of Ad26.COV2.S vaccine.
To assess the humoral response to SARS-CoV-2 after the concomitant administration of Ad26.COV2.S vaccine and a seasonal quadrivalent *standard-dose* influenza vaccine versus the administration of Ad26.COV2.S vaccine alone *in COVID-19 vaccine-naïve individuals*	• Antibody titers as measured by S enzyme-linked immunosorbent assay (S-ELISA) titers (GMC), 28 days after administration of Ad26.COV2.S vaccine.

Objectives	Endpoints
To compare seroconversion rates against the 4 influenza vaccine strains after the concomitant administration of Ad26.COV2.S vaccine and a seasonal quadrivalent (*high-dose* and *standard-dose*) influenza vaccine versus a vaccination of a seasonal quadrivalent (*high-dose* and *standard-dose*) influenza vaccine administered alone.	• Seroconversion is defined for each of the 4 influenza vaccine strains at 28 days after the administration of a seasonal quadrivalent (*high-dose* and *standard-dose*) influenza vaccine:<br>HI titer ≥1:40 in participants with a pre-vaccination HI titer of <1:10, or<br>a ≥4-fold HI titer increase in participants with a pre-vaccination HI titer of ≥1:10.
To assess seroprotection rates against the 4 influenza vaccine strains after the concomitant administration of Ad26.COV2.S vaccine and a seasonal quadrivalent (*high-dose* and *standard-dose*) influenza vaccine versus a vaccination of a seasonal quadrivalent (*high-dose* and *standard-dose*) influenza vaccine administered alone.	• Seroprotection is defined for each of the 4 influenza vaccine strains as HI titer ≥1:40 at 28 days after the administration of a seasonal quadrivalent (*high-dose* and *standard-dose*) influenza vaccine.
**Exploratory**	
To evaluate the durability of influenza-specific humoral immune responses after concomitant administration of the vaccines versus the influenza vaccine alone.	• GMT: Geometric mean of HI antibodies at Day 181.<br>• Percentages of participants with HI titers ≥1:40 on Day 181.
To evaluate the durability of SARS-CoV-2 specific humoral immune responses after concomitant administration of the vaccines versus the SARS-CoV-2 vaccine alone.	• Antibody GMC by S-ELISA at Day 181 after the administration of Ad26.COV2.S vaccine.
To assess the magnitude and durability of humoral immune responses specific to influenza.	• Serological responses to vaccination as measured by HAI at Day 181 after the administration of influenza vaccine.

Objectives	Endpoints
To further assess humoral responses to Ad26.COV2.	• Serological response to vaccination, as measured by virus neutralization assay (VNA) (SARS-CoV-2 VNA and/or pseudovirion [ps] VNA expressing S protein) titers.<br>• Adenovirus 26 neutralization responses measured by VNA.<br>• Functional and molecular antibody characterization including Fc-mediated viral clearance, avidity, Fc characteristics, immunoglobulin (Ig) subclass, and IgG isotype.<br>• Correlation between ELISA (S-ELISA) and VNA (wild-type virus [wt]VNA and/or pseudovirion [ps]VNA) titers at selected timepoints.
To explore the humoral responses to Ad26.COV2.S and influenza vaccine based on the SARS-CoV-2 serostatus at baseline (N-serology).	• Serological response to vaccination as measured by S-ELISA antibody concentration 28 days after the administration of Ad26.COV2.S vaccine.<br>• Antibody GMC by S-ELISA at 28 days after the administration of Ad26.COV2.S vaccine.<br>• Antibody HI titers as measured by HAI assay titers (GMTs) against the 4 influenza strains.
To explore the humoral responses to Ad26.COV2.S and influenza vaccine based on the previous history of COVID-19 vaccination at entry (viral vector vaccine or mRNA vaccine or vaccine-naïve).	• Serological response to vaccination as measured by S-ELISA antibody concentration 28 days after the administration of Ad26.COV2.S vaccine.<br>• Antibody GMC by S-ELISA at 28 days after the administration of Ad26.COV2.S vaccine.<br>• Antibody HI titers as measured by HAI assay titers (GMTs) against SARS-CoV-2.

Refer to Section 8, Study Assessments and Procedures for evaluations related to endpoints.

HYPOTHESES

The primary hypotheses of this study are:

The concomitant administration of the Ad26.COV2.S vaccine and a seasonal quadrivalent influenza vaccine (*standard-dose*) is non-inferior than the administration of the seasonal quadrivalent influenza vaccine (*standard-dose*) alone as measured by HI titers against each of the 4 influenza vaccine strains at 28 days after the administration of a quadrivalent seasonal influenza vaccine,

AND

The concomitant administration of the Ad26.COV2.S vaccine and a seasonal quadrivalent influenza vaccine (*standard-dose*) is non-inferior than the administration of the Ad26.COV2.S vaccine alone as measured by S-ELISA antibody titers at 28 days after the administration of the Ad26.COV2.S vaccine.

For the high-dose groups, no formal hypothesis testing is planned.

No formal statistical testing of safety data is planned. Safety data will be analyzed descriptively by vaccine group.

4. STUDY DESIGN

4.1. Overall Design

This is a randomized, double-blind, parallel, multicenter, interventional study in healthy (including stable comorbidities) adults ≥18 years of age. In this study, the safety, reactogenicity, and immunogenicity of Ad26.COV2.S co-administered with an influenza vaccine will be evaluated.

Approximately 1,100 participants will be randomized in parallel in this study. Participants aged ≥65 will be randomized in a 1:1:1:1 ratio to 1 of 4 groups. Participants aged ≤64 will be randomized in a 1:1 ratio to 1 of 2 groups (Group 1 or Group 2). Efforts will be made to ensure good representation in terms of race, gender, and ethnicity.

Participants will receive Ad26.COV2.S and a seasonal quadrivalent (*standard-dose* or *high-dose*) influenza vaccine either concomitantly on Day 1 and placebo on Day 29 (co-administration groups 1 and 3) or a seasonal quadrivalent (*standard-dose* or *high-dose*) influenza vaccine and placebo on Day 1 and Ad26.COV2.S on Day 29 (control groups 2 and 4) (see Table 2).

Table 2: Overview of the Groups and Vaccination Days

Group	Day 1	Day 29
1 (CoAd group)	Ad26.COV2.S + Q SD influenza vaccine	Placebo
2 (Control group)	Placebo + Q SD influenza vaccine	Ad26.COV2.S
3 (CoAd group)	Ad26.COV2.S + Q HD influenza vaccine	Placebo
4 (Control group)	Placebo + Q HD influenza vaccine	Ad26.COV2.S

CoAd = co-administration; HD = high-dose; Q = quadrivalent; SD = standard-dose.

The seasonal quadrivalent *standard-dose* influenza vaccine (Groups 1 and 2) can be administered to all participants. Age is included as stratification factor for Groups 1 and 2 (≥18 to ≤64 years of

age versus ≥65 years of age). The seasonal quadrivalent *high-dose* influenza vaccine (Groups 3 and 4) will only be administered to participants ≥65 years of age.

The previous SARS-CoV-2 vaccination history (Vaxzevria [AstraZeneca], Comirnaty [Pfizer-BioNTech], SpikeVax [Moderna], Ad26.COV2-S [Janssen] or COVID-19 vaccine naïve) will be used as second stratification factor. This stratification factor will aim to obtain a balanced distribution of SARS-CoV-2 vaccination history across the 4 treatment groups.

Participants can either have received a complete primary vaccination with an authorized/licensed COVID-19[a] vaccine (completed ≥6 months prior to first study vaccination) or be COVID-19 vaccine-naïve. Type of primary vaccination will be documented and accounted for according to interactive web response system (IWRS) procedures.

A diagram of the study design is provided in Section 1.2.

Study Duration

The study duration from screening until the last follow-up visit will be approximately 7-8 months. The study comprises screening on Day -28 to 1, vaccination visits on Days 1 and 29 with a 28-day follow-up period after each vaccination. AEs leading to withdrawal, MAAEs, SAEs, and AESIs will be collected throughout the study.

If a participant is unable to complete the study (post-vaccination 1), but has not withdrawn consent, an early exit visit will be conducted, or the participant can continue with other study procedures. The end of the study is defined as the last participant's last visit.

Unscheduled study visits may be performed based on investigator's clinical judgment and may include further evaluations, as needed.

Study Procedures

For each group, safety will be assessed by collection of solicited local (at injection site) and systemic AEs, unsolicited AEs, MAAEs, AESIs, and SAEs. Other safety assessments include vital signs measurements (heart rate, supine systolic and diastolic blood pressure, respiratory rate, and body temperature) and physical examinations at the time points indicated in the Schedule of Activities.

After each vaccination, participants will remain under observation at the study site for at least 15 minutes for presence of any acute reactions and solicited events. Any solicited local or systemic AEs, unsolicited AEs, MAAEs, AESIs, SAEs, concomitant medications, and vital signs will be documented by study-site personnel following this observation period. In addition, participants

[a] The following authorized/licensed COVID-19 vaccines are permitted in this study: Vaxzevria (AstraZeneca), Comirnaty (Pfizer-BioNTech), SpikeVax (Moderna), and Ad26.COV2-S (Janssen).

will record solicited signs and symptoms in a diary for 7 days post-vaccination (or until resolution) (Section 8).

The reporting periods of AEs, MAAEs, AESIs, and SAEs, and special reporting situations are detailed in Section 8.3. Reporting periods for concomitant therapy are outlined in Section 6.8. At each visit, the participant will be asked if they have had a private/off-study COVID-19 diagnostic test. See Section 8.3.1 on the reporting periods of this event.

Blood samples will be collected for assessment of humoral immune responses at the time points indicated in the Schedule of Activities.

Over the entire study, the total blood volume to be collected from each participant will be approximately 90 mL.

4.2. Scientific Rationale for Study Design

For the rationale for performing this study, refer to Section 2.1.

Dose Level Selection

The rationale behind the selection of the dose level is described in Section 4.3.

Blinding, Control, Study Phase/Periods, Vaccination Groups

Randomization will be used to minimize bias in the assignment of participants to vaccination groups, to increase the likelihood that known and unknown participant attributes (eg, demographic and baseline characteristics) are evenly balanced across vaccination groups, and to enhance the validity of statistical comparisons across vaccination groups. Blinded study vaccine will be used to reduce potential bias during data collection and evaluation of clinical endpoints.

4.2.1. Study-Specific Ethical Design Considerations

Potential participants will be fully informed of the risks and requirements of the study and, during the study, participants will be given any new information that may affect their decision to continue participation. They will be told that their consent to participate in the study is voluntary and may be withdrawn at any time with no reason given and without penalty or loss of benefits to which they would otherwise be entitled. Only participants who are fully able to understand the risks, benefits, and potential AEs of the study, and provide their consent voluntarily will be enrolled.

The study will be performed in adult participants who will receive vaccination against SARS-CoV-2 and influenza and compensation for their time and any inconveniences. See Section 2.3 for details on potential and known benefits and risks, and for the safety measures taken to minimize risk to participants.

The total blood volume to be collected is acceptable according to the US Department of Health and Human Services Office for Human Research Protections, and FDA guidelines (FDA 1998; US DHHS 1998), and the European Commission guidelines (EC 1998).

4.3. Justification for Dose

The 5×10^{10} vp dose level used in this study is consistent with the dose level authorized for emergency use in the United States (US) on 27 February 2021 and conditional marketing in the EU on 11 March 2021 (see Section 2).

4.4. End of Study Definition

End of Study Definition

The end of the study is considered as the last visit for the last participant in the study. The final data from the study site will be sent to the sponsor (or designee) after completion of the final participant visit at that study site, in the time frame specified in the Clinical Trial Agreement.

Study Completion Definition

A participant will be considered to have completed the study if the participant has completed assessments at 6-month follow-up visit post-vaccination 2.

Participants who prematurely discontinue the study vaccine for any reason before the second vaccination will not be considered to have completed the study. The reasons for such cases should be documented and available to the sponsor.

5. STUDY POPULATION

Screening for eligible participants could be performed within 28 days before the administration of the study vaccine. Refer to Section 5.4, Screen Failures for conditions under which the repeat of any screening procedures are allowed.

The inclusion and exclusion criteria for enrolling participants in this study are described below. If there is a question about these criteria, the investigator must consult with the appropriate sponsor representative and resolve any issues before enrolling a participant in the study. Waivers are not allowed.

For a discussion of the statistical considerations of participant selection, refer to Section 9.2.

NOTE: Investigators should ensure that all study enrollment criteria have been met at screening. If a participant's clinical status changes (including any available laboratory results or receipt of additional medical records) after screening but before the first dose of study vaccination and they no longer meet all eligibility criteria, the participant should be excluded from participation in the study (Refer to Section 5.4 for conditions for retesting).

The required source documentation to support meeting the enrollment criteria is noted in Section 10.3.10.

5.1. Inclusion Criteria

Each potential participant must satisfy all of the following criteria to be enrolled in the study:

1. Participant must sign an informed consent form (ICF) indicating that he or she understands the purpose, procedures, and potential risks and benefits of the study, and is willing to participate in the study.
2. Participant is willing and able to adhere to the prohibitions and restrictions specified in this protocol.
3. Participant is male or female aged ≥18 years of age, on the day of signing the ICF.
    a. Groups 3 and 4 only: Participant is male or female aged ≥65 years of age, on the day of signing the ICF.
4. Participant must be healthy, in the investigator’s clinical judgment, as confirmed by medical history, physical examination, and vital signs performed at screening. Participants may have underlying illnesses, as long as the symptoms and signs are medically controlled.
5. Criterion modified per Amendment 1

    5.1 Participant either received complete primary vaccination with an authorized/licensed COVID-19 vaccine (completed ≥6 months prior to the last vaccination received against COVID-19) or is COVID19 -vaccine-naïve.

    Note: The following authorized/licensed COVID-19 vaccines are permitted in this study: Vaxzevria (AstraZeneca), Comirnaty (Pfizer-BioNTech), SpikeVax (Moderna), and Ad26.COV2-S (Janssen).
6. Criterion modified per Amendment 1

    6.1 In the investigator’s clinical judgment, the participant may have a stable and well-controlled medical condition including comorbidities associated with an increased risk of progression to severe COVID-19 (CDC 2021a) (see Section 10.7, Appendix 7) (including stable/well-controlled HIV infection)*. If participants are on medication for a medical condition (including comorbidities associated with an increased risk of progression to severe COVID-19), the medication dose cannot have been modified within 4 weeks preceding vaccination. Participants will be included on the basis of relevant medical history at the investigator’s discretion.

    * Stable/well-controlled HIV infection includes:

    a. Documented CD4 cell count ≥300 cells/µL within 6 months prior to screening.

    b. Documented HIV viral load <50 copies/mL within 6 months prior to screening.

    c. Participant must be on a stable anti-retroviral treatment (ART) for 6 months (unless the change is due to tolerability, in which case the regimen can be for only the previous 3 months; changes in the formulation are allowed; nationwide guidelines that require transition from one ART regimen to another are allowed) and the participant must be willing to continue his/her ART throughout the study as directed by his/her local physician.

Note: Participants with ongoing and progressive comorbidities associated with HIV infection will be excluded but comorbidities associated with HIV infection that have been clinically stable for the past 6 months are not an exclusion criterion.

Laboratory methods for confirming a diagnosis of HIV infection are: Any evidence (historic or current) from medical records, such as ELISA with confirmation with Western Blot or real-time reverse-transcriptase polymerase chain reaction (RT-PCR), or of a detectable viral load (country-specific regulatory approved tests).

If a potential participant does not have HIV viral load and CD4 cell count data in his/her medical records from the last 6 months, they will be instructed to go to their local health care provider and obtain the necessary data for potential entry into the study. A laboratory result within 6 months of screening does not need to be repeated.

7. Contraceptive (birth control) use by participants should be consistent with local regulations regarding the acceptable methods of contraception for those participating in clinical studies.

   Before randomization, participants who were born female must be either (as defined in Section 10.5, Appendix 5):

   a. Not of childbearing potential

   b. Of childbearing potential and practicing a highly effective method of contraception and agrees to remain on such a method of contraception from signing the informed consent until 3 months after the administration of the last study vaccine. Use of hormonal contraception should start at least 28 days before the first administration of study vaccine. The investigator should evaluate the potential for contraceptive method failure (eg, noncompliance, recently initiated) in relationship to the first vaccination. Highly effective methods for this study include:

      1. hormonal contraception;

         a) combined (estrogen and progestogen containing) hormonal contraception associated with inhibition of ovulation (oral, intravaginal, or transdermal)

         b) progestogen-only hormonal contraception associated with inhibition of ovulation (oral, injectable, or implantable)

      2. intrauterine device;

      3. intrauterine hormone-releasing system;

      4. bilateral tubal occlusion/ligation procedure;

      5. vasectomized partner (the vasectomized partner should be the sole partner for that participant);

      6. sexual abstinence*.

      Sexual abstinence is considered an effective method* ***only* *if defined as refraining from heterosexual intercourse from signing the informed consent until 3 months after the last dose of study vaccine. The reliability of sexual abstinence needs to be evaluated in relation to the duration of the study and the preferred and usual lifestyle of the participant.*

8. Criterion modified per Amendment 1

   8.1 All participants who were born female and are of childbearing potential must:

   a. Have a negative highly sensitive urine pregnancy test at screening

b. Have a negative highly sensitive urine pregnancy test on the day of vaccination prior to each study vaccine administration.

9. Participant agrees to not donate or receive bone marrow, blood, and blood products from the administration of the study vaccine until 3 months after receiving the study vaccines.
10. Participant must be willing to provide verifiable identification to be contacted and to contact the investigator during the study.
11. Participant must be able to read, understand, and complete questionnaires in the participant's diary.
12. Participant must have access to a consistent means of contact either by telephone contact or e-mail/computer.

5.2. Exclusion Criteria

Any potential participant who meets any of the following criteria will be excluded from participating in the study:

1. Participant has a clinically significant acute illness (this does not include minor illnesses such as diarrhea or mild upper respiratory tract infection) or temperature ≥38.0ºC (100.4°F) within 24 hours prior to the planned dose of study vaccine; randomization at a later date is permitted at the discretion of the investigator.
2. Criterion modified per Amendment 1

   2.1 Participant has a history of malignancy within 1 year before screening (exceptions are squamous and basal cell carcinomas of the skin and carcinoma in situ of the cervix, or malignancies considered cured with minimal risk of recurrence per investigator's clinical judgment).
3. Participant has a known allergy or history of anaphylaxis or other serious adverse reactions to vaccines or their excipients (including specifically the excipients of the study vaccine) (refer to IB Ad26.COV2.S 2021 and its addenda for further details).
4. Participant has a history of severe allergic reactions (eg, anaphylaxis) to any component of the seasonal quadrivalent influenza vaccines, including egg protein, or following a previous dose of any influenza vaccine.
5. Criterion modified per Amendment 1

   5.1 Participant has an abnormal function of the immune system resulting from:

   a. Clinical conditions (eg, autoimmune disease or immunodeficiency) are expected to have an impact on the immune response elicited by the study vaccine. Participants with autoimmune diseases (eg, autoimmune thyroiditis, autoimmune inflammatory rheumatic diseases such as rheumatoid arthritis and type 1 diabetes) that are stable and controlled without the use of systemic immunomodulators and glucocorticoids may be enrolled at the discretion of the investigator.

      Note: Non-immunomodulatory treatment is allowed as well as steroids at a non-immunosuppressive dose or route of administration.

b. Chronic or recurrent use of systemic corticosteroids within 6 months before administration of the study vaccine and during the treatment period of the study at immunosuppressive doses.

   b.1 An immunosuppressive steroid dose is considered as >20 mg prednisone or equivalent daily for 2 consecutive weeks.
   Note: Ocular, topical, and inhaled steroids are allowed.

c. Administration of antineoplastic and immunomodulating agents or radiotherapy within 6 months before administration of the first study vaccine and during the treatment period of the study.

6. Participant has a history of any neurological disorders or seizures including Guillain-Barré syndrome, with the exception of febrile seizures during childhood.
7. Criterion deleted per Amendment 1
8. Participant received treatment with immunoglobulins within 3 months or exogenous blood products (autologous blood transfusions are not exclusionary) or blood products within 4 months before the administration of the first study vaccine or plans to receive such treatment during treatment period of the study.

   Note: The use of monoclonal antibodies (MABS) before administration of study vaccine and during the study conduct is permitted with the exception of those targeting T cells (anti-CD3, CD4, CD33, and CD52), B cells (anti-CD45, CD19, CD20, CD22, CD27, CD38, and CD138) and check point inhibitors (PD-1, PDL-1 and CTLA-4). Examples of not allowed MABS are: Muromonab-CD3, Gemtuzumab ozogamicin, Alemtuzumab, Bevacizumab, Rituximab, Ofatumumab, Ocrelizumab, Tositumomab, Veltuzumab, Obinutuzumab, Epratuzumab, Pembrolizumab, Nivolumab, Atezolizumab, avelumab, durvalumab and CTLA-4 targeting Ipilimumab. Participants receiving these MABS in the 2 months before the planned administration of the first study vaccine or who plan to receive these MABS during the treatment period of the study will be excluded from participating in the study.

9. Participant has history of TTS or heparin-induced thrombocytopenia and thrombosis (HITT).
10. Participant has history of capillary leak syndrome.
11. Participant received or plans to receive:

    a. Licensed live attenuated vaccines - within 28 days before or after planned administration of the first or subsequent study vaccines.

    b. Other licensed (not live) vaccines - within 14 days before or after planned administration of the first or subsequent study vaccines.

12. Criterion modified per Amendment 1

    12.1 Participant received a licensed/registered SARS-CoV-2 vaccine less than 6 months prior to first study vaccination or during the course of this study (other than study vaccination).

13. Participant received vaccination with a seasonal influenza vaccine for the current influenza season in the Northern Hemisphere.
14. Participant received an investigational drug (including any investigational agent for COVID-19 prophylaxis) or used an invasive investigational medical device within 30 days or received an investigational vaccine within 6 months before the administration of the study

vaccine or is currently enrolled or plans to participate in another investigational study during the course of this study.

Note: Participation in an observational clinical study (ie, without intervention) is allowed at the investigator's discretion.

15. Participant is a woman who is pregnant, or breastfeeding, or planning to become pregnant while enrolled in this study or within 3 months after the administration of the last study vaccine.
16. Participant has a history of an underlying clinically significant acute or chronic medical condition or physical examination findings for which, in the opinion of the investigator, participation would not be in the best interest of the participant (eg, compromise the well-being) or that could prevent, limit, or confound the protocol-specified assessments.
17. Participant had or plans to have major surgery (per the investigator's judgment) within 4 weeks before the administration of the first study vaccine or will not have recovered from surgery at the time of vaccination.
18. Participant has a contraindication to IM injections and blood draws (eg, bleeding disorders).
19. Participant is an employee of the investigator or study site, with direct involvement in the proposed study or other studies under the direction of that investigator or study site, as well as family members of the employees or the investigator, or an employee of the sponsor.
20. Participant has chronic active hepatitis B or hepatitis C infection per medical history.
21. Participant has had a major psychiatric illness or drug or alcohol abuse which in the investigator's opinion would compromise the participant's safety or compliance with the study procedures.
22. Participant cannot communicate reliably with the investigator.
23. Participant who, in the opinion of the investigator, is unlikely to adhere to the requirements of the study, or is unlikely to complete the full course of vaccination and observation.
24. Criterion modified per Amendment 1

    24.1 Participant has a positive diagnostic test result for current (viral RNA detection) SARS-CoV-2 infection prior to vaccine administration on Day 1.

NOTE: Investigators should ensure that all study enrollment criteria have been met prior to the first study vaccination. If a participant's clinical status changes (including any available laboratory results or receipt of additional medical records) after screening but before the first dose of study vaccination is given such that he or she no longer meets all eligibility criteria, then the participant should be excluded from participation in the study. Section 5.4 describes options for retesting. The required documentation to support meeting the enrollment criteria is described under Source Documents in Section 10.3.10, Appendix 3.

5.3. Lifestyle Considerations

Potential participants must be willing and able to adhere to the following lifestyle restrictions during the study to be eligible for participation:

1. Refer to Section 6.8 for details regarding prohibited and restricted therapy during the study.

2. Agree to follow all requirements that must be met during the study as noted in the Inclusion and Exclusion Criteria (eg, contraceptive requirements).

5.4. Screen Failures

Participant Identification, Enrollment, and Screening Logs

The investigator agrees to complete a participant identification and enrollment log to permit easy identification of each participant during and after the study. This document will be reviewed by the sponsor study site contact for completeness.

The participant identification and enrollment log will be treated as confidential and will be filed by the investigator in the study file. To ensure participant confidentiality, no copy will be made. All reports and communications relating to the study will identify participants by participant identification and age at initial informed consent. In cases where the participant is not randomized into the study, the date and age at initial informed consent will be used.

Individuals who do not meet the criteria for participation in this study (screen failure) may be rescreened once. Participants who are rescreened will be assigned a new participant number, undergo the ICF process, and then restart a new screening phase.

5.5. Criteria for Temporarily Delaying Administration of Study Vaccine

The following events constitute a temporary contraindication to study vaccination:

- Clinically significant acute illness at the time of vaccination. This does not include minor illnesses, such as diarrhea or mild upper respiratory tract infection.
- Fever (body temperature ≥38.0°C/100.4°F) within 24 hours prior to the planned time of vaccination.

If any of these events occur at the scheduled time for the first vaccination, randomization at a later date within the screening window is permitted at the discretion of the investigator and the reason should be documented. If any of these events occur at the scheduled time for one of the subsequent vaccinations, the vaccination visit can be rescheduled, within the allowed windows (see Visit Windows in the Schedule of Activities).

If the vaccination visit cannot be rescheduled within the allowed window or the contraindications to vaccination persist, the reason should be documented.

6. STUDY VACCINATION AND CONCOMITANT THERAPY

6.1. Study Vaccines Administered

Ad26.COV2.S will be supplied at a concentration of 1×10^{11} vp/mL in single-use vials, with an extractable volume of 0.5 mL, and dosed at 5×10^{10} vp. Placebo (0.9% sodium chloride [NaCl]) will be supplied in single-use vials.

The seasonal influenza vaccines to be used in this study are Afluria Quadrivalent (*standard-dose*) and Fluzone HD Quadrivalent (*high-dose*) or equivalent formulated for the 2021-2022 influenza- season:

- Afluria Quadrivalent (*standard-dose*) is a suspension for IM injection supplied in a 0.5-mL single-dose prefilled syringe. Each dose of Afluria Quadrivalent contains 60 µg of hemagglutinin (HA): 15 µg of each of 4 influenza strains (2 influenza A strains and 2 influenza B strains). For details, refer to the Afluria Quadrivalent package insert.
- Fluzone HD Quadrivalent (*high-dose*) is a suspension for IM injection supplied in a 0.7-mL single-dose prefilled syringe. Each dose of Fluzone HD Quadrivalent contains 240 µg of HA: 60 µg of each of 4 influenza strains (2 influenza A strains and 2 influenza B strains). For details, refer to the Fluzone HD Quadrivalent package insert.

Participants will be vaccinated at the study site according to the schedule shown in Table 2. Study vaccines will be administered by IM injection into the deltoid muscle. On Day 1, each participant will receive 2 IM injections. Each injection should be administered in opposite arms. It is recommended to use the right arm for the seasonal influenza vaccine on Day 1 and the left arm for the Ad26.COV2.S vaccine or placebo on Days 1 and 29.

On Day 29, each participant will receive 1 IM injection, preferably the deltoid muscle of the non-dominant upper arm is used.

If an injection cannot be given in the deltoids due to a medical or other contraindication (for example, tattooed upper arms rendering it difficult to assess site reactogenicity), use alternative locations such as the hip, thigh, or buttocks (to be avoided in overweight participants). When selecting an alternative location, the participant's ability to assess injection site events should be considered. In all circumstances, IM injections in other locations than the upper arm are not considered protocol deviations.

For further information on visit windows see the Schedule of Activities in Section 1.3.1. If a participant cannot be vaccinated within the allowed window, the decision regarding vaccination will be assessed on a case-by-case basis upon discussion between sponsor and investigator. The reason should be documented.

Study vaccine administration must be captured in the electronic case report form (eCRF).

Ad26.COV2.S and placebo will be manufactured and provided under the responsibility of the sponsor. Refer to the IB for a list of excipients (IB Ad26.COV2.S 2021).

Refer to the study site investigational product and procedures manual (SIPPM) and the Investigational Product Preparation Instructions (IPPI) for additional guidance on study vaccine administration.

Table 3: Description of Interventions

Intervention Name	Ad26.COV2.S	Placebo	Afluria Quadrivalent	Fluzone HD Quadrivalent
**Type**	Biologic/vaccine	Placebo	Biologic/vaccine	Biologic/vaccine
**Dose Formulation**	Single-use vials, with an extractable volume of 0.5 mL	Single-use vials, with an extractable volume of 10 mL	0.5 mL single-dose prefilled syringes	0.7 mL single-dose prefilled syringe.
**Unit Dose Strength(s)**	Ad26.COV2.S at a concentration of 1×10^{11} vp/mL	0.9% NaCl	15 µg HA of each influenza strain	60 µg HA of each influenza strain
**Dosage Level(s)**	Ad26.COV2.S 5×10^{10} vp once	0.5 mL once	60 µg HA once	240 µg HA once
**Route of Administration**	IM injection	IM injection	IM injection	IM injection
**Use**	Experimental	Placebo-comparator	Co-administration	Co-administration
**Investigational Medicinal Product (IMP)**	Yes	Yes	Yes	Yes
**Non-Investigational Medicinal Product/Auxiliary Medicinal Product (NIMP/AxMP)**	No	No	No	No
**Sourcing**	Provided centrally by the Sponsor			
**Packaging and Labeling** (Labels will contain information to meet the applicable regulatory requirements.)	The study vaccines will be packaged and labeled according to good manufacturing practices and local regulations. The study vaccines will not be packed in individual participant kits, 1 kit will be used by multiple participants. Each kit will contain single-use vials.		The study vaccines will be packaged and labeled according to good manufacturing practices and local regulations. The study vaccines will not be packed in individual participant kits, 1 kit will be used by multiple participants. Each kit will contain single-use vials or prefilled syringes.	
	Not in child resistant packaging			

HA = hemagglutinin; HD = high-dose; IM = intramuscular; vp = virus particles.

6.1.1. Combination Products

For this protocol, the term “combination product” refers to the single integral drug-device combination. The seasonal influenza vaccines administered in this study, Afluria Quadrivalent (standard-dose) and Fluzone HD Quadrivalent (high-dose), are considered combination products.

All combination product deficiencies (including failure, malfunction, improper or inadequate design, manufacturer error, use error, and inadequate labeling) shall be documented and reported by the investigator throughout the study. For studies using a combination product, these deficiencies will be reported as product quality complaints (PQC) (see Section 10.4.6; Appendix 4.6: Product Quality Complaint Handling) and appropriately managed by the sponsor.

6.2. Preparation/Handling/Storage/Accountability

Preparation/Handling/Storage

All study vaccine must be stored in a secured location with no access for unauthorized personnel and at controlled temperatures as indicated on the clinical labels. If study vaccine is exposed to temperatures outside the specified temperature range, all relevant data will be sent to the sponsor to determine if the affected supplies can be used or will be replaced. The affected study vaccine must be quarantined and not used until further instruction from the sponsor is received.

Refer to the SIPPM and the IPPI for additional guidance on study vaccine preparation, handling, and storage.

An unblinded study site pharmacist, or another qualified individual, who will have no other study function following vaccination, will prepare the appropriate vials and syringes, labeled with the participant’s identification number, and provide the syringes for the study vaccine in a blinded manner to the blinded vaccine administrator (a trained and qualified study nurse, medical doctor, or otherwise qualified health care professional [HCP]) who will perform the injection.

Accountability

The investigator is responsible for ensuring that all study vaccines received at the site are inventoried and accounted for throughout the study. The study vaccines administered to the participant must be documented on the vaccine accountability form. All study vaccines will be stored and disposed of according to the sponsor's instructions. Study site personnel must not combine the contents of the study vaccine containers.

Study vaccines must be handled in strict accordance with the protocol and the container label and must be stored at the study site in a limited-access area or in a locked cabinet under appropriate environmental conditions. Unused study vaccines must be available for verification by the sponsor's study site monitor during on-site monitoring visits. The return to the sponsor of unused study vaccines will be documented on the vaccine accountability form. When the study site is an authorized destruction unit and study vaccine supplies are destroyed on-site, this must also be documented on the vaccine accountability form.

Potentially hazardous materials containing hazardous liquids, such as needles and syringes should be disposed of immediately safely and therefore will not be retained for vaccine accountability purposes.

Study vaccines should be dispensed under the supervision of the investigator or a qualified member of the study site personnel, or by a hospital/clinic pharmacist. Study vaccines will be supplied only to participants participating in the study. Returned study vaccines must not be dispensed again, even to the same participant. Study vaccines may not be relabeled or reassigned for use by other participants. The investigator agrees neither to dispense the study vaccines from nor store them at, any site other than the study sites agreed upon with the sponsor. Further guidance and information for the final disposition of unused study vaccines are provided in the SIPPM.

6.3. Measures to Minimize Bias: Randomization and Blinding

Vaccine Allocation

Procedures for Randomization and Stratification

Central randomization will be implemented in this study. Participants will be randomly assigned to 1 of 2 vaccination groups (1:1 ratio; for participants ≥18 to ≤64 years of age) or 1 of 4 vaccination groups (1:1:1:1 ratio; for participants ≥65 years of age). This will be based on a computer-generated randomization schedule prepared before the study by or under the supervision of the sponsor.

The randomization will be balanced by using randomly permuted blocks and will be stratified by age (≥18 to ≤64 years of age versus ≥65 years of age) and previous SARS-CoV-2 vaccination history (Vaxzevria [AstraZeneca], Comirnaty [Pfizer-BioNTech], SpikeVax [Moderna], Ad26.COV2-S [Janssen], or COVID-19 vaccine naïve). The type of primary vaccination will be documented and accounted for according to IWRS procedures.

The IWRS will assign a unique intervention code, which will dictate the intervention assignment and matching study intervention kit for the participant. The requestor must use his or her own user identification and personal identification number when contacting the IWRS, and will then give the relevant participant details to uniquely identify the participant.

Blinding

Blinding will be guaranteed by the preparation of the study vaccine by an unblinded pharmacist or other qualified study site personnel with primary responsibility for study vaccine preparation and dispensing and by the administration of the vaccine in a masked syringe by a blinded study vaccine administrator. Participants will be randomly assigned to 1 of the groups based on a computer-generated randomization schedule prepared before the study by or under the supervision of the sponsor and using the IWRS.

The investigator will not be provided with randomization codes. The codes will be maintained within the IWRS, which has the functionality to allow the investigator to break the blind for an individual participant.

Data that may potentially unblind the intervention assignment (ie, immunogenicity data, study vaccine accountability data, study vaccine allocation, or other specific laboratory data) will be handled with special care to ensure that the integrity of the blind is maintained and the potential for bias is minimized. This can include making special provisions, such as segregating the data in question from view by the investigators, clinical team, or others as appropriate until the time of database lock and unblinding.

Under normal circumstances, the blind should not be broken until all participants have completed the treatment phase of the study and the database lock is completed. The investigator may, in an emergency, determine the identity of the intervention by contacting the IWRS. While the responsibility to break the intervention code in emergency situations resides with the investigator, it is recommended that the investigator contacts the sponsor or its designee if possible, to discuss the particular situation, before breaking the blind. Telephone contact with the sponsor or its designee will be available.

In the event the blind is broken, the sponsor must be informed as soon as possible. The date, time, and reason for the unblinding must be documented by the IWRS, electronic data capture (eDC) tool, and in the source document. The documentation received from the IWRS indicating the code break must be retained with the participant's source documents in a secure manner.

Participants who have had their intervention assignment unblinded should continue to return for scheduled evaluations.

Participants who withdraw will not be replaced.

6.4. Study Vaccination Compliance

Study vaccines will be administered IM by blinded qualified study site personnel at the study site. The date and time of study vaccine administration and the location used will be recorded in the eCRF.

6.5. Dose Modification

Dose modification is not applicable in this study.

6.6. Access to Licensed/Authorized Study Vaccine After the End of the Study

All participants in this study will receive Ad26.COV2.S vaccine and a seasonal quadrivalent influenza vaccine, irrespective of the study group to which they are randomized. Participants will not be offered another active study vaccine after the end of the study.

Participants will be instructed that a study vaccine will not be made available to them after they have completed/discontinued the study vaccine.

6.7. Treatment of Overdose

For this study, any dose of Ad26.COV2.S or of a seasonal quadrivalent influenza vaccine greater than the protocol-specified dose will be considered an overdose. The sponsor does not recommend specific treatment for an overdose.

In the event of an overdose, the investigator or treating physician should:

- Contact the sponsor or Medical Monitor immediately.
- Closely monitor the participant for AEs/AESIs/SAEs/MAAEs (ie, the participant will remain at the study site for at least 1 hour and will be closely monitored for allergic or other reactions by the study staff. Follow-up telephone calls 12 hours and 24 hours post-dose will be made).
- Document the quantity of the excess dose in the eCRF.
- Report as a special reporting situation (see Appendix 10.4.4).

6.8. Concomitant Therapy

Prestudy specific therapies such as analgesic/antipyretic medications and non-steroidal anti-inflammatory drugs, corticosteroids, antihistamines, and vaccinations taken up to 30 days before the first dose of study vaccine must be recorded at screening.

Concomitant therapies such as analgesic/antipyretic medications and non-steroidal anti-inflammatory drugs, corticosteroids, antihistamines, and vaccinations must be recorded (including dose and frequency) from the first dose of study vaccine until 28 days after administration of the last study vaccine.

All other concomitant therapies should also be recorded if administered in conjunction with a confirmed COVID-19 case or new or worsening AEs reported per protocol requirements outlined in Section 8.3.1.

For all participants, concomitant therapies associated with an SAE or suspected AESI meeting the criteria outlined in Sections 10.4.1 and 8.3.6, respectively, will be collected and recorded in the eCRF from the moment of first vaccination through the end of the study. Concomitant therapies associated with MAAEs or MAAEs leading to study discontinuation will be collected and recorded in the eCRF throughout the study.

For all participants, concomitant therapies associated with unsolicited AEs will be collected and recorded in the eCRF from the time of first vaccination through 28 days after the last vaccination. Concomitant therapies associated with solicited AEs will be collected by the participants and recorded in the eCRF from the time of each vaccination through 7 days after that vaccination (or until resolution).

Use of any experimental medication (including experimental vaccines other than the study vaccine) during the study is not allowed. Participants may not 1) receive an investigational drug (including investigational drugs for prophylaxis of COVID-19); 2) use of an invasive investigational medical device within 30 days; 3) receive an investigational immunoglobulins or

monoclonal antibodies within 3 months; 4) receive convalescent serum for COVID-19 treatment within 4 months; 5) receive an investigational vaccine within 6 months before the administration of the first study vaccine.

Vaccination with licensed live attenuated vaccines within 28 days of a study vaccination (ie, before or after) is prohibited. Other licensed (not live) vaccines (eg, tetanus, hepatitis A, hepatitis B, rabies) should be given at least 14 days before (or at least 14 days after) administration of the study vaccine to avoid potential confusion of adverse reactions and potential immune interference.

The use of any SARS-CoV-2 vaccine (licensed/authorized or investigational) other than study vaccination is disallowed less than 6 months prior to the first study vaccination and during the course of the study. In the event that a participant does receive another COVID-19 vaccine outside of the study, investigators are required to enter any COVID-19 vaccines (name/manufacturer of the vaccine and date of administration) in the Concomitant Therapy eCRF.

Vaccination with a seasonal influenza vaccine for the 2021-2022 influenza season in the Northern Hemisphere is disallowed at any time prior to vaccination and during the course of the study. If a vaccine is indicated in a post-exposure setting (eg, rabies or tetanus), it could take priority over the study vaccine and must be documented in the Concomitant Therapy eCRF.

Antipyretics are recommended post-vaccination for symptom relief as needed. Prophylactic antipyretic use is not encouraged; however, in some instances, it could be considered in special circumstances and/or comorbidities per the investigator’s discretion.

Chronic or recurrent use of systemic corticosteroids[a] at immunosuppressive dose (defined as >20 mg prednisone or equivalent daily for 2 consecutive weeks) and administration of antineoplastic and immunomodulating agents or radiotherapy is prohibited during the treatment phase (ie, through 1 month post vaccination 2). If any of these agents are indicated in a disease setting, medical judgment should prevail.

Treatment with immunoglobulins within 3 months or exogenous blood products (autologous blood transfusions are not exclusionary) or blood products within 4 months before the administration of the study vaccine or during the treatment period of the study is disallowed.

Refer to Section 5.2 for further details on time frames for prohibited therapy.

The sponsor must be notified as soon as possible of any instances in which prohibited therapies are administered.

[a] Note: Ocular, topical, or inhaled steroids are allowed.

7. DISCONTINUATION OF STUDY VACCINATION AND PARTICIPANT DISCONTINUATION/WITHDRAWAL

7.1. Discontinuation of Study Vaccination

Study vaccinations will be withheld for the reasons listed below. These participants must not receive any further doses of the study vaccine and could opt for an early termination visit or remain on study for follow-up with assessments of safety and immunogenicity as indicated in the Schedule of Activities. Additional unscheduled visits may be performed for safety/reactogenicity reasons, if needed. In case of questions, the investigator is encouraged to contact the sponsor.

- Any related AE, worsening of health status, or intercurrent illnesses that, in the opinion of the investigator, requires discontinuation from study vaccine
- Unblinding on the participant level that, in the opinion of the sponsor, would compromise the integrity of the data
- Anaphylactic reaction following vaccination, not attributable to causes other than vaccination
- SAE or other potentially life-threatening (Grade 4) event that is determined to be related to study vaccine
- Chronic or recurrent use of systemic corticosteroids at immunosuppressive dose (see Section 6.8), and administration of antineoplastic and immunomodulating agents or radiotherapy
- Withdrawal of consent to receive further study vaccination
- Participant received any experimental medication (including experimental vaccines other than the study vaccine) or receives a licensed/registered COVID-19 vaccine or treatment
- Participant received an influenza vaccine (other than the one administered in the study) for the current influenza season in the Northern Hemisphere
- Participant previously experienced TTS, including CVST or heparin-induced thrombocytopenia (HIT)
- Participant experiences capillary leakage syndrome after the first dose

7.2. Participant Discontinuation/Withdrawal From the Study

A participant will be withdrawn from the study for any of the following reasons:

- Lost to follow-up
- Withdrawal of consent from the study
- Death
- Repeated failure to comply with protocol requirements

When a participant withdraws before study completion, the reason for withdrawal is to be documented in the eCRF and in the source document. If the reason for withdrawal from the study

is the withdrawal of consent, then no additional assessments are allowed, but an optional safety visit could be offered.

Withdrawal of Consent

A participant declining to return for scheduled visits does not necessarily constitute withdrawal of consent. Alternate follow-up mechanisms that the participant agreed to when signing the consent form apply as local regulations permit.

7.2.1. Withdrawal From the Use of Research Samples

Withdrawal From the Use of Samples in Future Research

The participant may withdraw consent for use of samples for research (refer to Long-Term Retention of Samples for Additional Future Research in Section 10.3.5, Appendix 3). In such a case, samples will be destroyed after they are no longer needed for the clinical study. Details of the sample retention for research are presented in the main ICF.

7.3. Lost to Follow-up

To reduce the chances of a participant being deemed lost to follow-up, prior to randomization attempts should be made to obtain contact information from each participant, eg, home, work, and mobile telephone numbers and e-mail addresses for both the participant as well as appropriate family members.

A participant will be considered lost to follow-up if the participant repeatedly fails to return for scheduled visits and is unable to be contacted by the study site after multiple documented attempts. A participant cannot be deemed lost to follow-up until all reasonable efforts made by the study site personnel to contact the participant are deemed futile.

The following actions must be taken if a participant fails to return to the study site for a required study visit:

- The study site personnel must attempt to contact the participant to reschedule the missed visit as soon as possible, to counsel the participant on the importance of maintaining the assigned visit schedule, to ascertain whether the participant wishes to or should continue in the study.
- Before a participant is deemed lost to follow-up, the investigator or designee must make every reasonable effort to regain contact with the participant (where possible, 3 telephone calls, emails, fax, and, if necessary, a certified letter to the participant's last known mailing address, or local equivalent methods). Locator agencies may also be used as local regulations permit. These contact attempts should be documented in the participant's medical records.
- Should the participant continue to be unreachable, they will be considered to have withdrawn from the study.

Should a study site close, eg, for operational, financial, or other reasons, and the investigator cannot reach the participant to inform them, their contact information will be transferred to another study site.

8. STUDY ASSESSMENTS AND PROCEDURES

Overview

The Schedule of Activities summarizes the frequency and timing of safety, reactogenicity, immunogenicity, and all other measurements applicable to this study.

If multiple assessments are scheduled for the same timepoint, it is recommended that procedures be performed in the following sequence: vital signs and physical examination before blood draws. If needed, assessments may be performed on another day within the applicable visit window. Actual dates and times of assessments must be recorded in the source document and the eCRF.

Participants will be provided a thermometer (to measure body temperature), ruler (to measure local injection site reactions), and an electronic diary to record body temperature and solicited local (at injection site) and systemic symptoms. The diary includes instructions on how to capture the data and grading scales to assess severity of the signs and symptoms post-vaccination (reactogenicity).

The study staff is responsible for providing appropriate training to the participant to avoid missing or incorrect data. The diary will be reviewed by the study personnel on an ongoing basis and the investigator's assessment of reactogenicity will be completed on the 7-day post vaccination visit as indicated in the Schedule of Activities in Section 1.3.1). If there are ongoing symptoms, the diary can be reviewed during the next visit. If a participant misses a vaccination, the diary covering the period after the missed vaccination does not have to be completed.

The total blood volume to be collected from each participant throughout the study will be approximately 90 mL. Repeat or unscheduled samples may be taken for safety reasons or for technical issues with the samples. For participants who experience a suspected AESI, an additional approximately 15 mL of blood will be collected for additional safety assessments, as indicated in the Schedule of Activities in Section 1.3.2.

At each visit the participant will be asked if they have had a private/off-study COVID-19 test. If a participant receives a positive SARS-CoV-2 result from a private/off-study test (whether through PCR or serological testing), the event should be reported as medical history, AE or SAE if the event meets the criteria for an SAE. The participant can continue in the study in accordance with country and site level recommendations for COVID-19.

The study staff can recommend the participant informs their medical care provider. If there are no local country recommendations for COVID-19, the sponsor recommends the participant self-quarantines and does not come for a clinic visit until a negative PCR test could be obtained.

If the participant experiences signs or symptoms of COVID-19 after obtaining a positive SARS-CoV-2 test result, they should contact the study site at the time of symptom onset for

recommendations on actions to follow for further care per local guidance. See section 8.3.1 on the reporting periods of this event.

Visit Windows

Visit windows are provided in the Schedule of Activities in Section 1.3. The participant should be encouraged to come on the day planned, but the visit window may be used.

The timings of the post-vaccination visits will be determined relative to the actual day of the corresponding vaccination.

If a vaccination window is missed, efforts will be made to vaccinate the participant as soon as possible, even if out of the window. The timings of the post-vaccination visits will be determined relative to the actual day of the corresponding vaccination, unless they overlap with other scheduled visits, in which case the out of window visit should be documented in the source documents. If the participant refuses to receive the second vaccination, the investigator is encouraged to discuss it with the sponsor.

Screening

Screening may be performed within 28 days prior to the first study vaccination or on the day of vaccination. If screening is performed on the day of vaccination, Visit 1 and Visit 2 will coincide on Day 1. Screening must be completed and all eligibility criteria must be fulfilled prior to randomization and vaccination.

Screening may be conducted in part via a sponsor- and Independent Ethics Committee/Institutional Review Board (IRB/IEC)-pre-approved non-study-specific screening consent process, but only if the relevant pre-screening tests are identical to the per protocol screening tests and are within 4 weeks prior to first vaccination. However, no study-specific procedures, other than these pre-approved pre-screening assessments, will be performed until the participant has signed the study-specific ICF. The -study-specific signed ICF date must be entered into the eCRF.

Sample Collection and Handling

The actual dates and times of sample collection must be recorded in the eCRF or laboratory requisition form. Refer to the Schedule of Activities in Section 1.3 for the timing and frequency of all sample collections.

Instructions for the collection, handling, storage, and shipment of samples are found in the laboratory manual that will be provided. Collection, handling, storage, and shipment of samples must be under the specified, and where applicable, controlled temperature conditions as indicated in the laboratory manual.

Study-Specific Materials

The investigator will be provided with the following supplies:

- IB and any Addenda for Ad26.COV2.S

- Package insert for Ad26.COV2.S
- Package inserts for Afluria Quadrivalent and Fluzone HD Quadrivalent
- Thermometer
- Ruler (to measure diameter of any erythema and swelling)
- Pharmacy manual/IPPI/SIPPM
- Laboratory manual and laboratory supplies
- IWRS manual
- eCRF completion guidelines
- Sample ICF
- Participant diary/ Site training manual
- Contact information page(s)

8.1. Immunogenicity Assessments

No generally accepted immunological correlate of protection has been demonstrated for SARS-CoV-2 to date. If a correlate or threshold for protection against COVID-19 is established in terms of humoral immunity, then a statistical comparison to that correlate or threshold will substitute NI testing to immune responses to vaccine at release, as outlined in the study statistical analytical plan (SAP).

Venous blood samples will be collected for assessment of humoral immune responses. Sample volumes and time points are detailed in the Schedule of Activities in Section 1.3.

If the participant is unable to complete the study but they did not withdraw consent, immunogenicity samples will be taken at the early exit visit, but only if the early exit visit is at least 10 days after the previous immunology blood draw. See the Schedule of Activities for further details.

Humoral immunogenicity assays may include, but are not limited to, the assays summarized in Table 4.

Table 4: Summary of Humoral Immunogenicity Assays

Assay	Purpose
**Humoral Immunogenicity**	
Primary endpoints	
HI assay [a]	Analysis of HI to influenza strains
SARS-CoV-2 binding antibodies (ELISA)	Analysis of antibodies binding to SARS-CoV-2 or individual SARS-CoV-2 proteins (eg, S protein)
Secondary endpoints	
HI assay [a]	Analysis of HI to influenza strains
SARS-CoV-2 binding antibodies (ELISA)	Analysis of antibodies binding to SARS-CoV-2 or individual SARS-CoV-2 proteins (eg, S protein)
Exploratory endpoints	
HI assay	Analysis of HI to influenza strains

SARS-CoV-2 binding antibodies (ELISA)	Analysis of antibodies binding to SARS-CoV-2 or individual SARS-CoV-2 proteins (eg, S protein)
SARS-CoV-2 neutralization (VNA)	Analysis of neutralizing antibodies to the wild-type virus and/or pseudovirion expressing S protein
Adenovirus neutralization	Adenovirus 26 neutralization assay to evaluate neutralizing antibody responses against the Ad26 vector
SARS-CoV-2 binding antibodies (ELISA and/or SARS-CoV-2 immunoglobulin assay)	Analysis of antibodies binding to the SARS-CoV-2 N protein
Functional and molecular antibody characterization	Analysis of antibody characteristics including Fc-mediated viral clearance, avidity, Fc characteristics, Ig subclass and IgG isotype

ELISA = enzyme-linked immunosorbent assay; Fc = fragment crystallizable; HI assay = Hemagglutination inhibition (HI) assay; Ig = immunoglobulin; S = Spike; SARS-CoV2 = severe acute respiratory syndrome coronavirus-2; VNA = virus neutralization assay.

a. The HI antibody titers against influenza virus strains recommended by the World Health Organization (WHO) for use in 2021-2022 in the Northern Hemisphere season will be measured using a validated HI assay platform.

8.2. Safety Assessments

Adverse events will be reported and followed by the investigator as specified in Section 8.3 and Appendix 4 (Section 10.4).

Any clinically relevant changes occurring during the study period must be recorded on the AE section of the eCRF.

Any clinically significant abnormalities will be followed by the investigator until resolution or until a clinically stable condition is reached. For those abnormalities persisting at the end of the study/early withdrawal, the outcome should be documented in the participant's medical chart.

The study will include the following evaluations of safety and tolerability according to the time points provided in the Schedule of Activities.

8.2.1. Physical Examinations

A full physical examination, including height and body weight, will be carried out at screening.

At all other visits, an abbreviated, symptom-directed examination might be performed by the investigator based on any clinically relevant issues or symptoms, and medical history. Symptom-directed physical examination may be repeated if deemed necessary by the investigator.

Physical examinations will be performed by the investigator or designated medically trained clinician. Any clinically relevant abnormalities or changes in severity observed during the review of body systems should be documented in the eCRF.

8.2.2. Vital Signs

Body temperature (oral route preferred, or in accordance with the local standard of care), pulse/heart rate, respiratory rate, and blood pressure will be assessed.

Participants will utilize a diary to record body temperature measurements post-vaccination (see Section 8).

Blood pressure and pulse/heart rate measurements will be assessed preferably supine with a completely automated device. Manual techniques will be used only if an automated device is not available.

Blood pressure and pulse/heart rate measurements should be preceded by at least 5 minutes of rest in a quiet setting without distractions (eg, television, cell phones). Vital sign measurements are recommended before blood sampling.

8.2.3. Clinical Safety Laboratory Assessments

Blood samples for clinical laboratory assessments (as detailed in Section 10.2, Appendix 2) will be collected as described in the Schedule of Activities in Section 1.3.

In case of a thrombotic event, thrombocytopenia, or TTS occurring post-vaccination, every effort should be made to collect in a local hospital/laboratory the blood test results obtained to ensure that the treating physician performs a rapid diagnosis and treatment.

This information should be reported through the TTS AESI form (see Section 10.8, Appendix 8) electronically per instructions in the eCRF completion guidelines. In addition, every effort should be made to collect blood samples from the participant for a platelet count (local laboratory or substitute for local laboratory) and other applicable testing (central laboratory; eg, anti-PF4 see the Schedule of Activities in Section 1.3.2 and Section 10.2, Appendix 2).

The investigator will review the laboratory test results performed locally and investigate potential cases of thrombosis (per Appendix 9, Section 10.9) to assist the investigation of the AESI, and document it in eDC.

See Section 8.3.6.1 for details on laboratory tests to be reported for post-vaccination thrombocytopenia.

8.2.4. Pregnancy Testing

A urine pregnancy test for participants of childbearing potential will be performed at screening and before each vaccination.

Additional pregnancy tests may be performed for participants of childbearing potential, as determined necessary by the investigator or required by local regulation, to establish the absence of pregnancy at any time during the participation in the study.

8.3. Adverse Events, Serious Adverse Events, Medically-attended Adverse Events, Adverse Events of Special Interest, and Other Safety Reporting

Timely, accurate, and complete reporting and analysis of safety information, including AEs, SAEs, MAAEs, AESIs, and PQCs, from clinical studies are crucial for the protection of participants, investigators, and the sponsor, and are mandated by regulatory agencies worldwide.

The sponsor has established Standard Operating Procedures in conformity with regulatory requirements worldwide to ensure appropriate reporting of safety information; all clinical studies conducted by the sponsor or its affiliates will be conducted in accordance with those procedures.

Adverse events will be reported by the participant (or, when appropriate, by a caregiver, surrogate, or the participant's legally acceptable representative) for the duration of the study.

Further details on AEs, SAEs, MAAEs, AESIs, and PQCs can be found in Section 10.4, Appendix 4.

8.3.1. Time Period and Frequency for Collecting Adverse Event, Medically-attended Adverse Event, Adverse Event of Special Interest, and Serious Adverse Event Information

All Adverse Events

Adverse events and special reporting situations, whether serious or non-serious, that are related to study procedures or that are related to non-investigational sponsor products will be reported from the time a signed and dated ICF is obtained until the end of the study/early withdrawal.

Clinically relevant medical events not meeting the above criteria and occurring between signing of ICF and moment of first vaccination will be collected on the Medical History eCRF page as pre-existing conditions.

Solicited AEs, collected through a diary, will be recorded for each vaccination from the time of vaccination until 7 days post-vaccination (or until resolution).

All unsolicited AEs and special reporting situations, whether serious or non-serious, will be reported for each vaccination from the time of vaccination until 28 days post-vaccination. Unsolicited adverse events with the onset date outside the timeframe defined above (>28 days after each study vaccination), which are ongoing on the day of the subsequent vaccination, should be recorded as such.

At each visit the participant will be asked if they have had a private/off-study COVID-19 test. If a participant receives a positive SARS-CoV-2 result from a private/off-study test during the study period (whether through PCR or serological testing) the event should be reported as an AE or as an SAE if the event meets the criteria of an SAE. The participant can continue in the study in accordance with local country and site level recommendations for COVID-19. Any positive SARS-CoV-2 result should be recorded in the eCRF. Previous SARS-CoV-2 infection should be documented as medical history and does not preclude participation in the study.

All SAEs, AESIs, and AEs leading to discontinuation from the study/vaccination (regardless of the causal relationship) are to be reported from the moment of first vaccination until the end of the study, which may include contact for safety follow-up. The sponsor will evaluate any safety information that is spontaneously reported by an investigator beyond the time frame specified in the protocol.

MAAEs are defined as AEs with medically-attended visits including hospital, emergency room, urgent care clinic, or other visits to or from medical personnel for any reason. Routine medical visits for chronic comorbidities (or study visits) will not be considered medically-attended visits. New onset of chronic diseases will be collected as part of the MAAEs. MAAEs are to be reported for all participants throughout the study.

As long as the participant remains in the study, all AEs will be followed until resolved, until clinically stable or the end of the study, as applicable. The investigator's assessment of ongoing AEs at the time of each participant's last visit should be documented in the participant's medical chart.

Adverse Events of Special Interest

TTS is considered to be an AESI. Suspected AESIs (thrombotic events and thrombocytopenia [defined as platelet count below 150,000/µL (Brighton Collaboration 2021)] post-vaccination) will be recorded from the moment of vaccination until the end of the study/early withdrawal. An AESI Adjudication Committee with appropriate expertise will be established to evaluate each suspected AESI and determine whether it is a case of TTS (see Section 8.3.6).

All AESIs occurring during the study period must be reported to the sponsor by study site personnel within 24 hours of their knowledge of the event and complete the procedures indicated in the Schedule of Activities in Section 1.3.2.

Serious Adverse Events

All SAEs, as well as PQCs, occurring during the study must be reported to the appropriate sponsor contact person by study site personnel within 24 hours of their knowledge of the event.

Serious adverse events, including those spontaneously reported to the investigator, must be reported using an SAE form. The sponsor will evaluate any safety information that is spontaneously reported by an investigator beyond the time frame specified in the protocol.

Information regarding SAEs will be transmitted to the sponsor using the SAE Form and Safety Report Form of the CRF, which must be completed and reviewed by a physician from the study site, and transmitted to the sponsor within 24 hours. The initial and follow-up reports of an SAE should be transmitted electronically or by facsimile (fax). Telephone reporting should be the exception and the reporter should be asked to complete the appropriate form(s) first.

8.3.2. Method of Detecting Adverse Events, Medically-attended Adverse Events, Adverse Events of Special Interest, and Serious Adverse Events

Care will be taken not to introduce bias when detecting AEs, MAAEs, suspected AESIs, or SAEs. Open-ended and nonleading verbal questioning of the participant is the preferred method to inquire about AE occurrence.

Solicited Adverse Events

Solicited AEs are used to assess the reactogenicity of the study vaccine and are predefined local (at injection site) and systemic events for which the participant is specifically questioned and which are noted by participants in their diary.

After each vaccination, participants will remain under observation at the study site for at least 15 minutes for presence of any acute reactions and solicited events.

In addition, participants will record solicited signs and symptoms in a diary for 7 days post-vaccination (or until resolution). All participants will be provided with a diary and instructions on how to complete the diary (see Overview in Section 8).

Electronic diary information will be transferred from the e-diary source to the sponsor. After review and verbal discussion of the initial electronic diary entries with the participant, the investigator will complete his/her own assessment in the relevant sections of the CRF (ie, investigator's assessment of reactogenicity). Once a solicited symptom from a diary is verified and considered to be of severity Grade 1 or above, it will be recorded as a solicited adverse event per FDA toxicity grading (See Appendix 6, Section 10.6).

Solicited Injection Site (Local) Adverse Events

Participants will be asked to note in the diary occurrences of local reaction at the injection site namely pain/tenderness, erythema, swelling, and systemic reactions, namely fatigue, headache, nausea, and myalgia daily for 7 days post-vaccination (or until resolution, whatever happens first). The extent (largest diameter) of any erythema and swelling should be measured (using the ruler supplied) and recorded daily. The case definitions for solicited injection site events can be found in the references (Gidudu 2012; Kohl 2007).

Solicited Systemic Adverse Events

Participants will be instructed on how to record daily temperature using a thermometer for home use. Participants should record the temperature in the diary starting in the evening of the day of vaccination, and then daily for the next 7 days approximately at the same time each day. If more than one measurement is made on any given day, the highest temperature of that day should be recorded.

Fever is defined as endogenous elevation of body temperature ≥38°C (100.4°F). (See Appendix 6, Section 10.6.)

Unsolicited Adverse Events

Unsolicited AEs are all AEs for which the participant is not specifically questioned in the participant diary.

Medically-attended Adverse Events

MAAEs are AEs with medically-attended visits including hospital, emergency room, urgent care clinic, or other visits to or from medical personnel for any reason. New onset of chronic diseases

will be collected as part of the MAAEs. Routine study visits will not be considered medically-attended visits.

Adverse Events of Special Interest

For details about AESIs, refer to Section 8.3.6.

8.3.3. Follow-up of Adverse Events, Medically-attended Adverse Events, Adverse Events of Special Interest, and Serious Adverse Events

The investigator is obligated to perform or arrange for the conduct of supplemental measurements and evaluations as medically indicated to elucidate the nature and causality of the AE, MAAE, AESI, SAE, or PQC as fully as possible. This may include additional laboratory tests or investigations, histopathological examinations, or consultation with other health care professionals.

Adverse events, including pregnancy, will be followed by the investigator as specified in Section 10.4, Appendix 4.

8.3.4. Regulatory Reporting Requirements for Serious Adverse Events

The sponsor assumes responsibility for appropriate reporting of AEs to the regulatory authorities. The sponsor will also report to the investigator (and the head of the investigational institute where required) all suspected unexpected serious adverse reactions (SUSARs).

The investigator (or sponsor where required) must report SUSARs to the appropriate IEC/IRB that approved the protocol unless otherwise required and documented by the IEC/IRB. A SUSAR will be reported to regulatory authorities unblinded. Participating investigators and IEC/IRB will receive a blinded SUSAR summary, unless otherwise specified.

8.3.5. Pregnancy

All initial reports of pregnancy in participants or partners of male participants must be reported to the sponsor by the study site personnel within 24 hours of their knowledge of the event using the appropriate pregnancy notification form. Abnormal pregnancy outcomes (eg, spontaneous abortion, fetal death, stillbirth, congenital anomalies, ectopic pregnancy) are considered SAEs and must be reported using an SAE reporting form.

Any participant who becomes pregnant during the study may continue study vaccination if the investigator considers that the potential benefits outweigh any potential risks to the mother and fetus and is allowed by local regulations.

Follow-up information regarding the outcome of the pregnancy and any postnatal sequelae in the infant will be required.

8.3.6. Adverse Events of Special Interest

AESIs are significant AEs that are judged to be of special interest because of clinical importance, known or suspected class effects, or based on nonclinical signals. Adverse events of special interest will be carefully monitored during the study by the sponsor.

Suspected AESIs must be reported to the sponsor within 24 hours of awareness irrespective of seriousness (ie, serious and non-serious AEs) or causality following the procedure described above for SAEs.

At baseline, safety blood samples will be collected. Whole blood samples will be used for a complete blood counts (CBC), including platelet count (as part of a complete blood count), in a local laboratory or substitute for local laboratory, depending on local feasibility towards turnaround time of sample processing.

Serum and plasma samples will be collected and stored for potential future coagulation-related testing in a central laboratory if the participant experiences an AESI (see Section 10.2, Appendix 2).

Specific requirements for the AESI are described below.

8.3.6.1. Thrombosis with Thrombocytopenia Syndrome

As described in Section 2.3.1, TTS has been observed very rarely following vaccination with Ad26.COV2.S and is considered to be an AESI in this study. TTS is a syndrome characterized by a combination of both a thrombotic event and thrombocytopenia (American Society of Hematology 2021; Brighton Collaboration 2021).

Because this syndrome is rare and not completely understood, all cases of thrombosis and/or thrombocytopenia will be considered a suspected case of TTS until further adjudication can be performed. An AESI Adjudication Committee with appropriate expertise will be established to evaluate each suspected AESI and determine whether it is a case of TTS. The investigator shall be responsible for reporting any suspected AESI of TTS using the SAE form and the form detailed in Section 10.8, Appendix 8.

A suspected TTS case is defined as:

- Thrombotic events: any thrombotic or thromboembolic event as detailed in Section 10.9, Appendix 9
- Thrombocytopenia, defined as platelet count below 150,000/µL (Brighton Collaboration 2021) post-vaccination

Symptoms, signs, or conditions suggestive of a thrombotic event should be recorded and reported as a suspected AESI even if the final or definitive diagnosis has not yet been determined, and alternative diagnoses have not yet been eliminated or shown to be less likely. Follow-up information and final diagnoses, if applicable, should be submitted to the sponsor as soon as feasible.

In the event of post-vaccination thrombocytopenia, study site personnel should report the absolute value for the platelet count and the reference range for the laboratory test used in the eCRF. In the event of thrombocytopenia post study vaccination, thrombotic events should be excluded (through laboratory tests or imaging).

For either a thrombotic event or thrombocytopenia, testing for anti-PF4 should be performed at the central laboratory or substitute local laboratory; repeat testing may be requested for confirmation upon sponsor discretion.

Suspected AESIs will require enhanced data collection and evaluation (see Section 1.3.2). Every effort should be made to report as much information as possible about the AESI to the sponsor in a reasonable timeframe

If an event meets the criteria for an SAE (Section 10.4.1), it should be reported using the same process as for other SAEs.

The form detailed in Section 10.8, Appendix 8 is intended as a guide for assessment of the AESIs to facilitate diagnosis and determine treatment options. If the investigator is not the treating physician, every effort should be made to collect the information requested in the form from the treating physician and enter the available information in the eCRF.

9. STATISTICAL CONSIDERATIONS

Statistical analysis will be done by the sponsor or under the authority of the sponsor. A general description of the statistical methods to be used to analyze the efficacy and safety data is outlined below. Specific details will be provided in the SAP.

9.1. Statistical Hypotheses

The study is successful if the below objectives are demonstrated:

- NI of the humoral immune response as measured by the HI assay after concomitant administration of Ad26.COV2.S vaccine and a seasonal *standard-dose* influenza vaccine versus the administration of a seasonal quadrivalent *standard-dose* influenza vaccine alone, 28 days after the administration of a seasonal influenza vaccine.

  AND

- NI of the concomitant administration of Ad26.COV2.S vaccine and a seasonal quadrivalent *standard-dose* influenza vaccine versus the administration of Ad26.COV2.S vaccine alone, 28 days after the administration of Ad26.COV2.S vaccine, in terms of humoral immune response (ELISA)

Therefore, the following CIs will be calculated:

- The 2-sided 95% CIs for the difference in log-transformed HI antibody titers against each of the 4 seasonal influenza vaccine strains at 28 days after the administration of the quadrivalent seasonal influenza vaccine between the Control and the CoAd group, and

- The 2-sided 95% CIs for the difference in log-transformed ELISA titers at 28 days after the administration of Ad26.COV2.S vaccine between the Control and the CoAd group

Only if the upper bound of the 2-sided 95% CI for the GMT ratio (Control group/CoAd group) of the HI antibody titer for each of the 4 vaccine strains and for GMC for ELISA lies below 1.5, NI of co-administration versus separate administration will be concluded for both vaccines (seasonal influenza vaccine and Ad26.COV2.S vaccine). If 1 or more confidence limits for the GMT/GMC ratio exceed 1.5, NI cannot be concluded.

The primary analysis will be performed when all participants have completed the 28 days after the second study vaccination visit or discontinued earlier. No interim analyses will be performed.

For the high-dose groups, no formal hypothesis testing is planned.

No formal statistical testing of safety data is planned. Safety data will be analyzed descriptively by vaccine group.

9.2. Sample Size Determination

Sample size calculations are performed under the following assumptions:

- no effect of co-administration of Ad26.COV2.S vaccine and seasonal influenza vaccine on the immune response against influenza as measured by HI antibody titers against the 4 influenza vaccine strains at 28 days after the administration of seasonal influenza vaccine
- the use of Afluria Quadrivalent for *standard-dose* (adults ≥18 to <64 years of age and older adults ≥65 years of age)
- a standard deviation of 0.53 for the *standard-dose* at the $\log_{10}$ scale for HI antibody titers against the 4 influenza vaccine strains at 28 days after the administration of seasonal influenza vaccine (with or without Ad26.COV2.S)
- no effect of co-administration of Ad26.COV2.S vaccine and seasonal influenza vaccine on the immune response against SARS-CoV-2 as measured by S-ELISA at 28 days after the administration of Ad26.COV2.S vaccine
- a standard deviation of 0.50 at the $\log_{10}$ scale for S-ELISA at 28 days after the administration of Ad26.COV2.S vaccine (with or without seasonal influenza vaccine)
- a NI margin of 1.5
- 2-sided α of 5%

A total of approximately 305 (*standard-dose*) participants per group (total N 610) who have completed a primary COVID-19 vaccine series are needed to have 97.45% power to show NI in HI antibody titers for 1 influenza vaccine strain.

The sample size accounts for exclusions from the per protocol set (see Section 9.3), drop-outs and missing samples.

With this sample size, the overall power to show NI in HI antibody titers against each of the 4 influenza vaccine strains at 28 days after the administration of seasonal influenza vaccine as well as NI in S-ELISA at 28 days after the administration of Ad26.COV2.S vaccine is at least 90%.

9.3. Populations for Analysis Sets

For purposes of analysis, the following populations are defined:

Full Analysis Set (FAS): All randomized participants with at least 1 documented study vaccine administration, regardless of the occurrence of protocol deviations and vaccine type (seasonal influenza, Ad26.COV2.S, or placebo). Analyses of safety and participant information will be performed on the FAS.

Per Protocol Influenza Immunogenicity (PPII) Set: All randomized participants who received Ad26.COV2.S vaccine in combination with a seasonal influenza vaccine for the co-administration group and those who received a seasonal influenza vaccine alone for the control group, for whom immunogenicity data are available for at least one of the influenza strains in the vaccine.

Samples taken after a participant experiences a major protocol deviation expected to impact the immunogenicity outcomes will be excluded from the PPII analysis.

Per Protocol SARS-CoV-2 Immunogenicity (PPSI) Set: All randomized participants who received Ad26.COV2.S vaccine in combination with seasonal influenza vaccine for the co-administration group and Ad26.COV2.S vaccine alone for the control group and for whom SARS-CoV-2 immunogenicity data are available. In addition, samples obtained at later timepoints from participants after molecularly confirmed (viral RNA test result) natural SARS-CoV-2 infection will be excluded from the analysis.

Samples taken after a participant meets the criteria for a major protocol deviation expected to impact the immunogenicity outcomes will be excluded from the PPSI analysis.

The list of major protocol deviations with impact in the immunogenicity analysis will be specified in the SAP or major protocol violation criteria document. Both documents will be finalized before database lock and unblinding.

The primary analysis set for analyses related to influenza immunogenicity is the PPII Set, the primary analysis set for analyses related to SARS-CoV-2 immunogenicity is the PPSI Set. As a sensitivity analysis, key tables may also be based on the FAS.

Note: Participants who have a self-confirmed history of SARS-CoV-2 infection at screening or positive N-serology at baseline will be included in the analysis, but samples obtained after SARS-CoV-2 (or influenza) infection during the study will be excluded.

9.4. Statistical Analyses

The SAP will be finalized prior to primary analysis database lock and it will include a more technical and detailed description of the statistical analyses described in this section. This section is a summary of the planned statistical analyses of the primary and key secondary endpoints.

9.4.1. General Considerations

The primary analysis will be performed when all participants have completed the 28 days after the second study vaccination visit or discontinued earlier. No interim analyses will be performed.

9.4.2. Inferential Endpoints

The NI immunogenicity objectives will be assessed by calculating:

- the 2-sided 95% CIs for the difference in log-transformed HI antibody titers against each of the 4 influenza vaccine strains at 28 days after the administration of the seasonal influenza vaccine between the Control and the CoAd group for the *standard-dose*.
- the 2-sided 95% CIs for the difference in log-transformed S-ELISA titers at 28 days after the administration of Ad26.COV2.S vaccine between the Control and the CoAd group for the *standard-dose*.

For HI antibody titers against each of the 4 influenza vaccine strains and for S-ELISA titers, an analysis of variance (ANOVA) model will be fitted with the respective titers as dependent variable and group (Control or CoAd), age category (as stratified), previous SARS-CoV-2 vaccination (as stratified), and baseline S-ELISA titers as independent variable. Based on these ANOVA models, the CIs around the difference will be calculated and will be back transformed (by exponentiation) to CIs around a GMC or GMT ratio (Control group/CoAd group), respectively, and compared to the NI margin of 1.5.

Only if the upper bound of the 2-sided 95% CI for the GMT or GMC ratio (Control group/CoAd group), respectively, of the HI antibody titer for each of the 4 vaccine strains and for S-ELISA lies below 1.5, NI of co-administration versus separate administration will be concluded for both vaccines (seasonal influenza vaccine and Ad26.COV2.S vaccine). If 1 or more confidence limits for the GMT or GMC ratio exceed 1.5, non-inferiority cannot be concluded.

For immunogenicity, baseline is considered as the last assessment pre-vaccination (ie, it corresponds to Day 1 for CoAd groups or influenza vaccine alone, or to Day 29 for Ad26.COV2.S vaccine). In a sensitivity analysis, different variances between the groups will be allowed. Therefore, the CIs will be calculated via Welch’s ANOVA.

The assessment of the primary endpoints will be performed on individuals who have been previously vaccinated with different types of COVID-19 vaccines. This factor brings a level of variability of immune responses that is unknown.

9.4.3. Other Secondary Endpoints

For either the standard-dose or high-dose, seroconversion is defined for each of the 4 influenza vaccine strains at 28 days after the administration of a seasonal influenza vaccine:

- HI titer ≥1:40 in participants with a pre-vaccination HI titer of <1:10, or
- a ≥4-fold HI titer increase in participants with a pre-vaccination HI titer of ≥1:10

Seroprotection is defined for each of the 4 influenza vaccine strains as HI titer ≥1:40 at 28 days after the administration of a seasonal influenza vaccine.

The difference in proportions of seroconverted and seroprotected participants between the Control and the CoAd group will be estimated together with the 2-sided 95% CIs (calculated using Wilson's score method).

Serostatus at screening will be assigned according to medical records or participants' verbal reporting. For the analysis, serostatus will be based on binding antibodies. If a participant has detectable S-ELISA and/or N-ELISA above the limit of quantification, then the individual is considered seropositive. The confirmation of serostatus will be performed with a sensitive S-ELISA and N-ELISA assay. Seropositive participants are defined as previously vaccinated individuals (ie, Vaxzevria [AstraZeneca], Comirnaty [Pfizer-BioNTech], SpikeVax [Moderna], and Ad26.COV2-S [Janssen]), regardless of previous SARS-CoV2 infection. The following will be presented descriptively for the secondary analyses:

- Humoral immune response as measured by the HI assay after concomitant administration of Ad26.COV2.S vaccine and a seasonal quadrivalent *high-dose* influenza vaccine versus the administration of a seasonal quadrivalent *high-dose* influenza vaccine alone, 28 days after the administration of a seasonal influenza vaccine.
- Humoral immune response (ELISA) following concomitant administration of Ad26.COV2.S vaccine and a seasonal quadrivalent *high-dose* influenza vaccine versus the administration of Ad26.COV2.S vaccine alone, 28 days after the administration of Ad26.COV2.S vaccine.
- Humoral immune response as measured by the HI assay after concomitant administration of Ad26.COV2.S vaccine and a seasonal quadrivalent *high-dose* influenza vaccine versus the administration of a seasonal quadrivalent *high-dose* influenza vaccine alone, 28 days after the administration of a seasonal influenza vaccine *in COVID-19 vaccine-naïve individuals.*
- Humoral immune response (ELISA) following concomitant administration of Ad26.COV2.S vaccine and a seasonal quadrivalent *high-dose* influenza vaccine versus the administration of Ad26.COV2.S vaccine alone, 28 days after the administration of Ad26.COV2.S vaccine *in COVID-19 vaccine-naïve individuals.*
- Humoral immune response as measured by the HI assay after concomitant administration of Ad26.COV2.S vaccine and a seasonal quadrivalent *standard-dose* influenza vaccine versus the administration of a seasonal quadrivalent *standard-dose* influenza vaccine alone, 28 days after the administration of a seasonal influenza vaccine *in COVID-19 vaccine-naïve individuals.*

- Humoral immune response (ELISA) following concomitant administration of Ad26.COV2.S vaccine and a seasonal quadrivalent *standard-dose* influenza vaccine versus the administration of Ad26.COV2.S vaccine alone, 28 days after the administration of Ad26.COV2.S vaccine *in COVID-19 vaccine-naïve individuals.*

Additionally, HI antibody titers and S-ELISA titers will be summarized with descriptive statistics. Descriptive statistics (geometric mean and 95% CI) of the actual values will be calculated for continuous immunogenicity parameters at all timepoints. Geometric mean fold increases from baseline and corresponding 95% CIs might additionally be calculated. Graphical representations of immunogenicity parameters will be created as applicable.

Additional characterization of these participants will be included in the SAP.For categorical variables, frequency tables will be presented. Difference in proportions and corresponding CIs may be calculated where appropriate.

9.4.4. Exploratory Endpoints

Exploratory endpoint analyses will be detailed in the SAP.

9.4.5. Safety Analyses

No formal statistical testing of safety data is planned. All safety data will be analyzed descriptively by group. All safety analyses will be based on the FAS.

Adverse Events

The verbatim terms used in the eCRF by investigators to identify AEs will be coded using the Medical Dictionary for Regulatory Activities (MedDRA). All reported AEs with onset during the active vaccination phase (ie, AEs occurring after vaccination up to 28 days post-vaccination), and all SAEs/MAAEs/AESIs will be included in the analysis. (S)AEs caused by molecularly confirmed SARS-CoV-2 infection will be removed at the analysis level from the (S)AE listings and tables and presented separately. For each AE, the percentage of participants who experience at least 1 occurrence of the given event will be summarized by vaccine group.

Summaries, listings, datasets, or participant narratives may be provided, as appropriate, for those participants who die, who discontinue study vaccine due to an AE, or who experience a severe AE, an AESI, or an SAE.

Solicited local (at injection site) and systemic AEs will be summarized descriptively. The number and percentages of participants with at least one solicited local (at injection site) or systemic AE will be presented. The frequencies by vaccine group as well as frequencies according to severity and duration will be described for solicited AEs. Frequencies of unsolicited AEs, separately for all and vaccination-related only, will be presented by System Organ Class and preferred term, while those of solicited AEs will be presented only by preferred term.

Clinical Laboratory Tests

Laboratory data (abnormal or graded, when available) will be listed and/or tabulated by participant and time point.

Vital Signs

Vital signs including temperature, pulse/heart rate, respiratory rate, and blood pressure (systolic and diastolic) will be summarized over time, using descriptive statistics and/or graphically. The percentage of participants with values beyond clinically important limits will be summarized.

Physical Examinations

Physical examination findings will be summarized at baseline. Physical examination abnormal findings will be listed.

9.4.6. Other Analyses

The primary analysis of safety and immunogenicity will be performed when all participants have completed the 28 days after the second study vaccination visit or discontinued earlier. The primary analysis will be performed based on sponsor's unblinded data (sponsor, study site personnel and participants will remain blinded during the treatment phase up to the database lock). No interim analyses will be performed.

A final analysis will be performed when all participants have completed the 6 month follow-up period after the second vaccination or discontinued earlier.

9.5. Interim Analysis

Not applicable.

10. SUPPORTING DOCUMENTATION AND OPERATIONAL CONSIDERATIONS

10.1. Appendix 1: Abbreviations and Definitions

ACIP	Advisory Committee on Immunization Practices
Ad26	adenovirus type 26
ADCC	antibody-dependent cell-mediated cytotoxicity
AdVAC®	adenoviral vaccine (vector platform)
AE	adverse event
AESI	adverse event of special interest
ANOVA	analysis of variance
aPTT	activated partial thromboplastin time
AR	adverse reaction
ART	anti-retroviral treatment
BMI	body mass index
CD	cluster of differentiation
CDC	Centers for Disease Control and Prevention
CI	confidence interval
CoAd	co-administration
COVID-19	coronavirus disease-2019
CTLA-4	cytotoxic T-lymphocyte-associated antigen 4
CVST	cerebral venous sinus thrombosis
DVT	deep vein thrombosis
eCRF	electronic case report form
eDC	electronic data capture
EEA	European Economic Area
ELISA	enzyme-linked immunosorbent assay
EMA	European Medicines Agency
EU	European Union
EUA	Emergency Use Authorization
FAS	full analysis set
Fc	crystallizable fragment
FDA	Food and Drug Administration
FOIA	Freedom of Information Act
GCP	Good Clinical Practice
GMC	geometric mean concentration
GMT	geometric mean titer
HA	hemagglutinin
HAI	hemagglutinin inhibition assay
HCP	health care professional
HI	hemagglutination inhibition
HIT	heparin-induced thrombocytopenia
HITT	heparin-induced thrombocytopenia with thrombosis
HIV	human immunodeficiency virus
HPV	human papillomavirus
IB	investigator's brochure
ICF	informed consent form
ICH	International Conference on Harmonisation
ICMJE	International Committee of Medical Journal Editors
ICU	intensive care unit
IEC	Independent Ethics Committee
IFN-γ	interferon gamma
Ig	immunoglobulin
IM	intramuscular
IND	Investigational New Drug
IPPI	Investigational Product Preparation Instructions
IRB	Institutional Review Board
IWRS	interactive web response system

MAAE	Medically-attended Adverse Events
MABS	Monoclonal Antibodies
MedDRA	Medical Dictionary for Regulatory Activities
NHP	non-human primate
NI	non-inferiority
PCR	polymerase chain reaction
PCC	protocol clarification communication
PD-1	programmed cell death protein 1
PE	pulmonary embolism
PF-4	platelet factor 4
PI	principal investigator
PPI	per protocol immunogenicity
PPII	per protocol influenza immunogenicity
PPSI	per protocol SARS-CoV-2 immunogenicity
PQC	product quality complaint
PT	prothombin time
RNA	ribonucleic acid
RSV	respiratory syncytial virus
RT-PCR	real-time reverse-transcriptase polymerase chain reaction
S	Spike
SAE	serious adverse event
SAP	statistical analysis plan
SARS-CoV(-2)	severe acute respiratory syndrome coronavirus(-2)
SIPPM	site investigational product and procedures manual
SUSAR	suspected unexpected serious adverse reaction
Th	T helper
TNF-α	tumor necrosis factor alpha
TTS	thrombosis with thrombocytopenia syndrome
US	United States
VAED	vaccine-associated enhanced disease
VAERD	vaccine-associated enhanced respiratory disease
VAERS	Vaccine Adverse Event Reporting System
VNA	virus neutralization assay
vp	virus particle
VTE	venous thromboembolism
WBC	white blood cell
WHO	World Health Organization

Definitions of Terms

COVID-19	COVID-19 is the disease caused by the virus SARS-CoV-2. COVID-19 refers to SARS-CoV-2 infection with symptoms, and can range from mild to severe disease, the latter including pneumonia, severe acute respiratory syndrome, multi-organ failure, and death (FDA 2020, FDA 2021a).
e-Diary	The electronic technology used to record solicited signs and symptoms by the participants.
Study Name Convention	In this document, studies are referred to using the short study name (preceding letters and final digits of the study identifier) only (eg, COV2007).
Electronic source system	Contains data traditionally maintained in a hospital or clinic record to document medical care or data recorded in a CRF as determined by the protocol. Data in this system may be considered source documentation.

10.2. Appendix 2: Clinical Laboratory Tests

The following tests will be performed according to the Schedule of Activities:

Protocol-Required Safety Laboratory Assessments

Laboratory Assessments	Parameters			Timepoints
Testing done locally or at a substitute for local laboratory	Platelet count<br>Hemoglobin	Prothrombin time<br>Activated partial thromboplastin time	White Blood Cell (WBC) count with Differential:<br>Neutrophils<br>Lymphocytes<br>Monocytes<br>Eosinophils<br>Basophils	Pre-vaccination with the vaccine at baseline for safety monitoring and at the time of a (suspected) AESI.
Testing done centrally	Serum/plasma samples for coagulation-related assays such as but not limited to:<br>• AntiPF4<br>• Activated partial thromboplastin time<br>• Prothrombin time<br>• International normalized ratio<br>• Fibrinogen<br>• D-dimer·<br>• Lupus anticoagulant·<br>• Anti-cardiolipin antibody<br>• Beta-2 glycoprotein·<br>• Heparin-induced thrombocytopenia (HIT)/PF4 Ab, IgG·(HIT assay)<br>• Platelet activation assay (if HIT/PF4 is positive)<br>• Homocysteine·<br>• ADAMTS13 Activity and Inhibitor Profile			As part of a (suspected) AESI investigation (eg, whether thrombocytopenia is observed with a thrombotic event), all or some of these tests may be conducted on the stored pre-vaccination sample (retrospective test) and on the samples obtained at the time of the (suspected) AESI investigation.

10.3. Appendix 3: Regulatory, Ethical, and Study Oversight Considerations

10.3.1. Regulatory and Ethical Considerations

Investigator Responsibilities

The investigator is responsible for ensuring that the study is performed in accordance with the protocol, current International Council for Harmonisation (ICH) guidelines on Good Clinical Practice (GCP), and applicable regulatory and country-specific requirements.

Good Clinical Practice is an international ethical and scientific quality standard for designing, conducting, recording, and reporting studies that involve the participation of human participants. Compliance with this standard provides public assurance that the rights, safety, and well-being of study participants are protected, consistent with the principles that originated in the Declaration of Helsinki, and that the study data are credible.

Protocol Clarification Communications

If text within a final approved protocol requires clarification (eg, current wording is unclear or ambiguous) that does not change any aspect of the current study conduct, a protocol clarification communication (PCC) may be prepared. The PCC Document will be communicated to the Investigational Site, Site Monitors, Local Trial Managers (LTMs), Clinical Trial Managers (CTMs), and/or Contract Research Organizations (CROs) who will ensure that the PCC explanations are followed by the investigators.

The PCC Document may be shared by the sites with IECs/IRBs per local regulations.

The PCC Documents must NOT be used in place of protocol amendments, but the content of the PCC Document must be included in any future protocol amendments.

Protocol Amendments

Neither the investigator nor the sponsor will modify this protocol without a formal amendment by the sponsor. All protocol amendments must be issued by the sponsor, and signed and dated by the investigator. Protocol amendments must not be implemented without prior IEC/IRB approval, or when the relevant competent authority has raised any grounds for non-acceptance, except when necessary to eliminate immediate hazards to the participants, in which case the amendment must be promptly submitted to the IEC/IRB and relevant competent authority. Documentation of amendment approval by the investigator and IEC/IRB must be provided to the sponsor. When the change(s) involve only logistic or administrative aspects of the study, the IEC/IRB (where required) only needs to be notified.

In situations where a departure from the protocol is unavoidable during the study, the investigator or other physician in attendance will contact the appropriate sponsor representative listed in the Contact Information page(s), which will be provided as a separate document. Except in emergency situations, this contact should be made before implementing any departure from the protocol. In all cases, contact with the sponsor must be made as soon as possible to discuss the situation and agree on an appropriate course of action. The data recorded in the eCRF and source documents

will reflect any departure from the protocol, and the source documents will describe this departure and the circumstances requiring it.

Regulatory Approval/Notification

This protocol and any amendment(s) must be submitted to the appropriate regulatory authorities in each respective country, if applicable. A study may not be initiated until all local regulatory requirements are met.

Required Prestudy Documentation

The following documents must be provided to the sponsor before shipment of study vaccine to the study site:

- Protocol and amendment(s), if any, signed and dated by the principal investigator
- A copy of the dated and signed (or sealed, where appropriate per local regulations), written IEC/IRB approval of the protocol, amendments, ICF, any recruiting materials, and if applicable, participant compensation programs. This approval must clearly identify the specific protocol by title and number and must be signed (or sealed, where appropriate per local regulations) by the chairman or authorized designee.
- Name and address of the IEC/IRB, including a current list of the IEC/IRB members and their function, with a statement that it is organized and operates according to GCP and the applicable laws and regulations. If accompanied by a letter of explanation, or equivalent, from the IEC/IRB, a general statement may be substituted for this list. If an investigator or a member of the study site personnel is a member of the IEC/IRB, documentation must be obtained to state that this person did not participate in the deliberations or in the vote/opinion of the study.
- Regulatory authority approval or notification, if applicable
- Signed and dated statement of investigator (eg, Form FDA 1572), if applicable
- Documentation of investigator qualifications (eg, curriculum vitae)
- Completed investigator financial disclosure form from the principal investigator, where required
- Signed and dated Clinical Trial Agreement, which includes the financial agreement
- Any other documentation required by local regulations

The following documents must be provided to the sponsor before enrollment of the first participant:

- Completed investigator financial disclosure forms from all subinvestigators
- Documentation of subinvestigator qualifications (eg, curriculum vitae)
- Name and address of any local laboratory conducting tests for the study, and a dated copy of current laboratory normal ranges for these tests, if applicable
- Local laboratory documentation demonstrating competence and test reliability (eg, accreditation/license), if applicable

Independent Ethics Committee or Institutional Review Board

Before the start of the study, the investigator (or sponsor where required) will provide the IEC/IRB with current and complete copies of the following documents (as required by local regulations):

- Final protocol and, if applicable, amendments
- Sponsor-approved ICF (and any other written materials to be provided to the participants)
- IB (or equivalent information) and amendments/addenda
- Sponsor-approved participant recruiting materials
- Information on compensation for study-related injuries or payment to participants for participation in the study, if applicable
- Investigator's curriculum vitae or equivalent information (unless not required, as documented by the IEC/IRB)
- Information regarding funding, name of the sponsor, institutional affiliations, other potential conflicts of interest, and incentives for participants
- Any other documents that the IEC/IRB requests to fulfill its obligation

This study will be undertaken only after the IEC/IRB has given full approval of the final protocol, amendments (if any, excluding the ones that are purely administrative, with no consequences for participants, data or study conduct, unless required locally), the ICF, applicable recruiting materials, and participant compensation programs, and the sponsor has received a copy of this approval. This approval letter must be dated and must clearly identify the IEC/IRB and the documents being approved.

During the study the investigator (or sponsor where required) will send the following documents and updates to the IEC/IRB for their review and approval, where appropriate:

- Protocol amendments (excluding the ones that are purely administrative, with no consequences for participants, data or study conduct)
- Revision(s) to ICF and any other written materials to be provided to participants
- If applicable, new or revised participant recruiting materials approved by the sponsor
- Revisions to compensation for study-related injuries or payment to participants for participation in the study, if applicable
- New edition(s) of the IB and amendments/addenda
- Summaries of the status of the study at intervals stipulated in guidelines of the IEC/IRB (at least annually)
- Reports of AEs that are serious, unlisted/unexpected, and associated with the study vaccine
- New information that may adversely affect the safety of the participants or the conduct of the study
- Deviations from or changes to the protocol to eliminate immediate hazards to the participants

- Report of deaths of participants under the investigator's care
- Notification if a new investigator is responsible for the study at the site
- Development Safety Update Report and Line Listings, where applicable
- Any other requirements of the IEC/IRB

For all protocol amendments (excluding the ones that are purely administrative, with no consequences for participants, data or study conduct), the amendment and applicable ICF revisions must be submitted promptly to the IEC/IRB for review and approval before implementation of the change(s).

At least once a year, the IEC/IRB will be asked to review and reapprove this study, where required.

At the end of the study, the investigator (or sponsor where required) will notify the IEC/IRB about the study completion.

Country Selection

This study will only be conducted in those countries where the intent is to launch or otherwise help ensure access to the developed product if the need for the product persists, unless explicitly addressed as a specific ethical consideration in Section 4.2.1.

Other Ethical Considerations

For study-specific ethical design considerations, refer to Section 4.2.1.

10.3.2. Financial Disclosure

Investigators and sub-investigators will provide the sponsor with sufficient, accurate financial information in accordance with local regulations to allow the sponsor to submit complete and accurate financial certification or disclosure statements to the appropriate regulatory authorities. Investigators are responsible for providing information on financial interests during the study and for 1 year after completion of the study.

Refer to Required Prestudy Documentation (above) for details on financial disclosure.

10.3.3. Informed Consent Process

Each participant must give written consent according to local requirements after the nature of the study has been fully explained. The ICF(s) must be signed before performance of any study-related activity. The ICF(s) that is/are used must be approved by both the sponsor and by the reviewing IEC/IRB and be in a language that the participant can read and understand. The informed consent should be in accordance with principles that originated in the Declaration of Helsinki, current ICH and GCP guidelines, applicable regulatory requirements, and sponsor policy.

Before enrollment in the study, the investigator or an authorized member of the study site personnel must explain to potential participants the aims, methods, reasonably anticipated benefits, and potential hazards of the study, and any discomfort participation in the study may entail. Participants will be informed that their participation is voluntary and that they may withdraw

consent to participate at any time. They will be informed that choosing not to participate will not affect the care the participant will receive. Finally, they will be told that the investigator will maintain a participant identification register for the purposes of long-term follow-up if needed and that their records may be accessed by health authorities and authorized sponsor personnel without violating the confidentiality of the participant, to the extent permitted by the applicable law(s) or regulations. By signing the ICF the participant is authorizing such access. It also denotes that the participant agrees to allow his or her study physician to recontact the participant for the purpose of obtaining consent for additional safety evaluations, if needed.

The participant will be given sufficient time to read the ICF and the opportunity to ask questions. After this explanation and before entry into the study, consent should be appropriately recorded by means of the participant's personally dated signature. After having obtained the consent, a copy of the ICF must be given to the participant.

Participants who are rescreened are required to sign a new ICF.

If the participant is unable to read or write, an impartial witness should be present for the entire informed consent process (which includes reading and explaining all written information) and should personally date and sign the ICF after the oral consent of the participant is obtained.

10.3.4. Data Protection

Privacy of Personal Data

The collection and processing of personal data from participants enrolled in this study will be limited to those data that are necessary to fulfill the objectives of the study.

These data must be collected and processed with adequate precautions to ensure confidentiality and compliance with applicable data privacy protection laws and regulations. Appropriate technical and organizational measures to protect the personal data against unauthorized disclosures or access, accidental or unlawful destruction, or accidental loss or alteration must be put in place. Sponsor personnel whose responsibilities require access to personal data agree to keep the identity of participants confidential.

The informed consent obtained from the participant includes explicit consent for the processing of personal data and for the investigator/institution to allow direct access to his or her original medical records (source data/documents) for study-related monitoring, audit, IEC/IRB review, and regulatory inspection. This consent also addresses the transfer of the data to other entities and to other countries.

The participant has the right to request through the investigator access to his or her personal data and the right to request rectification of any data that are not correct or complete. Reasonable steps will be taken to respond to such a request, taking into consideration the nature of the request, the conditions of the study, and the applicable laws and regulations.

Exploratory research is not conducted under standards appropriate for the return of data to participants. In addition, the sponsor cannot make decisions as to the significance of any findings resulting from exploratory research. Therefore, exploratory research data will not be returned to participants or investigators, unless required by law or local regulations. Privacy and confidentiality of data generated in the future on stored samples will be protected by the same standards applicable to all other clinical data.

10.3.5. Long-Term Retention of Samples for Additional Future Research

Samples collected in this study may be stored for up to 15 years (or according to local regulations) for additional research. Samples will only be used to understand Ad26.COV2.S, to understand SARS-CoV2.S infection, to understand differential intervention responders, and to develop tests/assays related to Ad26-based vaccines, including Ad26.COV2.S and SARS-CoV2.S infection. The research may begin at any time during the study or the post study storage period.

Stored samples will be coded throughout the sample storage and analysis process and will not be labeled with personal identifiers. Participants may withdraw their consent for their samples to be stored for research (refer to Section 7.2.1).

10.3.6. Committees Structure

AESI Adjudication Committee

An AESI Adjudication Committee with appropriate expertise will be established to evaluate each suspected AESI and determine whether it is a case of TTS (see Section 8.3.6). A Charter will be developed to describe the roles and responsibilities of the Committee.

10.3.7. Publication Policy/Dissemination of Clinical Study Data

All information, including but not limited to information regarding Ad26.COV2.S or the sponsor's operations (eg, patent application, formulas, manufacturing processes, basic scientific data, prior clinical data, formulation information) supplied by the sponsor to the investigator and not previously published, and any data, including exploratory research data, generated as a result of this study, are considered confidential and remain the sole property of the sponsor. The investigator agrees to maintain this information in confidence and use this information only to accomplish this study and will not use it for other purposes without the sponsor's prior written consent.

The investigator understands that the information developed in the study will be used by the sponsor in connection with the continued development of Ad26.COV2.S, and thus may be disclosed as required to other clinical investigators or regulatory agencies. To permit the information derived from the clinical studies to be used, the investigator is obligated to provide the sponsor with all data obtained in the study.

The results of the study will be reported in a Clinical Study Report generated by the sponsor and will contain data from all study sites that participated in the study as per protocol. Recruitment performance or specific expertise related to the nature and the key assessment parameters of the study will be used to determine a coordinating investigator for the study. Results of exploratory

analyses performed after the Clinical Study Report has been issued will be reported in a separate report and will not require a revision of the Clinical Study Report.

Study participant identifiers will not be used in publication of results. Any work created in connection with performance of the study and contained in the data that can benefit from copyright protection (except any publication by the investigator as provided for below) shall be the property of the sponsor as author and owner of copyright in such work.

Consistent with Good Publication Practices and International Committee of Medical Journal Editors (ICMJE) guidelines, the sponsor shall have the right to publish such primary (multicenter) data and information without approval from the investigator. The investigator has the right to publish study site-specific data after the primary data are published. If an investigator wishes to publish information from the study, a copy of the manuscript must be provided to the sponsor for review at least 60 days before submission for publication or presentation. Expedited reviews will be arranged for abstracts, poster presentations, or other materials. If requested by the sponsor in writing, the investigator will withhold such publication for up to an additional 60 days to allow for filing of a patent application. In the event that issues arise regarding scientific integrity or regulatory compliance, the sponsor will review these issues with the investigator. The sponsor will not mandate modifications to scientific content and does not have the right to suppress information. For multicenter study designs and sub-study approaches, secondary results generally should not be published before the primary endpoints of a study have been published. Similarly, investigators will recognize the integrity of a multicenter study by not submitting for publication data derived from the individual study site until the combined results from the completed study have been submitted for publication, within 18 months after the study end date, or the sponsor confirms there will be no multicenter study publication. Authorship of publications resulting from this study will be based on the guidelines on authorship, such as those described in the ICMJE Recommendations for the Conduct, Reporting, Editing and Publication of Scholarly Work in Medical Journals, which state that the named authors must have made a significant contribution to the conception or design of the work; or the acquisition, analysis, or interpretation of the data for the work; and drafted the work or revised it critically for important intellectual content; and given final approval of the version to be published; and agreed to be accountable for all aspects of the work in ensuring that questions related to the accuracy or integrity of any part of the work are appropriately investigated and resolved.

Registration of Clinical Studies and Disclosure of Results

The sponsor will register and disclose the existence of and the results of clinical studies as required by law. The disclosure of the final study results will be performed after the end of study in order to ensure the statistical analyses are relevant.

10.3.8. Data Quality Assurance

Data Quality Assurance/Quality Control

Steps to be taken to ensure the accuracy and reliability of data include the selection of qualified investigators and appropriate study sites, review of protocol procedures with the investigator and

study site personnel before the study, and periodic monitoring visits by the sponsor. Written instructions will be provided for collection, handling, storage, and shipment of samples.

Guidelines for eCRF completion will be provided and reviewed with study site personnel before the start of the study. The sponsor may review the eCRF for accuracy and completeness during on-site monitoring visits and after transmission to the sponsor; any discrepancies will be resolved with the investigator or designee, as appropriate. After upload of the data into the study database they will be verified for accuracy and consistency with the data sources.

10.3.9. Case Report Form Completion

Case report forms are prepared and provided by the sponsor for each participant in electronic format. All data relating to the study must be recorded in the eCRF. All eCRF entries, corrections, and alterations must be made by the investigator or authorized study site personnel. The investigator must verify that all data entries in the eCRF are accurate and correct.

The study data will be transcribed by study site personnel from the source documents onto an eCRF, if applicable. Study-specific data will be transmitted in a secure manner to the sponsor.

Worksheets may be used for the capture of some data to facilitate completion of the eCRF. Any such worksheets will become part of the participant's source documents. Data must be entered into the eCRF in English. The eCRF must be completed as soon as possible after a participant visit and the forms should be available for review at the next scheduled monitoring visit.

All participative measurements (eg, questionnaires) will be completed by the same individual who made the initial baseline determinations whenever possible.

If necessary, queries will be generated in the electronic data capture (eDC) tool. If corrections to an eCRF are needed after the initial entry into the eCRF, this can be done in either of the following ways:

- Investigator and study site personnel can make corrections in the eDC tool at their own initiative or as a response to an auto query (generated by the eDC tool).
- Sponsor or sponsor delegate can generate a query for resolution by the investigator and study site personnel.

10.3.10. Source Documents

At a minimum, source documents consistent in the type and level of detail with that commonly recorded at the study site as a basis for standard medical care must be available for the following: participant identification, eligibility, and study identification; study discussion and date of signed informed consent; dates of visits; results of safety and efficacy parameters as required by the protocol; record of all AEs and follow-up of AEs; concomitant medication; intervention receipt/dispensing/return records; study vaccine administration information; and date of study completion and reason for early discontinuation of study vaccine or withdrawal from the study, if applicable.

The author of an entry in the source documents should be identifiable.

Participant- and investigator-completed scales and assessments designated by the sponsor (ie, diary to record solicited AEs) will be recorded and will be considered source data. The participant's diary used to collect information regarding solicited signs and symptoms after vaccination will be considered source data.

Specific details required as source data for the study and source data collection methods will be reviewed with the investigator before the study and will be described in the monitoring guidelines (or other equivalent document).

An eSource system may be utilized, which contains data traditionally maintained in a hospital or clinic record to document medical care (eg, electronic source documents) as well as the clinical study-specific data fields as determined by the protocol. These data are electronically extracted for use by the sponsor. If eSource is utilized, references made to the eCRF in the protocol include the eSource system but information collected through eSource may not be limited to that found in the eCRF.

10.3.11. Monitoring

The sponsor will use a combination of monitoring techniques (central, remote, and/or on-site monitoring) to monitor this study.

The sponsor will perform on-site monitoring visits as frequently as necessary. The monitor will record dates of the visits in a study site visit log that will be kept at the study site. The first post-initiation visit will be made as soon as possible after enrollment has begun. At these visits, the monitor may compare the data entered into the eCRF with the source documents (eg, hospital/clinic/physician's office medical records). The nature and location of all source documents will be identified to ensure that all sources of original data required to complete the eCRF are known to the sponsor and study site personnel and are accessible for verification by the sponsor study site contact. If electronic records are maintained at the study site, the method of verification must be discussed with the study site personnel.

Direct access to source documents (medical records) must be allowed for the purpose of verifying that the recorded data are consistent with the original source data. Findings from this review will be discussed with the study site personnel. The sponsor expects that, during monitoring visits, the relevant study site personnel will be available, the source documents will be accessible, and a suitable environment will be provided for review of study-related documents. The monitor will meet with the investigator on a regular basis during the study to provide feedback on the study conduct.

In addition to on-site monitoring visits, remote contacts can occur. It is expected that during these remote contacts, study site personnel will be available to provide an update on the progress of the study at the site.

Central monitoring will take place for data identified by the sponsor as requiring central review.

10.3.12. On-Site Audits

Representatives of the sponsor's clinical quality assurance department may visit the study site at any time during or after completion of the study to conduct an audit of the study in compliance with regulatory guidelines and company policy. These audits will require access to all study records, including source documents, for inspection. Participant privacy must, however, be respected. The investigator and study site personnel are responsible for being present and available for consultation during routinely scheduled study site audit visits conducted by the sponsor or its designees.

Similar auditing procedures may also be conducted by agents of any regulatory body, either as part of a national GCP compliance program or to review the results of this study in support of a regulatory submission. The investigator should immediately notify the sponsor if he or she has been contacted by a regulatory agency concerning an upcoming inspection.

10.3.13. Record Retention

In compliance with the ICH/GCP guidelines, the investigator/institution will maintain all eCRF and all source documents that support the data collected from each participant, as well as all study documents as specified in ICH/GCP Section 8, Essential Documents for the Conduct of a Clinical Trial, and all study documents as specified by the applicable regulatory requirement(s). The investigator/institution will take measures to prevent accidental or premature destruction of these documents.

Essential documents must be retained until at least 2 years after the last approval of a marketing application in an ICH region and until there are no pending or contemplated marketing applications in an ICH region or until at least 2 years have elapsed since the formal discontinuation of clinical development of the investigational product. These documents will be retained for a longer period if required by the applicable regulatory requirements or by an agreement with the sponsor. It is the responsibility of the sponsor to inform the investigator/institution as to when these documents no longer need to be retained.

If the responsible investigator retires, relocates, or for other reasons withdraws from the responsibility of keeping the study records, custody must be transferred to a person who will accept the responsibility. The sponsor must be notified in writing of the name and address of the new custodian. Under no circumstance shall the investigator relocate or dispose of any study documents before having obtained written approval from the sponsor.

If it becomes necessary for the sponsor or the appropriate regulatory authority to review any documentation relating to this study, the investigator/institution must permit access to such reports.

10.3.14. Study and Site Start and Closure

First Act of Recruitment

The first site open is considered the first act of recruitment and it becomes the study start date.

Study/Site Termination

The sponsor reserves the right to close the study site or terminate the study at any time for any reason at the sole discretion of the sponsor. Study sites will be closed upon study completion. A study site is considered closed when all required documents and study supplies have been collected and a study site closure visit has been performed.

The investigator may initiate study site closure at any time, provided there is reasonable cause and sufficient notice is given in advance of the intended termination.

Reasons for the early closure of a study site by the sponsor or investigator may include but are not limited to:

- Failure of the investigator to comply with the protocol, the requirements of the IEC/IRB or local health authorities, the sponsor's procedures, or GCP guidelines
- Inadequate recruitment of participants by the investigator
- Discontinuation of further study vaccine development

10.4. Appendix 4: Adverse Events, Serious Adverse Events, Adverse Events of Special Interest, Product Quality Complaints, and Other Safety Reporting: Definitions and Procedures for Recording, Evaluating, Follow-up, and Reporting

10.4.1. Adverse Event Definitions and Classifications

Adverse Event

An AE is any untoward medical occurrence in a clinical study participant administered a pharmaceutical (investigational or non-investigational) product. An AE does not necessarily have a causal relationship with the intervention. An AE can therefore be any unfavorable and unintended sign (including an abnormal finding), symptom, or disease temporally associated with the use of a medicinal (investigational or non-investigational) product, whether or not related to that medicinal (investigational or non-investigational) product. (Definition per ICH)

This includes any occurrence that is new in onset or aggravated in severity or frequency from the baseline condition, or abnormal results of diagnostic procedures, including laboratory test abnormalities.

Any respiratory tract infection that is not due to SARS-CoV-2 infection will be reported as an AE if it occurs between the time of any vaccination through the following 28 days. Any respiratory tract infection recorded as an AE in the eCRF will be excluded from any AE analysis if the molecular test is subsequently found to be positive for SARS-CoV-2. Respiratory tract infections arising from SARS-CoV-2 infection will not be reported as (S)AEs in the Clinical Study Report but will be tabulated separately. In general, any (S)AEs caused by molecularly confirmed SARS-CoV-2 infection will be removed at the analysis level from the (S)AE listings and tables and presented separately. In general, (S)AEs caused by molecularly confirmed SARS-CoV-2 infection will be removed at the analysis level from the (S)AE listings and tables and presented separately.

Note: For time period of sponsor's AE collection, see All Adverse Events under Section 8.3.1.

Serious Adverse Event

An SAE based on ICH and EU Guidelines on Pharmacovigilance for Medicinal Products for Human Use is any untoward medical occurrence that at any dose:

- Results in death
- Is life-threatening
  (The participant was at risk of death at the time of the event. It does not refer to an event that hypothetically might have caused death if it were more severe.)
- Requires inpatient hospitalization or prolongation of existing hospitalization
- Results in persistent or significant disability/incapacity
- Is a congenital anomaly/birth defect

- Is a suspected transmission of any infectious agent via a medicinal product
- Is Medically Important*

  *Medical and scientific judgment should be exercised in deciding whether expedited reporting is also appropriate in other situations, such as important medical events that may not be immediately life-threatening or result in death or hospitalization but may jeopardize the participant or may require intervention to prevent one of the other outcomes listed in the definition above. These should usually be considered serious.

If a serious and unexpected AE occurs for which there is evidence suggesting a causal relationship between the study vaccine and the event (eg, death from anaphylaxis), the event must be reported as a serious and unexpected suspected adverse reaction even if it is a component of the study endpoint (eg, all-cause mortality).

Unlisted (Unexpected) Adverse Event/Reference Safety Information

An AE is considered unlisted if the nature or severity is not consistent with the applicable product reference safety information. For Ad26.COV2.S, the expectedness of an AE will be determined by whether or not it is listed in the IB.

10.4.2. Attribution Definitions

Assessment of Causality

The causal relationship to study vaccine is determined by the investigator. The following selection should be used to assess all AEs.

Related

There is a reasonable causal relationship between study vaccine administration and the AE.

Not Related

There is not a reasonable causal relationship between study vaccine administration and the AE.

The term "reasonable causal relationship" means there is evidence to support a causal relationship.

By definition, all solicited adverse events at the injection site (local) will be considered related to the study vaccine administration.

10.4.3. Severity Criteria

All AEs and laboratory data will be coded for severity using a modified version of the FDA grading table, based on version of September 2007 (US DHHS 2007), included in Section 10.6, Appendix 6.

For AEs not identified in the grading table, the following guidelines will be applied:

**Grade 1**	Mild	Symptoms causing no or minimal interference with usual social and functional activities
**Grade 2**	Moderate	Symptoms causing greater than minimal interference with usual social and functional activities
**Grade 3**	Severe	Symptoms causing inability to perform usual social and functional activities and requires medical intervention
**Grade 4**	Potentially life-threatening	Symptoms causing inability to perform basic self-care functions OR medical or operative intervention indicated to prevent permanent impairment, persistent disability OR emergency room visit or hospitalization

The severity of solicited signs and symptoms will be graded in the e-Diary by the participant based on the severity assessment provided in the e-Diary and then verified by the investigator using the toxicity grading scale in Section 10.6, Appendix 6. (Note: severity of the measured events will be derived from the diameter [for erythema and swelling] and the temperature measurements [for fever]).

10.4.4. Special Reporting Situations

Safety events of interest on a sponsor study vaccine in an interventional study that may require expedited reporting or safety evaluation include, but are not limited to:

- Overdose of a sponsor study vaccine
- Suspected abuse/misuse of a sponsor study vaccine
- Accidental or occupational exposure to a sponsor study vaccine
- Medication error, intercepted medication error, or potential medication error involving a Johnson & Johnson medicinal product (with or without patient exposure to the Johnson & Johnson medicinal product, eg, product name confusion, product label confusion, intercepted prescribing or dispensing errors)
- Exposure to a sponsor study vaccine from breastfeeding

Special reporting situations should be recorded in the eCRF. Any special reporting situation that meets the criteria of an SAE should be recorded on the SAE page of the CRF.

10.4.5. Procedures

All Adverse Events

All AEs, regardless of seriousness, severity, or presumed relationship to study vaccine, must be recorded using medical terminology in the source document and the eCRF. Whenever possible, diagnoses should be given when signs and symptoms are due to a common etiology (eg, cough, runny nose, sneezing, sore throat, and head congestion should be reported as "upper respiratory

infection"). Investigators must record in the eCRF their opinion concerning the relationship of the AE to study therapy. All measures required for AE management must be recorded in the source document and reported according to sponsor instructions.

For all studies with an outpatient phase, including open-label studies, the participant must be provided with a "wallet (study) card" and instructed to carry this card with them for the duration of the study indicating the following:

- Study number
- Statement, in the local language(s), that the participant is participating in a clinical study
- Investigator's name and 24hour contact telephone number
- Local sponsor's name and 24-hour contact telephone number (for medical personnel only)
- Site number
- Participant number
- Any other information that is required to do an emergency breaking of the blind

Serious Adverse Events

All SAEs that have not resolved by the end of the study, or that have not resolved upon the participant's discontinuation from the study, must be followed until any of the following occurs:

- The event resolves
- The event stabilizes
- The event returns to baseline, if a baseline value/status is available
- The event can be attributed to agents other than the study vaccine or to factors unrelated to study conduct
- It becomes unlikely that any additional information can be obtained (participant or health care practitioner refusal to provide additional information, lost to follow-up after demonstration of due diligence with follow-up efforts)

Any event requiring hospitalization (or prolongation of hospitalization) that occurs during participation in the study must be reported as an SAE, except hospitalizations for the following:

- Hospitalizations not intended to treat an acute illness or AE (eg, social reasons such as pending placement in long-term care facility)
- Surgery or procedure planned before entry into the study (must be documented in the eCRF). Note: Hospitalizations that were planned before the signing of the ICF, and where the underlying condition for which the hospitalization was planned has not worsened, will not be considered SAEs. Any AE that results in a prolongation of the originally planned hospitalization is to be reported as a new SAE.

The cause of death of a participant in a study, whether or not the event is expected or associated with the study vaccine, is considered an SAE.

Information regarding SAEs will be transmitted to the sponsor using an SAE reporting form and safety report form of the eCRF, which must be completed and reviewed by a physician from the study site, and transmitted in a secure manner to the sponsor within 24 hours. The initial and follow-up reports of an SAE should be transmitted in a secure manner electronically or by facsimile (fax). Telephone reporting should be the exception and the reporter should be asked to complete the appropriate form(s) first.

Adverse Events of Special Interest

AESIs will be carefully monitored during the study by the sponsor. Suspected AESIs must be reported to the sponsor within 24 hours of awareness irrespective of seriousness (ie, serious and non-serious AEs) or causality assessment, following the procedure described above for SAEs and will require enhanced data collection.

10.4.6. Product Quality Complaint Handling

Definition

A PQC is defined as any suspicion of a product defect related to manufacturing, labeling, or packaging, ie, any dissatisfaction relative to the identity, quality, durability, reliability, or performance of a distributed product, including its labeling, drug delivery system, or package integrity. A PQC may have an impact on the safety and efficacy of the product. In addition, it includes any technical complaints, defined as any complaint that indicates a potential quality issue during manufacturing, packaging, release testing, stability monitoring, dose preparation, storage or distribution of the product or the drug delivery system.

Procedures

All initial PQCs must be reported to the sponsor by the study site personnel within 24 hours after being made aware of the event.

A sample of the suspected product should be maintained under the correct storage conditions until a shipment request is received from the sponsor.

10.4.7. Contacting Sponsor Regarding Safety, Including Product Quality

The names (and corresponding telephone numbers) of the individuals who should be contacted regarding safety issues, PQC, or questions regarding the study are listed in the Contact Information page(s), which will be provided as a separate document.

10.5. Appendix 5: Contraceptive Guidance and Collection of Pregnancy Information

Participants must follow contraceptive measures as outlined in Section 5.1. Pregnancy information will be collected and reported as noted in Section 8.3.5, Pregnancy and Section 10.4.

Definition of a Person of Childbearing Potential

A Person of Childbearing Potential

A person is considered fertile following menarche and until becoming postmenopausal unless permanently sterile (see below).

A Person Not of Childbearing Potential

- **premenarchal**
  A premenarchal state is one in which menarche has not yet occurred.
- **postmenopausal**
  A postmenopausal state is defined as no menses for 12 months without an alternative medical cause.
- **permanently sterile (for the purpose of this study)**
  Permanent sterilization methods include total hysterectomy, or bilateral salpingectomy, or bilateral oophorectomy.

Note: If the childbearing potential changes after start of the study (eg, a premenarchal person experiences menarche) or the risk of pregnancy changes (eg, a person who is not heterosexually active becomes active), a person must begin an acceptable effective method of contraception, as described throughout the inclusion criteria.

10.6. Appendix 6: Toxicity Grading Scale

Adapted from the FDA Guidance document "Toxicity Grading Scale for Healthy Adult and Adolescent Volunteers Enrolled in Preventive Vaccine Clinical Trials" (September 2007) (US DHHS 2007).

A: Tables for Clinical Abnormalities

**Local Reaction to Injectable Product**	**Mild (Grade 1)**	**Moderate (Grade 2)**	**Severe (Grade 3)**	**Potentially Life Threatening (Grade 4)**
Pain/Tenderness#	Aware of symptoms but easily tolerated; Does not interfere with activity; Discomfort only to touch	Notable symptoms; Requires modification in activity or use of medications; Discomfort with movement	Incapacitating symptoms; Inability to do work, school, or usual activities; Use of narcotic pain reliever	Hospitalization; Pain/tenderness causing inability to perform basic self-care function
Erythema#	25 – 50 mm	51 – 100 mm	> 100 mm	Hospitalization; Necrosis or exfoliative dermatitis
Swelling#	25 – 50 mm	51 – 100 mm	> 100 mm	Hospitalization; Necrosis

Revised by the sponsor.

**Vital Signs ***	**Mild (Grade 1)**	**Moderate (Grade 2)**	**Severe (Grade 3)**	**Potentially Life Threatening (Grade 4)**
Fever (°C) ** (°F)**	38.0 - 38.4<br>100.4 - 101.1	38.5 - 38.9<br>101.2 - 102.0	39.0 - 40.0<br>102.1 - 104.0	> 40<br>> 104.0
Tachycardia - beats per minute	101 – 115	116 – 130	> 130	Hospitalization for arrhythmia#
Bradycardia - beats per minute***	50 – 54	45 – 49	< 45	Hospitalization for arrhythmia#
Hypertension (systolic) - mm Hg	141 – 150	151 – 155	> 155	Hospitalization for malignant hypertension#
Hypertension (diastolic) - mm Hg	91 – 95	96 – 100	> 100	Hospitalization for malignant hypertension#
Hypotension (systolic) – mm Hg	85 – 89	80 – 84	< 80	Hospitalization for hypotensive shock#
Respiratory Rate – breaths per minute	17 – 20	21 – 25	> 25	Intubation

* Participant should be at rest for all vital sign measurements.
** For oral temperature: no recent hot or cold beverages or smoking.
*** When resting heart rate is between 60 – 100 beats per minute. Use clinical judgment when characterizing bradycardia among some healthy participant populations, for example, conditioned athletes.
# Revised by the sponsor.

Systemic (General)	Mild (Grade 1)	Moderate (Grade 2)	Severe (Grade 3)	Potentially Life Threatening (Grade 4)
Vomiting#	No interference with activity or 1 – 2 episodes/24 hours	Some interference with activity or > 2 episodes/24 hours	Prevents daily activity, requires outpatient IV hydration	Hospitalization; Hypotensive shock
Nausea#	Minimal symptoms; causes minimal or no interference with work, school, or self-care activities	Notable symptoms; Requires modification in activity or use of medications; Does NOT result in loss of work, school, or cancellation of social activities	Incapacitating symptoms; Requires bed rest and/or results in loss of work, school, or cancellation of social activities	Hospitalization; Inability to perform basic self-care functions
Diarrhea#	2 – 3 loose stools or < 400 gms/24 hours	4 – 5 stools or 400 – 800 gms/24 hours	6 or more watery stools or > 800 gms/24 hours or oral rehydration necessary	Hospitalization; Hypotensive shock OR IV fluid replacement indicated
Headache#	Minimal symptoms; causes minimal or no interference with work, school, or self-care activities	Notable symptoms; Requires modification in activity or use of medications; Does NOT result in loss of work, school, or cancellation of social activities	Incapacitating symptoms; Requires bed rest and/or results in loss of work, school, or cancellation of social activities; Use of narcotic pain reliever	Hospitalization; Inability to perform basic self-care functions
Fatigue#	Minimal symptoms; causes minimal or no interference with work, school, or self-care activities	Notable symptoms; Requires modification in activity or use of medications; Does NOT result in loss of work, school, or cancellation of social activities	Incapacitating symptoms; Requires bed rest and/or results in loss of work, school, or cancellation of social activities; Use of narcotic pain reliever	Hospitalization; Inability to perform basic self-care functions
Myalgia#	Minimal symptoms; causes minimal or no interference with work, school, or self-care activities	Notable symptoms; Requires modification in activity or use of medications; Does NOT result in loss of work, school, or cancellation of social activities	Incapacitating symptoms; Requires bed rest and/or results in loss of work, school, or cancellation of social activities; Use of narcotic pain reliever	Hospitalization; Inability to perform basic self-care functions

Revised by the sponsor.

Systemic Illness	Mild (Grade 1)	Moderate (Grade 2)	Severe (Grade 3)	Potentially Life Threatening (Grade 4)
Illness or clinical adverse event (as defined according to applicable regulations)	No interference with activity	Some interference with activity not requiring medical intervention	Prevents daily activity and requires medical intervention	Hospitalization[#]

[#] Revised by the sponsor.

B: Tables for Laboratory Abnormalities

Laboratory tests may be performed during routine medical care and assessment of AEs or other medical events based on the investigator's judgment.

The laboratory values provided in the tables below serve as guidelines and are dependent upon institutional normal parameters. Institutional normal reference ranges should be provided to demonstrate that they are appropriate.

Serum *	Mild (Grade 1)	Moderate (Grade 2)	Severe (Grade 3)	Potentially Life Threatening (Grade 4)**
Sodium – Hyponatremia mEq/L	132 – 134	130 – 131	125 – 129	<125
Sodium – Hypernatremia mEq/L	144 – 145	146 – 147	148 – 150	>150
Potassium – Hyperkalemia mEq/L	5.1 – 5.2	5.3 – 5.4	5.5 – 5.6	>5.6
Potassium – Hypokalemia mEq/L	3.5 – 3.6	3.3 – 3.4	3.1 – 3.2	<3.1
Glucose – Hypoglycemia mg/dL	65 – 69	55 – 64	45 – 54	<45
Glucose – Hyperglycemia<br>Fasting mg/dL<br>Random – mg/dL	 100 – 110<br>110 – 125	 111 – 125<br>126 – 200	 >125<br>>200	Insulin requirements or hyperosmolar coma
Blood Urea Nitrogen (BUN) mg/dL	23 – 26	27 – 31	> 31	Requires dialysis
Creatinine – mg/dL	1.5 – 1.7	1.8 – 2.0	2.1 – 2.5	>2.5 or requires dialysis
Calcium – hypocalcemia mg/dL	8.0 – 8.4	7.5 – 7.9	7.0 – 7.4	<7.0
Calcium – hypercalcemia mg/dL	10.5 – 11.0	11.1 – 11.5	11.6 – 12.0	>12.0
Magnesium – hypomagnesemia mg/dL	1.3 – 1.5	1.1 – 1.2	0.9 – 1.0	<0.9
Phosphorous – hypophosphatemia mg/dL	2.3 – 2.5	2.0 – 2.2	1.6 – 1.9	<1.6
Creatine phosphokinase (CPK) – mg/dL	1.25 – 1.5 x ULN***	1.6 – 3.0 x ULN	3.1 –10 x ULN	>10 x ULN
Albumin – Hypoalbuminemia g/dL	2.8 – 3.1	2.5 – 2.7	< 2.5	--
Total Protein – Hypoproteinemia g/dL	5.5 – 6.0	5.0 – 5.4	< 5.0	--
Alkaline phosphate – increase by factor	1.1 – 2.0 x ULN	2.1 – 3.0 x ULN	3.1 – 10 x ULN	>10 x ULN
Liver Function Tests –ALT, AST increase by factor	1.1 – 2.5 x ULN	2.6 – 5.0 x ULN	5.1 – 10 x ULN	>10 x ULN
Bilirubin – when accompanied by any increase in Liver Function Test increase by factor	1.1 – 1.25 x ULN	1.26 – 1.5 x ULN	1.51 – 1.75 x ULN	>1.75 x ULN

Bilirubin – when Liver Function Test is normal; increase by factor	1.1 – 1.5 x ULN	1.6 – 2.0 x ULN	2.0 – 3.0 x ULN	>3.0 x ULN
Cholesterol	201 – 210	211 – 225	> 226	---
Pancreatic enzymes – amylase, lipase	1.1 – 1.5 x ULN	1.6 – 2.0 x ULN	2.1 – 5.0 x ULN	>5.0 x ULN

* The laboratory values provided in the tables serve as guidelines and are dependent upon institutional normal parameters. Institutional normal reference ranges should be provided to demonstrate that they are appropriate.

** The clinical signs or symptoms associated with laboratory abnormalities might result in characterization of the laboratory abnormalities as potentially life-threatening (Grade 4). For example. a low sodium value that falls within a Grade 3 parameter (125-129 mEq/L) should be recorded as a Grade 4 hyponatremia event if the participant had a new seizure associated with the low sodium value.

*** ULN is the upper limit of the normal range.

**Hematology***	**Mild (Grade 1)**	**Moderate (Grade 2)**	**Severe (Grade 3)**	**Potentially Life Threatening (Grade 4)**
Hemoglobin (Female) - gm/dL	11.0 – 12.0	9.5 – 10.9	8.0 – 9.4	<8.0
Hemoglobin (Female) change from baseline value - gm/dL	Any decrease – 1.5	1.6 – 2.0	2.1 – 5.0	>5.0
Hemoglobin (Male) - gm/dL	12.5 – 13.5	10.5 – 12.4	8.5 – 10.4	<8.5
Hemoglobin (Male) change from baseline value – gm/dL	Any decrease – 1.5	1.6 – 2.0	2.1 – 5.0	>5.0
WBC Increase - cell/mm3	10,800 – 15,000	15,001 – 20,000	20,001 – 25, 000	>25,000
WBC Decrease - cell/mm3	2,500 – 3,500	1,500 – 2,499	1,000 – 1,499	<1,000
Lymphocytes Decrease - cell/mm3	750 – 1,000	500 – 749	250 – 499	<250
Neutrophils Decrease - cell/mm3	1,500 – 2,000	1,000 – 1,499	500 – 999	<500
Eosinophils - cell/mm3	650 – 1500	1501 - 5000	> 5000	Hypereosinophilic
Platelets Decreased - cell/mm3	125,000 – 140,000	100,000 – 124,000	25,000 – 99,000	<25,000
PT – increase by factor (prothrombin time)	1.0 – 1.10 x ULN**	1.11 – 1.20 x ULN	1.21 – 1.25 x ULN	>1.25 ULN
PTT – increase by factor (partial thromboplastin time)	1.0 – 1.2 x ULN	1.21 – 1.4 x ULN	1.41 – 1.5 x ULN	> 1.5 x ULN
Fibrinogen increase - mg/dL	400 – 500	501 – 600	> 600	--
Fibrinogen decrease - mg/dL	150 – 200	125 – 149	100 – 124	<100 or associated with gross bleeding or disseminated intravascular coagulation (DIC)

* The laboratory values provided in the tables serve as guidelines and are dependent upon institutional normal parameters. Institutional normal reference ranges should be provided to demonstrate that they are appropriate.

** ULN is the upper limit of the normal range.

Urine *	Mild (Grade 1)	Moderate (Grade 2)	Severe (Grade 3)	Potentially Life-Threatening (Grade 4)
Protein	Trace	1+	2+	Hospitalization or dialysis
Glucose	Trace	1+	2+	Hospitalization for hyperglycemia
Blood (microscopic) – red blood cells per high power field (rbc/hpf)	1 - 10	11 – 50	> 50 and/or gross blood	Hospitalization or packed red blood cells (PRBC) transfusion

* The laboratory values provided in the tables serve as guidelines and are dependent upon institutional normal parameters. Institutional normal reference ranges should be provided to demonstrate that they are appropriate.

10.7. Appendix 7: Summary of Guidance from CDC Website[a] on Underlying Medical Conditions That Lead or Might Lead to Increased Risk for Severe Illness From COVID-19

Adults of any age with **certain underlying medical conditions** are at increased risk for severe illness from COVID-19: Severe illness from COVID-19 is defined as hospitalization, admission to the ICU, intubation or mechanical ventilation, or death.

Adults of any age with the following conditions can be more likely to get severely ill from COVID-19[b]:

- Cancer
- Chronic kidney disease
- Chronic lung diseases, including COPD (chronic obstructive pulmonary disease), asthma (moderate to severe), interstitial lung disease, cystic fibrosis, and pulmonary hypertension
- Dementia or other neurological conditions
- Diabetes (Type 1 and Type 2)
- Down syndrome
- Heart conditions (such as heart failure, coronary artery disease, cardiomyopathies, or hypertension)
- HIV infection
- Immunocompromised state (weakened immune system)
- Liver disease
- Overweight (BMI of >25 kg/m^2 but <30 kg/m^2), obesity (BMI of ≥30 kg/m^2 but <40 kg/m^2), or severe obesity (BMI ≥40 kg/m^2)
- Pregnancy
- Sickle cell disease or thalassemia
- Smoking (current and former)
- Solid organ or blood stem cell transplant
- Stroke or cerebrovascular disease, which affects blood flow to the brain
- Substance use disorders

[a]Source: https://www.cdc.gov/coronavirus/2019-ncov/need-extra-precautions/people-with-medical-conditions.html?CDC AA refVal=https%3A%2F%2Fwww.cdc.gov%2 Fcoronavirus% 2F2019-ncov%2Fneed-extra-precautions%2Fgroups-at-higher-risk.html. Updated 13 May 2021. Accessed: 8 June 2021.

[b] The list of underlying medical conditions is not exhaustive and only includes conditions with sufficient evidence to draw conclusions.

10.8. Appendix 8: TTS AESI Form

The form below represents the type of information that may be collected in case of a suspected AESI in order to help adjudicate whether the event is a case of TTS. Additional data may be requested by the sponsor for investigation of the event.

Adverse Event of Special Interest Questionnaire (AESIQ) for Thromboembolism with Thrombocytopenia Syndrome

Date of Report: [dd-MMM-yyyy]

1. Adverse Event Description

Participant's clinical signs and symptoms

☐ Leg/Calf Oedema	☐ Pain in Leg/Calf	☐ Haemoptysis
☐ Dyspnoea	☐ Chest Pain/Discomfort	☐ Syncope
☐ Tachypnoea	☐ Tachycardia	☐ Cough
☐ Loss of consciousness	☐ Headache	☐ Seizure
☐ Visual impairment	☐ Weakness	☐ Impaired speech
☐ Confusional state	☐ Paresthesia	☐ Gait disturbance

☐ Other symptoms:

Was patient on VTE prophylaxis? ☐ No ☐ Yes, details:

2. Medical History and Concurrent Conditions

Provide details:

Is the participant overweight or have obesity?	☐ No	☐ Yes
If available, please provide: Height	Weight	BMI
Does the participant have a sedentary lifestyle[a]?	☐ No	☐ Yes – details:
Has the participant been in a sitting position for long periods of time prior to the event?	☐ No	☐ Yes – details:
Is there a current history of smoking (active or passive)?	☐ No	☐ Yes – details:
Is there a prior history of smoking (active or passive)?	☐ No	☐ Yes – details:

Does the participant have a prior history of:

Cancer	☐ No	☐ Yes – details:
Autoimmune disease (i.e., collagen-vascular disease, inflammatory bowel disease) or myeloproliferative disease?	☐ No	☐ Yes – details:
Clotting disorder or a hypercoagulable state	☐ No	☐ Yes – details:
Varicose veins	☐ No	☐ Yes – details:
Trauma to the involved leg or pelvis	☐ No	☐ Yes – details:
DVT/PE or other VTE	☐ No	☐ Yes – details:
Blood transfusion	☐ No	☐ Yes – details:
Cardiovascular disease	☐ No	☐ Yes – details:

[a] Any waking behavior characterized by an energy expenditure less than or equal to 1.5 metabolic equivalents (METs), while in a sitting, reclining or lying posture

If the participant has experienced a previous thrombotic event, address the following:
1. Date (or estimate) 2. Provide brief description of the nature of the event 3. Provide brief description of the treatment of the event 4. Note any residual manifestations of the event.

If the patient has experienced more than one previous thrombotic event, please list other events.

Was the (female) participant pregnant at the time of event? ☐ No ☐ Yes – details:

Does the participant has any of genetic risk factors:

☐ Dysfibrinogenemia ☐ Antiphospholipid syndrome ☐ Factor V Leiden mutation
☐ Protein C or S deficiency ☐ Elevated factor VIII levels ☐ Anti-thrombin deficiency
☐ Hyperhomocysteinemia ☐ Prothrombin gene mutation ☐ Blood-clotting disorder
☐ Thrombophilia

Does the participant have any acquired risk factors:

☐ Reduced mobility (paralysis, paresis, travel etc.) ☐ Recent surgery
☐ Indwelling central venous catheters ☐ Recent trauma
☐ Recent discontinuation of anticoagulants (e.g., heparin, warfarin, DOACs)
☐ Hormone replacement therapy (including contraceptives)
☐ Phlebitis ☐ Lupus
☐ Inflammatory bowel disease ☐ Myeloproliferative disorders
☐ Diabetes mellitus ☐ Hyperlipidemia
☐ Hypertension ☐ Dehydration
☐ Other significant medical co-morbidities or risk factors for DVT, specify:

If yes to any of the above, provide details:
Provide Well's score, if calculated:

3. **Relevant results of diagnostic tests including laboratory tests, imaging, biopsies, etc.** (Note the levels/conclusion, date performed, **normal ranges** as well as any other details. **Alternatively, attach full reports of the diagnostic tests.)**

Diagnostic Test	Results at baseline or prior to use of product (Include date and value/details)	Test results after use of product (Include date and value/details)
CBC with smear (microscopic evaluation)		
ESR		
Platelet count		
Antibodies to platelet factor 4 (PF4)		
Fibrinogen levels		
Clauss fibrinogen assay		

Diagnostic Test	Results at baseline or prior to use of product (Include date and value/details)	Test results after use of product (Include date and value/details)
D-Dimer		
Clotting Profile (PT, aPTT- prior to an anticoagulation treatment)		
Thrombin time (Bovine) Plasma		
Prothrombin		
Antithrombin activity		
Factor V Leiden		
Protein C activity		
Protein S activity		
C-reactive protein		
Homocystein levels		
Dilute Russells Viper Venom Time (DRVVT), Plasma		
Activated Protein C Resistance V (APCRV), Plasma		
Thrombophilia interpretation		
Anticardiolipin antibodies (IgG and IgM) or beta-2 glycoproteins antibodies		
Antiphospholipid antibodies (IgG and IgM)		
Lupus anticoagulant		
Heparin antibodies		
ANA and ANCA		
IL6 levels		
ADAMTS13 Activity Assay		
Ceruloplasmin		
Direct Coombs test		
Complement C3, C4		
MethylenetetraHydrofolate reductase gene mutation		
Prothrombin gene mutation (G20210A)		
Occult blood in stool		
COVID-19 test		
Troponins		
Brain Natriuretic Peptide		

Diagnostic Test	Results at baseline or prior to use of product (Include date and value/details)	Test results after use of product (Include date and value/details)
Arterial Blood Gases		
Chest X-Ray		
Electrocardiography		
Echocardiography		
Duplex Ultrasonography		
MRI scan		
CT scan		
Contrast Venography		
Pulmonary Angiography		
Ventilation-Perfusion Scanning		

Provide details of any additional diagnostic results:

10.9. Appendix 9: Thrombotic Events to be Reported as AESIs

At the time of protocol writing, the list of thrombotic events to be reported to the sponsor as AESIs is provided below. Further guidance may become available on thrombotic events of interest.

- MedDRA PTs for large vessel thrombosis and embolism:
  - Aortic embolus, aortic thrombosis, aseptic cavernous sinus thrombosis, brain stem embolism, brain stem thrombosis, carotid arterial embolus, carotid artery thrombosis, cavernous sinus thrombosis, cerebral artery thrombosis, cerebral venous sinus thrombosis, cerebral venous thrombosis, superior sagittal sinus thrombosis, transverse sinus thrombosis, mesenteric artery embolism, mesenteric artery thrombosis, mesenteric vein thrombosis, splenic artery thrombosis, splenic embolism, splenic thrombosis, thrombosis mesenteric vessel, visceral venous thrombosis, hepatic artery embolism, hepatic artery thrombosis, hepatic vein embolism, hepatic vein thrombosis, portal vein embolism, portal vein thrombosis, portosplenomesenteric venous thrombosis, splenic vein thrombosis, spontaneous heparin-induced thrombocytopenia syndrome, femoral artery embolism, iliac artery embolism, jugular vein embolism, jugular vein thrombosis, subclavian artery embolism, subclavian vein thrombosis, obstetrical pulmonary embolism, pulmonary artery thrombosis, pulmonary thrombosis, pulmonary venous thrombosis, renal artery thrombosis, renal embolism, renal vein embolism, renal vein thrombosis, brachiocephalic vein thrombosis, vena cava embolism, vena cava thrombosis, truncus coeliacus thrombosis

- MedDRA PTs for more common thrombotic events:
  - Axillary vein thrombosis, deep vein thrombosis, pulmonary embolism, MedDRA PTs for acute myocardial infarction*, MedDRA PTs for stroke*

Source: Shimabukuro T. CDC COVID-19 Vaccine Task Force. Thrombosis with thrombocytopenia syndrome (TTS) following Janssen COVID-19 vaccine. Advisory Committee on Immunization Practices (ACIP). April 23, 2021. https://www.cdc.gov/vaccines/acip/meetings/slides-2021-04-23.html.

*Vaccine Adverse Event Reporting System (VAERS) Standard Operating Procedures for COVID-19 (as of 29 January 2021) https://www.cdc.gov/vaccinesafety/pdf/VAERS-v2-SOP.pdf

10.10. Appendix 10: Protocol Amendment History

This is an original protocol.

11. REFERENCES

1. Agrawal AS (2016), Tao X, Algaissi A, et al. Immunization with inactivated Middle East Respiratory Syndrome coronavirus vaccine leads to lung immunopathology on challenge with live virus. Hum Vaccin Immunother. 2016;12(9):2351-2356.

2. American Society of Hematology (2021). COVID-19 resources. Thrombosis with Thrombocytopenia Syndrome (also termed Vaccine-induced Thrombotic Thrombocytopenia). https://www.hematology.org/covid-19/vaccine-induced-immune-thrombotic-thrombocytopenia. Last updated: 29 April 2021. Accessed: 02 July 2021.

3. Anywaine Z (2019), Whitworth H, Kaleebu P, et al. Safety and immunogenicity of a 2-dose heterologous vaccination regimen with Ad26.ZEBOV and MVA-BN-Filo Ebola vaccines: 12-month data from a Phase 1 randomized clinical trial in Uganda and Tanzania. J Infect Dis. 2019;220(1):46-56.

4. Arepally GM (2021), Padmanabhan, A. Heparin-induced thrombocytopenia: a focus on thrombosis. Arterioscler Thromb Vasc Biol. 2021;41:141–152.

5. Baden LR (2020), El Sahly HM, Essink B, et al. Efficacy and safety of the mRNA-1273 SARS-CoV-2 vaccine. N Engl J Med. 2021;384:403-416.

6. Barouch DH (2013), Liu J, Peter L, et al. Characterization of humoral and cellular immune responses elicited by a recombinant adenovirus serotype 26 HIV-1 Env vaccine in healthy adults (IPCAVD 001). J Infect Dis. 2013:207(2);248-256.

7. Barouch DH (2018), Tomaka FL, Wegmann F, et al. Evaluation of a mosaic HIV-1 vaccine in a multicentre, randomised, double-blind, placebo-controlled, phase 1/2a clinical trial (APPROACH) and in rhesus monkeys (NHP 13-19). Lancet. 2018;392(10143):232-243.

8. Bartoszko J (2021), Loeb M. The burden of influenza in older adults: meeting the challenge. Aging Clin Exp Res. 2021;33(3):711-717.

9. Bolles M (2011), Deming D, Long K, et al. A double-inactivated severe acute respiratory syndrome coronavirus vaccine provides incomplete protection in mice and induces increased eosinophilic proinflammatory pulmonary response upon challenge. J Virol. 2011;85(23):12201-12215.

10. Brighton Collaboration (2021). Updated Proposed Brighton Collaboration process for developing a standard case definition for study of new clinical syndrome X, as applied to Thrombosis with Thrombocytopenia Syndrome (TTS). 18 May 2021. https://brightoncollaboration.us/wp-content/uploads/2021/05/TTS-Interim-Case-Definition-v10.16.3-May-23-2021.pdf.Accessed: 02 September 2021.

11. British Society for Haematology (2021). Guidance produced from the Expert Haematology Panel (EHP) focussed on Covid-19 Vaccine induced Thrombosis and Thrombocytopenia (VITT). Version 1.3; 7 April 2021. https://b-s-h.org.uk/media/19530/guidance-version-13-on-mngmt-of-thrombosis-with-thrombocytopenia-occurring-after-c-19-vaccine 20210407.pdf. Accessed: 02 July 2021.

12. Brosio F (2018), D'Alò GL, Baccello V, Terracciano E, Franco E, Gabutti G. Vaccine co-administration in clinical practice. Ig Sanita Pubbl. 2018;74(5):475-494.

13. Centers for Disease Control and Prevention (2020). COVID-19 Guidance for Older Adults. https://www.cdc.gov/aging/covid19-guidance.html. Accessed: 02 July 2021.

14. Centers for Disease Control and Prevention (2021). Cases of cerebral venous sinus thrombosis with thrombocytopenia after receipt of the Johnson & Johnson COVID-19 Vaccine. 13 April 2021. https://emergency.cdc.gov/han/2021/han00442.asp. Accessed: 02 July 2021.

15. Centers for Disease Control and Prevention (2021a). Coronavirus Disease 2019 (COVID-19) Groups at Higher Risk for Severe Illness. https://www.cdc.gov/coronavirus/2019-ncov/need-extra-precautions/groups-at-higher-risk.html. Updated: 29 March 2021. Accessed: 04 June 2021.

16. Centers for Disease Control and Prevention (2021b). Seasonal influenza-associated hospitalizations in the United States. https://www.cdc.gov/flu/about/burden/faq.htm (page last reviewed: 04 June 2021). Accessed 24 June 2021.

17. Centers for Disease Control and Prevention (2021c). COVID-19 vaccines – Coadministration with other vaccines. https://www.cdc.gov/vaccines/covid-19/clinical-considerations/covid-19-vaccines-us.html?CDC AA refVal=https%3A%2F%2Fwww.cdc.gov%2Fvaccines%2Fcovid-19%2Finfo-by-product%2Fclinical-considerations.html#Coadministration. Accessed: 04 June 2021.

18. Deming D (2006), Sheahan T, Heise M, et al. Vaccine efficacy in senescent mice challenged with recombinant SARS-CoV bearing epidemic and zoonotic spike variants. PLoS Med. 2006;3(12):e525.

19. European Centre for Disease Prevention and Control (2021). COVID-19 country overviews. https://www.ecdc.europa.eu/en/cases-2019-ncov-eueea. Accessed 11 July 2021.

20. European Commission (1998) 98/463/EC: Council Recommendation of 29 June 1998 on the suitability of blood and plasma donors and the screening of donated blood in the European Community.

21. European Medicines Agency (2021). Vaxzevria: EMA advises against use in people with history of capillary leak syndrome. https://www.ema.europa.eu/en/news/vaxzevria-ema-advises-against-use-people-history-capillary-leak-syndrome. Published: 11 June 2021. Accessed: 01 July 2021.

22. Gidudu JF (2012), Walco GA, Taddio A, et al./The Brighton Immunization Site Pain Working Group. Immunization site pain: case definition and guidelines for collection, analysis, and presentation of immunization safety data. Vaccine. 2012:30(30);4558-4577.

23. He X (2021), Chandrashekar A, Zahn R, et al. Low-dose Ad26.COV2.S protection against SARS-CoV-2 challenge in rhesus macaques. BioRxiv 2021; doi:10.1101/2021.01.27.428380. [preprint]

24. Honda-okubo Y (2015), Barnard D, Ong CH, Peng BH, Tseng CT, Petrovsky N. Severe acute respiratory syndrome-associated coronavirus vaccines formulated with delta inulin adjuvants provide enhanced protection while ameliorating lung eosinophilic immunopathology. J Virol. 2015;89(6):2995-3007.

25. Houser KV (2017), Broadbent AJ, Gretebeck L, et al. Enhanced inflammation in New Zealand white rabbits when MERS-CoV reinfection occurs in the absence of neutralizing antibody. PLoS Pathog. 2017;13(8):e1006565.

26. Huang K (2021), Lin SW, Sheng WH, Wang CC. Influenza vaccination and the risk of COVID-19 infection and severe illness in older adults in the United States. Sci Rep 2021;11(1):11025.

27. Investigator's Brochure (2021): Ad26.COV2.S (VAC31518), Edition 5.0. Janssen Vaccines & Prevention B.V (December 2021). Janssen Vaccines & Prevention B.V. Data on file.

28. Johns Hopkins CSSE (2021). Coronavirus COVID-19 Global Cases. https://www.arcgis.com/apps/dashboards/bda7594740fd40299423467b48e9ecf6. Accessed 11 July 2021.

29. Kohl KS (2007), Walop W, Gidudu J, et al./The Brighton Collaboration Local Reaction Working Group for Swelling at or near Injection Site. Swelling at or near injection site: case definition and guidelines for collection, analysis and presentation of immunization safety data. Vaccine 2007:25(31);5858-5874.

30. Lee GM (2021). CDC COVID-19 Vaccine Safety Technical (VaST) Work Group. Advisory Committee on Immunization Practices (ACIP). May 12, 2021. https://www.cdc.gov/vaccines/acip/meetings/downloads/slides-2021-05-12/08-COVID-Lee-508.pdf.

31. MacNeil JR (2021), Su JR, Broder KR, et al. Updated Recommendations from the Advisory Committee on Immunization Practices for Use of the Janssen (Johnson & Johnson) COVID-19 Vaccine After Reports of Thrombosis with Thrombocytopenia Syndrome Among Vaccine Recipients — United States, April 2021. MMWR Morb Mortal Wkly Rep 2021;70:651-656.

32. Mertz D (2013), Kim TH, Johnstone J, et al. Populations at risk for severe or complicated influenza illness: systematic review and meta-analysis. BMJ. 2013;347(aug23 1):f5061-f5061.

33. Milligan ID (2016), Gibani MM, Sewell R, et al. Safety and immunogenicity of novel adenovirus type 26- and modified vaccinia ankara-vectored ebola vaccines: a randomized clinical trial. JAMA. 2016:315(15);1610-1623.

34. Mutua G (2019), Anzala O, Luhn K, et al. Safety and immunogenicity of a 2-dose heterologous vaccine regimen with Ad26.ZEBOV and MVA-BN-Filo Ebola vaccines: 12-month data from a Phase 1 randomized clinical trial in Nairobi, Kenya. J Infect Dis. 2019;220(1):57-67.

35. Perrotta F (2020), Corbi G, Mazzeo G, et al. COVID-19 and the elderly: insights into pathogenesis and clinical decision-making. Aging Clin Exp Res. 2020;32(8):1599-1608.

36. Polack FP (2020), Thomas SJ, Kitchin N, et al. Safety and efficacy of the BNT162b2 mRNA COVID-19 vaccine. N Engl J Med. 2020;383(27):2603-2615.

37. Rüggeberg JU (2007), Gold MS, Bayas J, et al. Anaphylaxis: case definition and guidelines for data collection, analysis, and presentation of immunization safety data. Vaccine; 2007;25(31):5675-5684.

38. Sadoff J (2020), De Paepe E, Haazen W, et al. Safety and immunogenicity of the ad26. Rsv. Pref investigational vaccine coadministered with an influenza vaccine in older adults. The Journal of Infectious Diseases. 2021;223(4):699-708.

39. Sadoff J (2021), Le Gars M, Shukarev G, et al. Interim Results of a Phase 1-2a Trial of Ad26.COV2.S Covid-19 Vaccine. N Engl J Med. Published online January 13, 2021:NEJMoa2034201.

40. Salisch NC (2019), Izquierdo Gil A, Czapska-Casey DN, et al. Adenovectors encoding RSV-F protein induce durable and mucosal immunity in macaques after two intramuscular administrations. NPJ Vaccines. 2019;4:54.

41. Salisch NC (2021), Stephenson KE, Williams K, et al. A double-blind, randomized, placebo-controlled Phase 1 study of Ad26.ZIKV.001, an Ad26-vectored anti-zika virus vaccine. Ann Intern Med. 2021;174(5):585-594.

42. Shimabukuro T (2021). CDC COVID-19 Vaccine Task Force. Thrombosis with thrombocytopenia syndrome (TTS) following Janssen COVID-19 vaccine. Advisory Committee on Immunization Practices (ACIP). April 23, 2021. https://www.cdc.gov/vaccines/acip/meetings/slides-2021-04-23.html.

43. Streiff MB (2021). Pathogenesis and Management of Thrombosis with Thrombocytopenia Syndrome (TTS). Centers for Disease Control & Prevention Advisory Committee on Immunization Practices (ACIP) meeting 23 April 2021. https://www.cdc.gov/vaccines/acip/meetings/downloads/slides-2021-04-23/02-COVID-Strieff-508.pdf. (accessed 18 May 2021).

44. Toback S (2021), Galiza E, Cosgrove C, et al. Safety, immunogenicity, and efficacy of a COVID-19 vaccine (NVX-CoV2373) co-administered with seasonal influenza vaccines. MedRxiv preprint doi: https://doi.org/10.1101/2021.06.09.21258556.

45. US Department of Health and Human Services (2007), Food and Drug Administration, Center for Biologics Evaluation and Research. September 2007. Guidance for Industry. Toxicity Grading Scale for Healthy Adult and Adolescent Volunteers Enrolled in Preventive Vaccine Clinical Trials. https://www.fda.gov/downloads/BiologicsBloodVaccines/GuidanceComplianceRegulatoryInformation/Guidances/Vaccines/ucm091977.pdf. Accessed 19 February 2020.

46. US Department of Health and Human Services (1998). Office for Human Research Protections - OHRP Expedited Review Categories. 1998. https://www.hhs.gov/ohrp/regulations-and-policy/guidance/categoriesof-research-expedited-review-procedure-1998/index.html. Accessed 18 September 2020.

47. US Food and Drug Administration (1998). Conditions for IRB Use of Expedited Review. Federal Register: November 9, 1998 (Volume 63, Number 216). https://www.fda.gov/science-research/guidance-documentsincluding-information-sheets-and-notices/conditions-irb-use-expedited-review. Accessed 24 February 2020.

48. US Food and Drug Administration (2020). Development and Licensure of Vaccines to Prevent COVID-19. Guidance for Industry. June 2020. https://www.fda.gov/media/139638/download. Accessed 2 April 2021.

49. US Food and Drug Administration (2021), Center for Biologics Evaluation and Research. May 2021. Guidance for Industry. Emergency Use Authorization for Vaccines to Prevent COVID-19. https://www.fda.gov/regulatory-information/search-fda-guidance-documents/emergency-use-authorization-vaccines-prevent-covid-19. Accessed 01 June 2021.

50. US Food and Drug Administration (2021a). COVID-19: Developing Drug and Biological Products for Treatment or Prevention. Guidance for Industry. February 2021. https://www.fda.gov/media/137926/download. Accessed 2 April 2021.

51. van der Fits L (2020), Bolder R, Heemskerk-van der Meer M, et al. Adenovector 26 encoded prefusion conformation stabilized RSV-F protein induces long-lasting Th1-biased immunity in neonatal mice. NPJ Vaccines. 2020;5:49.

52. van der Lubbe JEM (2021), Rosendahl Huber SK, Vijayan A, et al. Ad26.COV2.S-elicited immunity protects against G614 spike variant SARS2 CoV-2 infection in Syrian hamsters and does not enhance respiratory disease in challenged animals with breakthrough infection after sub-optimal vaccine dosing. Immunology; 2021; https://doi.org/10.1101/2021.01.08.425915.

53. Voysey M (2021), Clemens SAC, Madhi SA. Safety and efficacy of the ChAdOx1 nCoV-19 vaccine (AZD1222) against SARS-CoV-2: an interim analysis of four randomised controlled trials in Brazil, South Africa and the UK. Lancet.2021;397(10269):99-111.

54. Widjojoatmodjo MN (2015), Bogaert L, Meek B, et al. Recombinant low-seroprevalent adenoviral vectors Ad26 and Ad35 expressing the respiratory syncytial virus (RSV) fusion protein induce protective immunity against RSV infection in cotton rats. Vaccine. 2015; 33(41):5406-5414.

55. World Health Organization (2018). Influenza (Seasonal). 6 November 2018. https://www.who.int/en/news-room/fact-sheets/detail/influenza-(seasonal). Accessed 24 June 2020.

56. World Health Organization (2020). Coronavirus disease 2019 (COVID-19) Situation Report - 51. Available at: https://www.who.int/emergencies/diseases/novel-coronavirus-2019/situation-reports. Accessed: 02 July 2021.

57. World Health Organization (2020a). WHO Director-General's opening remarks at the media briefing on COVID-19 - 11 March 2020. https://www.who.int/dg/speeches/detail/who-director-general-s-opening-remarks-at-the-media-briefing-on-covid-19---11-march-2020. Accessed 02 July 2021.

58. Zhou H (2012), Thompson WW, Viboud CG, Ringholz CM, et al. Hospitalizations associated with influenza and respiratory syncytial virus in the United States, 1993-2008. Clin Infect Dis. 2012;54(10):1427-1436.

59. Zhou T (2004), Wang H, Luo D, et al. An exposed domain in the severe acute respiratory syndrome coronavirus spike protein induces neutralizing antibodies. J Virol. 2004;78(13):7217-7226.

60. Zumla A (2016), Chan JF, Azhar EI, Hui DS, Yuen KY. Coronaviruses - drug discovery and therapeutic options. Nat Rev Drug Discov. 2016;15(5):327-347.

INVESTIGATOR AGREEMENT

I have read this protocol and agree that it contains all necessary details for carrying out this study. I will conduct the study as outlined herein and will complete the study within the time designated.

I will provide copies of the protocol and all pertinent information to all individuals responsible to me who assist in the conduct of this study. I will discuss this material with them to ensure that they are fully informed regarding the study vaccine, the conduct of the study, and the obligations of confidentiality.

Coordinating Investigator (where required):

Name (typed or printed): ____________________

Institution and Address: ____________________

____

____

____

Signature: ____________________ Date: ____________________

(Day Month Year)

Principal (Site) Investigator:

Name (typed or printed): ____________________

Institution and Address: ____________________

____

____

____

Telephone Number: ____________________

Signature: ____________________ Date: ____________________

(Day Month Year)

Sponsor's Responsible Medical Officer:

Name (typed or printed): PPD

Institution: Janssen Vaccines & Prevention B.V.

Signature: electronic signature appended at the end of the protocol Date: ____________________

(Day Month Year)

Note: If the address or telephone number of the investigator changes during the study, written notification will be provided by the investigator to the sponsor, and a protocol amendment will not be required.

Signature

User	Date	Reason
PPD	15-Feb-2022 17:52:23 (GMT)	Document Approval
